# Comprehensive Profiling of Antioxidant, Antidiabetic, and Cytotoxic Compounds from *Morinda lucida* Benth Using ^1^H-NMR- and UHPLC-Q Exactive Orbitrap MS-Based Metabolomics Combined with Molecular Networking and Molecular Docking

**DOI:** 10.3390/metabo16080523

**Published:** 2026-07-24

**Authors:** Dorcas Tlhapi, Ntsoaki Malebo, Idah Tichaidza Manduna, Monizi Mawunu, Chika Ifeanyi Chukwuma, Ramakwala Christinah Chokwe, Kolawole Olofinsan

**Affiliations:** 1 Centre for Applied Food Sustainability and Biotechnology, Faculty of Health and Environmental Sciences, Central University of Technology, Bloemfontein 9301, South Africa; imanduna@cut.ac.za; 2 Department of Life Sciences, Faculty of Health and Environmental Sciences, Central University of Technology, Bloemfontein 9301, South Africa; nmalebo@cut.ac.za; 3 Department of Agronomy, Polytechnic Institute, Kimpa Vita University, Uíge P.O. Box 77, Angola; m.mawunu2000@gmail.com; 4 Department of Biology, Faculty of Science and Technology, University of Kinshasa, Kinshasa P.O. Box 190, Congo; 5 Centre for Quality of Health and Living, Faculty of Health and Environmental Sciences, Central University of Technology, Bloemfontein 9301, South Africa; cchukwuma@cut.ac.za; 6 Department of Chemistry, College of Science Engineering and Technology, University of South Africa, Florida, Johannesburg 1710, South Africa; chokwrc@unisa.ac.za; 7 Department of Pharmacology, Faculty of Health Sciences, University of the Free State, Bloemfontein 9300, South Africa; olofinsan.k@ufs.ac.za

**Keywords:** *Morinda lucida*, ^1^H-NMR, UHPLC-Q Exactive Orbitrap MS, molecular networking, molecular docking, phytochemical compounds, chemical profile, antioxidant activity, alpha-glucosidase inhibition, cytotoxicity

## Abstract

**Background/Objectives**: *Morinda lucida* Benth is widely distributed throughout Central and West Africa. It has traditionally been used to treat and manage various diseases. However, scientific research on its phytochemical and pharmacological properties remains scarce. This study investigated the phytochemical profiles, antioxidant activities, α-glucosidase inhibitors, and cytotoxic effects of compounds derived from various parts of *M. lucida*. **Methods**: Seventy-seven natural compounds were putatively annotated using ^1^H-NMR, UHPLC–Q Exactive Orbitrap MS, and molecular networking techniques. The antioxidant activities of the crude extracts were assessed in vitro using DPPH free radical scavenging and reducing power assays, whereas the in vitro α-glucosidase inhibition activity and toxicity of the crude extracts were evaluated using the α-glucosidase inhibition and MTT assays. Molecular docking was used to assess the interactions between the identified glycosides and the α-glucosidase protein. **Results**: The root extract exhibited the highest DPPH free radical scavenging (IC_50_ = 7.7550 ± 6.9142 μg/mL) and reducing power capacity (IC_0.5_ = 0.0052 ± 0.0025 μg/mL). In contrast, the stem bark extract demonstrated significant inhibition of alpha-glucosidase (IC_50_ = 79.9 ± 16.1 µg/mL). Sophoricoside, formononetin 7-O-glucoside, and epicatechin identified in the stem bark extract showed notable in silico interactions with the α-glucosidase protein. The stem bark and leaf extracts were more toxic than the root extract at different concentrations. **Conclusions**: The results of this study demonstrate the therapeutic potential of *M. lucida* and provide information on its phytochemical composition and pharmacological properties.

## 1. Introduction

*Morinda lucida* Benth (“brimstone tree”) is a medicinal plant species belonging to the Rubiaceae family, which includes approximately 12,000 known species and approximately 576 genera, and is widely distributed in Central and West Africa [1,2]. *M. lucida* has been used to treat various ailments, including diabetes, hypertension, cancer, inflammation, parasitic worms, malaria, jaundice, trypanosomiasis, typhoid fever, neurological disorders, cognitive disorders, fever, and sickle cell disease [2,3,4]. Several studies on *M. lucida* have revealed its bioactivities, including antiproliferative, antionchocercal, antifungal, antitrypanosomal, antileishmanial, antisickling, hypotensive, antidiabetic, anti-inflammatory, antimicrobial, immunostimulatory, antimalarial, and antioxidant activities [5]. Various compounds, including cardiac glycosides, polyphenols, iridoids, terpenoids, sterols, anthraquinones, flavonoids, saponins, fatty acids, phenols, alkaloids, and tannins, have been identified in different parts of *Morinda lucida* [6]. These compounds may be correlated with the medicinal properties of the plants. Several studies have been performed on the bioactivities of *M. lucida*; however, there has been no documentation of metabolomic profiles linked to the bioactivity of this plant species. Therefore, it is necessary to discover the phytochemicals that contribute to the antioxidant activity, α-glucosidase inhibition, and cytotoxic effects of this plant. Studies on *M. lucida* have focused on classical phytochemical techniques such as column chromatography (CC), Sephadex LH-20, liquid chromatography (LC), gas chromatography (GC), thin-layer chromatography (TLC), and high-performance thin-layer chromatography (HPTLC). However, these methods are time-consuming and often inefficient for the analysis of chemical constituents [7,8]. ^1^H nuclear magnetic resonance (^1^H-NMR) and ultra-high-performance liquid chromatography-quadrupole-electrostatic field orbitrap mass spectrometry (UHPLC–Q Exactive Orbitrap MS) combined with molecular networking (MN) and molecular docking techniques have modernized phytochemical analysis, allowing for faster identification, annotation, and quantification of different phytochemicals with remarkable sensitivity and precision [9].

Metabolomics involves methods and techniques to analyze a group of chemical compounds in a complex matrix [10,11,12]. Molecular networking is a tool used to classify, identify, and visualize the similarities between metabolites [13]. This approach makes it easier for scientific researchers to annotate known natural compounds in crude extracts and focus on unknown natural compounds that could be of biological interest [14]. Molecular docking is a computational technique used to analyze, model, and predict chemical and biological processes [15]. Few studies have shown that *M. lucida* is used to treat diabetes [1,15,16]; however, the bioactive compounds in this plant have rarely been isolated and characterized using classical phytochemical techniques. Furthermore, there are no reports on the chemical profiling of bioactive extracts from *Morinda* species for α-glucosidase inhibition using a metabolomic approach. Applying these techniques, the stem bark, root, and leaf extracts of *Morinda lucida* were analyzed to identify possible antidiabetic compounds. This study explored the metabolic fingerprint, in vitro antioxidant activity, α-glucosidase potential, and cytotoxicity of different plant parts of *M. lucida* using proton nuclear magnetic resonance (^1^H-NMR), ultra-high-performance liquid chromatography–quadrupole–electrostatic field orbitrap mass spectrometry (UHPLC–Q Exactive Orbitrap MS), MN, and molecular docking techniques. Furthermore, this study provides information on which plant organ can be a potential source of safe and effective compounds for the treatment of diabetes using the 2,2-diphenyl-1-picrylhydrazyl (DPPH) free radical scavenging, reducing power, α-glucosidase inhibition, and MTT (3-(4,5-dimethylthiazol-2-yl)-2,5-diphenyltetrazolium bromide) assays.

## 2. Materials and Methods

### 2.1. General Experimental Procedure

Minimal essential medium, fetal bovine serum, L- glutamine, penicillin/streptomycin, trypan blue, Earle’s balanced salt solution, and trypsin/EDTA were obtained from Cytiva HyClone (Marlborough, MA, USA). Acarbose, α-glucosidase enzyme solution, phosphate-buffered saline (PBS), and *p*-Nitrophenyl α-D-glucopyranoside were purchased from Sigma-Aldrich (Darmstadt, Germany). African green monkey (Vero) kidney cells (ATCC CCL-81, RRID: CVCL_0059) were purchased from Cytiva HyClone (Marlborough, MA, USA). Acetonitrile, methanol, dichloromethane, and formic acid were purchased from Sigma-Aldrich (St. Louis, MO, USA). Ascorbic acid, quercetin, gallic acid, DPPH (2,2-diphenyl-1-picrylhydrazyl), and dimethyl sulfoxide were purchased from Sigma-Aldrich (Darmstadt, Germany). All solvents and chemicals used in this research study were of analytical grade.

### 2.2. Plant Collection, Sampling, and Extraction

The leaves, root, and stem bark of *Morinda lucida* were collected in Condo Benza, Uíge Province, Angola (Latitude: S 7°35′59.38044″, Longitude: E 15°0′16.434″, https://www.google.com/maps/search/?api=1&query=-7.5998279,15.004565) accessed on 15 March 2023. The samples were air-dried for four weeks and then ground to a fine powder using a POLYMIX lab mill with blade grinding, PX- MFC 90 D (Willows, Bloemfontein, South Africa). The samples were identified, and a herbarium reference specimen (UNIKIVI-017-2023) was deposited at Kimpa Vita University, Uíge Province, Angola. The dried leaves (approximately 115.32 g) of *M. lucida* were extracted by maceration with 2 L of 100% dichloromethane and 100% methanol at room temperature for 48 h. The resulting crude dichloromethane and methanol extracts were filtered and concentrated using a Büchi Rotavapor (Sigma-Aldrich, St. Louis, MO, USA) to 1.32 g and 4.00 g of dichloromethane and methanol leaf extracts, respectively, were obtained. In addition, the dried root (approximately 859.07 g) and stem bark (approximately 173.76 g) of *M. lucida* were extracted by maceration with 2 L of 100% methanol at room temperature for 48 h. The resulting crude methanol extracts were filtered and concentrated using a Büchi Rotavapor (Sigma-Aldrich, St. Louis, MO, USA) to 8.12 g and 9.45 g of methanol root and stem bark extracts.

### 2.3. ^1^H-NMR Spectroscopic Analysis

One-dimensional (1D) ^1^H-NMR spectra were recorded at 500 MHz using an Avance III HD spectrometer (Bruker, Fällanden, Switzerland). The chemical shifts for ^1^H-NMR were measured in parts per million (ppm). Deuterated chloroform (CDCl_3_) and dimethyl sulfoxide-d6 (DMSO-d6) served as solvents for preparation of the NMR samples. The NMR spectral data were processed using Bruker TopSpin 5.0 software, which facilitated baseline correction, phase correction, normalization, and referencing. All the crude extracts underwent analysis via ^1^H-NMR spectroscopy, and the annotation of compounds was confirmed through one-dimensional (1D) ^1^H-NMR spectral analysis, comparison with previously reported 1D ^1^H-NMR data, and reference to the Human Metabolite Database (https://hmdb.ca).

### 2.4. UHPLC–Q Exactive Orbitrap MS Analysis

The leaves, root, and stem bark extracts of *Morinda lucida* were annotated using a Q Exactive Plus Orbitrap mass spectrometer connected to a Thermo Scientific Dionex Ulti-mate 3000 ultra-high-performance liquid chromatography system (UHPLC; Thermo Fisher Scientific, Waltham, MA, USA). The Exactive Plus was attached to the mass spectrometry with a heated electrospray ionization (ESI) probe and an optimum source (auxiliary temperature, 400 °C; spray voltage, 3 kV; 50 arbitrary units; sheath gas flow, capillary temperature, 290 °C). Full mass spectrometry (MS) selected ion monitoring and data-dependent MS^2^ with both negative and positive polarity exchanging over a scan range of *m*/*z* 100 to 1500 with a mass tolerance window of less than 5 ppm were used for both the negative and positive ESI modes. The mass spectrometer was operated at a resolution of 70,000 full width at half maximum in a complete scan mode, with an automated gain control target set at 1.0 × 10^6^ and a maximum injection time of 100 ms. Liquid chromatography, electrospray ionization, and Orbitrap mass spectrometry (LC-ESI-Orbitrap-MS) analysis was performed using a C18 analytical column (4.6 × 150 mm, particle size 3.5 µm), and the mobile phases used were formic acid (0.1% *v*/*v*) in solvent A (water) and formic acid (0.1% *v*/*v*) in solvent B (acetonitrile). Linear gradient elution began at 0 min with 5% solvent B and increased to 20 min with 100% solvent B. The sample injection volume was 10 µL, mobile phase flow rate was 0.9 mL min^−1^, and column temperature was 25 °C. Data processing was performed using XCaliber version 3.0 (Thermo Fisher Scientific Inc., Waltham, MA, USA). Both electrospray ionization modes, namely negative (ESI−, Figure S2) and positive (ESI+, Figure S1), were evaluated; however, the negative electrospray ionization mode was selected for subsequent sample analysis due to its higher ion production compared with the positive mode. Raw data files were converted into mzXML format using the ProteoWizard tool MSConvertGUI software (version 3.0.24164-38d6037). The mzXML files were analyzed using MZmine (version 3.9.0). The aligned peak list, which included the retention time (tR), peak heights, and *m*/*z* values for each sample, was subsequently converted to the -quant.csv format. These converted files were uploaded to Mass Bank (https://massbank.eu), an online database dedicated to the annotation of compounds. Secondary metabolites with a matching score greater than 80% in the database were considered, and the quantified and identified secondary metabolites were extracted from the sample chromatogram data for validation. A data-dependent scan (dd-MS^2^) was employed to obtain high-quality MS/MS data, wherein the top five most intense precursors were automatically selected for MS/MS fragmentation through higher-energy collisional dissociation (HCD). The parameters of dd-MS^2^ were as follows: resolution, 17,500; automatic gain control target, 1 × 10^5^; Maximum IT, 50 ms; loop count, 5; isolation window, 4 *m*/*z*. The Normalized Collision Energy was set to 20, 40, and 60 V.

### 2.5. Global Natural Product Social Molecular Networking (GNPS) and Metabolite Annotation

The analysis of the *Morinda lucida* crude extracts was performed using the molecular networking approach, as outlined on the Global Natural Product Social molecular networking website (http://gnps.ucsd.edu, accessed on 7 July 2025). The mass spectroscopic data were converted into the mzXML format using the ProteoWizard tool MSConvertGUI (version 3.0.24164-38d6037) software. Subsequently, the spectral data (mzXML files) were uploaded to Global Natural Product Social molecular networking platform via WinSCP (version 6.3.4 (Build 14955 17 June 2024)) and analyzed using the GNPS platform (http://gnps.ucsd.edu, accessed on 7 July 2025). Following the upload of the spectral files, molecular networking was employed to connect the mass spectra of the compounds based on the similarity of their MS/MS fragment ions. The optimum parameters were as follows: the precursor ion mass tolerance was set to 0.02 Da, and fragment ion mass tolerance was set to 0.02 Da. Molecular networks were constructed with a cosine score exceeding 0.6 and a minimum of four matched fragment ions shared by at least one MS^2^ spectrum. The results were downloaded, and the generated spectral network was uploaded to Cytoscape (Version 3.91) software for network visualization [17]. Chemical structures were illustrated using ChemDraw 15.0. For compound annotations, the empirical formulas derived from the exact mass attained from the MS^2^ data of the compounds were utilized to annotate both unmatched and matched nodes, which were then compared with other established natural product dereplication databases, such as the Human Metabolome Database (https://hmdb.ca, accessed on 4 August 2025), the Dictionary of Natural Products (http://dnp.chemnetbase.com/faces/chemical/ChemicalSearch.xhtml, accessed on 8 September 2025), ChemSpider (www.chemspider.com, accessed on 6 October 2025), MassBank (https://massbank.eu, accessed on 10 November 2025), and KNApSAck (www.knapsackfamily.com, accessed on 2 December 2025).

### 2.6. Antioxidant Assay

#### 2.6.1. Free Radical Scavenging Assay (DPPH)

The antioxidant activity was assessed using the DPPH free radical scavenging method outlined by Mintsa et al. (2022) [18]. One hundred microliters of the leaf, root, and stem bark crude extracts were added in triplicate (n = 3) to the first three wells of a 96-well plate containing 100 µL of distilled water, followed by a two-fold serial dilution. Subsequent to the serial dilutions, 200 µL of DPPH/methanol solution (0.3 M) was introduced into all the wells of the plate. The plates were incubated at room temperature for 30 min, after which the absorbance was measured using a SoftMaxR Pro 6 (version 6.3) microplate reader at a wavelength of 517 nm. Gallic acid, quercetin, and ascorbic acid served as positive controls, which were also tested in triplicate.

DPPH inhibition percentage was calculated using the following Formula (1):

(1) $$DPPH\;RSA\;\left(\%\right)=\;\frac{Absorbance\;of\;DPPH\;-\;Absorbance\;of\;sample}{Absorbance\;of\;DPPH}\;\;\times \;100\;$$

#### 2.6.2. Reducing Power Assay

The reducing power of the samples was evaluated using a modified method based on the work of Dah-Nouvlessounon et al. (2023) [19]. Fifty microliters of the samples were combined with 0.2 M sodium phosphate buffer (pH 6.6) and 50 µL of 1% aqueous potassium hexacyanoferrate [K_3_Fe(CN)_6_] solution. Following a 20 min incubation at 50 °C, 50 µL of a 10% trichloroacetic acid (TCA) solution and 80 µL of each mixture were transferred to a 96-well plate containing 80 µL of distilled water and 16 µL of ferric chloride (FeCl_3_; 0.1%, *w*/*v*). Absorbance was measured using a SoftMaxR Pro 6 (version 6.3) microplate reader at a wavelength of 700 nm. All samples were tested in triplicate (n = 3).

### 2.7. α-Glucosidase Inhibition Assay

The α-glucosidase inhibition assay was conducted following the method described by Motloung et al. (2020) [20], with minor modifications. In this assay, 50 μL of various concentrations (6.25–100 μg/mL in the reaction mixture) of extracts or standard (acarbose), along with their solvent (control), were combined with 50 μL of a 2 U/mL α-glucosidase enzyme solution (dissolved in cold commercial PBS) in a transparent 96-well plate and incubated at 37 °C for 10 min. Thereafter, 50 μL of 5 mM *p*-Nitrophenyl α-D-glucopyranoside (dissolved in pre-warmed commercial PBS) was added, and the mixture was incubated for an additional 25 min at 37 °C. Thereafter, absorbance was measured at 405 nm. The enzyme inhibition (%) of the samples was calculated using the following Formula (2):

(2) $$Inhibition\;\left(\%\right)=\;\frac{Absorbance\;of\;control-Absorbance\;of\;test}{Absorbance\;of\;control}\;\times \;100\;$$

### 2.8. Molecular Docking Study

The binding affinities and interaction profiles of the compounds identified in the stem bark (sophoricoside, formononetin 7-O-glucoside, epicatechin, 6-gingerol, 6-paradol) or reference inhibitor drug (Acarbose) with the target enzyme were evaluated through molecular docking simulations. The three-dimensional (3D) structures of the ligands were retrieved from PubChem in SDF format and prepared using the Dock Prep module in UCSF Chimera (version 1.14) [21]. During preparation, all hydrogen atoms were incorporated, Gasteiger charges were assigned, and energy minimization was performed to achieve stable conformations. The crystallographic structure of α-glucosidase (GAA) (PDB ID: 5NN5) was sourced from the Protein Data Bank (PDB). The protein structure was prepared by removing water molecules and any co-crystallized ligands, followed by the addition of polar hydrogens and the assignment of appropriate charges. The active site was defined according to the coordinates of the co-crystallized ligand, and a grid box was generated to encompass the binding pocket. The grid box was centered at X = −14, Y = −31, and Z = 98, with dimensions of 40 × 32 × 31 Å along the X, Y, and Z axes, respectively. Molecular docking was performed using AutoDock (Version 1.5.6 17 September 2014) Vina [22], which employs a Lamarckian genetic algorithm to predict optimal ligand binding conformations. Each ligand was docked independently into the protein’s active site, generating multiple binding poses. The best conformations were selected based on the lowest binding energy values. To validate the docking protocol, a redocking procedure was performed in which the co-crystallized ligand was re-docked into the protein’s binding site under the same docking conditions. The accuracy of the docking method was assessed by calculating the root-mean-square deviation (RMSD) between the experimentally observed and predicted ligand poses. An RMSD value of <2.0 Å was deemed indicative of a reliable docking protocol. In addition, a reference inhibitor (positive control ligand) was included in the docking study to facilitate comparative analysis of binding affinities and interaction patterns with the tested compounds. The binding interactions of the best-ranked docking poses were further analyzed using BIOVIA Discovery Studio Visualizer, which generated two-dimensional (2D) interaction diagrams to identify key interactions, including hydrogen bonds, hydrophobic contacts, and π–π stacking, within the active site.

### 2.9. Cytotoxicity Testing

#### 2.9.1. Cell Culture

African green monkey (Vero) kidney cells (ATCC CCL-81, RRID: CVCL_0059; Cellonex, South Africa) were cultured in minimal essential medium containing Earle’s balanced salt solution and 2.0 mM L-glutamine (Cytiva HyClone, Marlborough, MA, USA), supplemented with 10% fetal bovine serum and 1% penicillin or streptomycin (Biowest, Nuaille, France). The cells were maintained at 37 °C in an incubator (Nüve, Ankara, Turkey) with an atmosphere of 95% air and 5% CO_2_. Subsequently, the cells were detached with 0.25% trypsin or ethylenediaminetetraacetic acid (EDTA) (Cytiva HyClone, Marlborough, MA, USA) and subcultured at a ratio of 1:5 upon reaching 70% to 80% confluency. Cell viability was assessed using trypan blue 0.4% (Cytiva Hyclone, Marlborough, MA, USA) on an automatic cell counter (NanoEntek, Gurogu, Seoul, South Korea), and only cell suspensions exhibiting a cell viability of ≥90% were used for the cytotoxicity assay.

#### 2.9.2. Cytotoxicity Assay

The cytotoxicity of the samples was assessed using the MTT method described by Tolosa et al. (2015) [23]. African green monkey kidney (Vero) cells were seeded at a density of 10,000 cells per well in 96-well plates and subsequently treated with a positive control (doxorubicin; Merck, Darmstadt, Germany) and various concentrations of samples. The Vero cells were allowed to adhere overnight under standard cell culture conditions. Initially, the cells were treated with different concentrations of samples (500, 250, 100, and 50 µg/mL) dissolved in dimethyl sulfoxide (DMSO) and diluted in culture medium. The positive control (doxorubicin; Merck, Darmstadt, Germany) was administered at concentrations of 10, 5, 2.5, and 1.25 µM. In each assessment, the concentration of DMSO (negative control) in the medium was ≤0.5%. Following a 48 h incubation of the 96-well plates, the culture medium containing the tested samples was removed and replaced with 200 µL of medium containing thiazolyl blue tetrazolium bromide (30 µL; 5 mg/mL) (Sigma-Aldrich, St. Louis, Missouri, USA) dissolved in PBS. After four hours incubation at 37 °C with 5% CO_2_, the culture medium was aspirated using a suction pump (Integra Biosciences Corp., Hudson, New Hampshire, USA), and 50 µL of DMSO was added to each well. The absorbance was measured using a SpectraMax iD3 multimode microplate reader (Winooski, VT, USA) at a wavelength of 570 nm. The cell viability was calculated as a percentage relative to the 0.5% DMSO negative control, as described in the following formula:

(3) $$Cell\;viability\;\left(\%\right)=\;\frac{Absorbance\;of\;sample\;-\;Absorbance\;of\;blank}{Absorbance\;of\;negative\;control\;-Absorbance\;of\;blank}\;\times \;100\;$$

### 2.10. Statistical Analysis

Data obtained for the α-glucosidase inhibition activities were analyzed in triplicate and were reported as the mean ± standard deviation. Acarbose was used as a positive control for the α-glucosidase inhibition assay. Statistical significance (*p* < 0.05) was determined using the IBM SPSS Statistics (Windows version 29.0) software when comparing the data (n = 3) across groups. One-way analysis of variance (ANOVA) and Tukey post hoc tests were used for multiple comparative analyses. The IC_50_ values for enzyme inhibition and CC_50_ values for cytotoxicity in Vero cells were calculated using a non-linear fit of the transformed (Log10) tested concentrations against the corresponding inhibitory or cytotoxic activities, utilizing GraphPad Prism 7 (Windows Version) software. For the antioxidant and cytotoxicity tests, the data were also obtained in triplicate and presented as the mean ± standard deviation. Gallic acid, ascorbic acid, and quercetin were used as reference standards for the antioxidant assay, while doxorubicin was employed as a positive control for the cytotoxicity assay. The IBM SPSS Statistics package, version 22 (Chicago, IL, USA), was used to compute statistical significance (*p* < 0.05) when comparing the data (n = 3) across samples in the antioxidant and cytotoxicity tests. The ANOVA with Duncan’s Multiple Range Test was used to evaluate differences in the measured variables among the samples in these tests.

## 3. Results

### 3.1. Chemical Fingerprint of the Crude Extracts of M. lucida Using ^1^H-NMR

All crude extracts of *M. lucida* were analyzed by ^1^H-NMR spectroscopy, with the annotation of compounds confirmed through one-dimensional (1D) ^1^H-NMR spectral analysis, comparison with the previously reported data [24,25,26,27,28,29,30,31,32,33,34], and the Human Metabolite Database (https://hmdb.ca). A total of 13 metabolites, including dicarboxylic acids, fatty acids, amino acids, disaccharides, monosaccharides, quinic acids, and flavonoids, were putatively annotated from the crude extracts (refer to Table 1). The ^1^H-NMR spectra of the extracts were categorized into three regions based on chemical shift: the aliphatic region (0–3 ppm), the sugar region (3–6 ppm), and the aromatic region (6–10 ppm), as shown in Figures S1A–S4. The chemical shifts of the annotated metabolites are presented in Table 1.

Signals corresponding to leucine and valine were identified in the dichloromethane leaf (Figure S1C,D), methanol leaf (Figure S2B,C), and stem bark extracts (Figure S3A); in contrast, betaine was only detected in the root extract (Figure S4). ^1^H-NMR signals of *α*-linoleic acid and alanine appeared in the dichloromethane leaf extract (Figure S1B,E), at δ 0.964 (t), 1.277 (m), 1.604 (m), and 5.320 (m) ppm, as well as δ 3.605 (q) ppm, respectively. Proton signals for hexadecanedioic acid were observed in the dichloromethane leaf, methanol leaf, and stem bark extracts at δ 1.262 (m), 1.277 (m), 1.464 (m), and 1.578 (m) ppm; δ 1.279 (m), 1.580 (m), and 1.263–1.280 (m) ppm; and δ 1.263–1.280 (m), and 1.578 (m) ppm, respectively (Figures S1A–S3A). Rhamnose was annotated in the methanol leaf (Figure S2D) and stem bark (Figure S3B) extracts, with signals at δ 3.844–3.852 (m), and 3.928 (m) ppm; and δ 3.841 (m), and 3.848 (m) ppm. Trehalose was detected in the dichloromethane leaf (Figure S1F), stem bark (Figure S3B), and root (Figure S4) extracts, exhibiting signals at δ 3.643 (dd), 3.847 (m), and 3.857 (m) ppm; δ 3.645 (dd), 3.841 (m), and 3.848 (m) ppm; and δ 3.834 (m), 3.845 (m), and 3.662 (m) ppm. Glucose and fructose were exclusively detected in the stem bark (Figure S3B), with signals at δ 3.869–3.923 (dd), and 3.923–3.971 (dd) ppm; and δ 3.923 (dd), and 3.971–4.033 (m) ppm, respectively. Sucrose was observed in both the stem bark (Figure S3B) and root (Figure S4) extracts at δ 3.841 (m), and 3.848 (m) ppm; and δ 3.834 (m), and 3.845 (m) ppm. The ^1^H-NMR spectra showed similarities and differences among the different crude extracts. Additionally, signals associated with chlorogenic acid were detected in the stem bark extract (Figure S3C,D), with the observed signals at δ 2.032 (m), 3.869 (dd), 3.898 (dd), 5.302 (m), 6.955 (d), and 6.940 (d) ppm. Signals corresponding to quercetin were also detected in the stem bark extract (Figure S3D), with the observed signals at δ 6.889 (d) and 6.906 (d) ppm.

### 3.2. UHPLC-Q Exactive Orbitrap MS Identification of Compounds in Morinda lucida Extracts

The untargeted screening and characterization of metabolites in *Morinda lucida* were putatively annotated by comparing their spectroscopic data with values found in published literature and databases to confirm the identity of the metabolites. Spectral data from the untargeted metabolomic analysis of *Morinda lucida* extracts were acquired using an UHPLC–Q Exactive Orbitrap MS in both positive mode (ESI (+), Figure S5, Supplementary Material) and negative mode (ESI (−), Figure S6, Supplementary Material). The negative mode (ESI (−)) was selected for further sample analysis due to its higher abundance of ions. Details on the UHPLC–Q Exactive Orbitrap MS, retention time (tR in minutes), product ion (*m*/*z*), and MS/MS spectra (*m*/*z*) are provided in Table 2. A total of 56 metabolites were putatively annotated (see Table 2), with the majority found in the leaf extracts, followed by root and stem bark extracts. Furthermore, this study represents the first comprehensive annotation and reporting of these metabolites in *Morinda lucida*.

#### 3.2.1. Flavonoids

A total of fifty-six compounds were annotated across various plant parts of *Morinda lucida*. Compound **1** (Figure S7, Supplementary Material) with tR 3.04 min, was annotated as glabranine, exhibiting a parent ion at *m*/*z* 323.26 [M − H]^−^ [MassBank-BML00154]. Peak **2** (Figure S8, Supplementary Material) displayed characteristics consistent with apigenin 6-C-glucoside 8-C-arabinoside, with a parent ion at *m*/*z* 563.43 [M − H]^−^ [35]. This peak appeared at tR 4.11 min, yielding MS/MS fragment ions at *m*/*z* 545, 520, and 503. Compounds **3** (Figure S9, Supplementary Material) and **4** (Figure S10, Supplementary Material) exhibited parent ions at *m*/*z* 449.32 and *m*/*z* 449.32, with characteristic MS/MS fragment ions at *m*/*z* 313 and 269, and *m*/*z* 447 and 316 [36,37]. Compounds **5** (gossypetin-8-C-glucoside, Figure S11, Supplementary Material), **6** (8-prenylnaringenin, Figure S12, Supplementary Material), **7** (isoschaftoside, Figure S13, Supplementary Material) and **8** (6,4′-dimethoxyisoflavone-7-glucoside, Figure S14, Supplementary Material) produced fragment ions at *m*/*z* 324,323 and 316; *m*/*z* 339; *m*/*z* 563; and *m*/*z* 296,207 [38,39,40] (MassBank-BS-BS003443). Luteolin 6-C-glucoside 8-C-arabinoside (**9**, C_23_H_24_O_10_, Figure S15, Supplementary Material), pinocembrin (**10**, C_27_H_30_O_16_, Figure S16, Supplementary Material), acacetin-7-*O*-rutinoside (**11**, C_15_H_12_O_4_, Figure S17, Supplementary Material), and 3′,4′-dimethoxy-7-hydroxyflavone (**12**, C_28_H_32_O_14_, Figure S18, Supplementary Material) exhibited molecular ions at *m*/*z* 609.41 [M − H]^−^; *m*/*z* 255.23 [M − H]^−^; *m*/*z* 591.13 [M − H]^−^; and *m*/*z* 297.24 [M − H]^−^, producing fragment ions at *m*/*z* 579, 519 and 487; *m*/*z* 217 and 211; *m*/*z* 283; and *m*/*z* 313 and 311 [35,41,42], [MassBank-BS-BS003750]. Compounds **13** (sophoricoside, Figure S19, Supplementary Material), **14** (kaempferol-7-*O*-rhamnoside, Figure S20, Supplementary Material), **15** (apigenin 8-C-glucoside, Figure S21, Supplementary Material), **16** (hyperoside, Figure S22, Supplementary Material), **17** (formononetin-7-*O*-glucoside, Figure S23, Supplementary Material) and **18** (quercetin-3-*O*-glucoside, Figure S24, Supplementary Material) exhibited molecular ions at *m*/*z* 431.23 [M − H]^−^, *m*/*z* 431.23 [M − H]^−^, *m*/*z* 431.23 [M − H]^−^, *m*/*z* 463.32 [M − H]^−^, *m*/*z* 267.07 [M − H]^−^, and *m*/*z* 464.09 [M − H]^−^, generating product ions at *m*/*z* 323, 311 and 238; *m*/*z* 283; *m*/*z* 341 and 314; *m*/*z* 455 and 453; *m*/*z* 267; and *m*/*z* 463 and 304 [43,44,45,46,47,48]. The molecular formula, C_21_H_20_O_12_, was determined for compound **19** (tR = 1.45 min), which exhibited a molecular ion peak [M − H]^−^ at *m*/*z* 464.88 (Figure S25, Supplementary Material, [49]). Compound **20** (luteone 7-glucoside) showed a molecular ion peak [M − H]^−^ at *m*/*z* 515.16, corresponding to the molecular formula C_26_H_28_O_11_. The MS/MS spectrum (Figure S26, Supplementary Material) contained a characteristic fragment at *m*/*z* 353. The annotation of this flavonoid was accomplished by comparing the spectral data with existing literature [50]. The UHPLC–Q Exactive Orbitrap MS analysis of compound **21** (tR = 1.96 min, C_16_H_12_O_3_) revealed an [M − H]^−^ ion at *m*/*z* 251.03 (Figure S27, Supplementary Material), with MS/MS fragments at *m*/*z* 209 and 207 [51]. The mass spectra of compounds **22** (Figure S28, Supplementary Material), **23** (Figure S29, Supplementary Material) and **24** (Figure S30, Supplementary Material) exhibited molecular ion peaks at *m*/*z* 595.15, 595.27 and 595.15, respectively. These were characterized as eriodictyol-7-*O* neohesperidoside (C_27_H_32_O_15_), quercetin-3-arabinoglucoside (C_26_H_28_O_16_), and quercetin-3-*O*-vicianoside (C_26_H_28_O_16_), supported by the presence of characteristic fragments at *m*/*z* 595, *m*/*z* 595, and *m*/*z* 594 and 593 [52,53,54]. Moreover, the UHPLC–Q Exactive Orbitrap MS spectra of compounds **25** (Figure S31, Supplementary Material) and **26** (Figure S32, Supplementary Material) revealed [M − H]^−^ ions at *m*/*z* 447.09 and 289.18, respectively. These compounds were identified as quercitrin and epicatechin, with characteristic daughter fragments at *m*/*z* 455 and 303, and *m*/*z* 259 and 243 [53,55].

#### 3.2.2. Terpenoids

In the UHPLC-Q Exactive Orbitrap MS analysis utilizing the negative electrospray ionization mode, eight terpenoids were annotated in *Morinda lucida* crude extracts. Compound **27** (Figure S33, Supplementary Material) was assigned as 3-*O*-acetyl-16alpha-hydroxydehydrotrametenolic acid. It revealed its [M − H]^−^ ion at *m*/*z* 511.33, which generated a fragment ion at *m*/*z* 511 [56]. Furthermore, corosolic acid (**28**), sumaresinolic acid (**29**), and maslinic acid (**30**) exhibited characteristic fragmentations at *m*/*z* 426 and 418 (Figure S34, Supplementary Material), *m*/*z* 463 and 437 (Figure S35, Supplementary Material), and *m*/*z* 238 (Figure S36, Supplementary Material). These compounds were previously reported by Liu et al. (2022) [57] and Sánchez-González et al. (2013) [58], respectively. Meanwhile, poricoic acid A (**31**), alpha-boswellic acid (**32**), poricoic acid B (**33**) and medicagenic acid (**34**) exhibited distinct fragment ions at *m*/*z* 405, 377 and 216 (Figure S37, Supplementary Material); *m*/*z* 454 and 453 (Figure S38, Supplementary Material); *m*/*z* 483 (Figure S39, Supplementary Material); and *m*/*z* 485 and 421 (Figure S40, Supplementary Material), respectively [59,60,61,62].

#### 3.2.3. Phenolic Acids and Phenolic Compounds

Compounds **35** (Figure S41, Supplementary Material) and **36** (Figure S42, Supplementary Material), were eluted at 6.20 min and 2.58 min, with the molecular ions [M − H]^−^ at *m*/*z* 442.07 (C_22_H_18_O_10_) and 295.23 (C_16_H_25_NO_4_), respectively; these were annotated as catechin gallate and esmolol, respectively, by comparing the retention times and MS/MS information with data from the literature [MassBank-BS-BS003891,55]. Compound **37** (Figure S43, Supplementary Material), detected at 1.46 min, with the precursor ion at [M − H]^−^ at *m*/*z* 293.18 and fragment ions at *m*/*z* 248, 237 and 209, was annotated as 6-gingerol [55]. Compounds **38** (Figure S44, Supplementary Material) and **39** (Figure S45, Supplementary Material), with molecular ions of *m*/*z* 277.20 [M − H]^−^ and *m*/*z* 305.92 [M − H]^−^ were found at 2.33 and 1.51 min, respectively. These compounds yielded the fragment ions at *m*/*z* 283, 233, and 205; *m*/*z* 289 and 221, and were annotated as 6-paradol and (−)-epigallocatechin in comparison with data from the literature [49,55].

#### 3.2.4. Fatty Acids

The molecular ion peaks observed at *m*/*z* 339.94 [M − H]^−^ and *m*/*z* 311.22 [M − H]^−,^ along with their corresponding fragment ion signals at *m*/*z* 337 and 319, and *m*/*z* 293, were annotated as behenic acid (**40**, Figure S46, Supplementary Material) and arachidic acid (**41**, Figure S47, Supplementary Material). This identification was achieved through comparative analysis with the retention times (tR) and mass spectral data published in the literature [63]. Eicosadienoic acid (**42**) and stearic acid (**43**) displayed mass spectral peaks at *m*/*z* 307.19 [M − H]^−^ and *m*/*z* 283.03 [M − H]^−^, respectively. UHPLC–Q Exactive Orbitrap MS revealed chemical product fragment ions at *m*/*z* 290 and 289 (Figure S48, Supplementary Material) and *m*/*z* 283 (Figure S49, Supplementary Material). These secondary metabolites were previously reported by Servi et al. (2022) [64] and MSBNK-Antwerp_Univ-METOX_N109326_B8BB, respectively.

#### 3.2.5. Alkaloids and Others

Fragmentation analyses of yohimbic acid (**44**, *m*/*z* 321,297 [M − H]^−^; C_20_H_24_N_2_O_3_) and isomajdine (**45**, *m*/*z* 397,381,285 [M − H]^−^; C_23_H_28_N_2_O_6_) yielded precursor ions at *m*/*z* 339.12 (Figure S50, Supplementary Material; [65]) and *m*/*z* 427.18 (Figure S51, Supplementary Material; [66]). Compounds **46** (Figure S52, Supplementary Material) and **47** (Figure S53, Supplementary Material) were putatively annotated as delphinidin 3-galactoside (*m*/*z* 464.09 [M − H]^−^, [C_21_H_21_O_12_]^+^) and delphinidin-3-*O*-sambubioside (*m*/*z* 596.16 [M − H]^−^, [C_26_H_29_O_16_]^+^), respectively [67,68]. Additionally, molecular ions at *m*/*z* 675.30 [M − H]^−^ and *m*/*z* 974.90 [M − H]^−^ detected at retention times 2.78 min and 10.43 min were annotated as hederagenin base + *O*-AcetylHex (**48**, Figure S54, Supplementary Material, [69]) and (**49**, Figure S55, Supplementary Material, MassBank-RIKEN-PR308930). The annotation of compounds **50** (Figure S56, Supplementary Material), **51** (Figure S57, Supplementary Material) and **52** (Figure S58, Supplementary Material), detected at 1.91 min, 1.46 min and 1.52 min, respectively, revealed precursor ions at *m*/*z* 423.17 [M − H]^−^, *m*/*z* 533.38 [M − H]^−^ and *m*/*z* 339.07 [M − H]^−^ [55,70,71]. Furthermore, compounds **53** (tR = 1.31 min, C_14_H_8_O_5_), **54** (tR = 1.14 min, C_20_H_20_O_6_) and **55** (tR = 8.45 min, C_24_H_34_O_4_) exhibited precursor ions at *m*/*z* 255.23, *m*/*z* 355.16, *m*/*z* 386.88, which yielded characteristic fragment ions at *m*/*z* 226; *m*/*z* 353, 265 and 226; and *m*/*z* 431, respectively. These compounds were putatively annotated as purpurin (Figure S59, Supplementary Material, [72]), licoagrodione (Figure S60, Supplementary Material, [73]), and bufalin (Figure S61, Supplementary Material, [74]). In contrast, compound **56** (chlorogenic acid, C_16_H_18_O_9_), with a precursor ion at *m*/*z* 353.11, yielded the characteristic fragments ion at *m*/*z* 309, 295, and 283 (Figure S62, Supplementary Material, [50]).

### 3.3. Molecular Networking of Morinda lucida Metabolites

**Figure 1:**
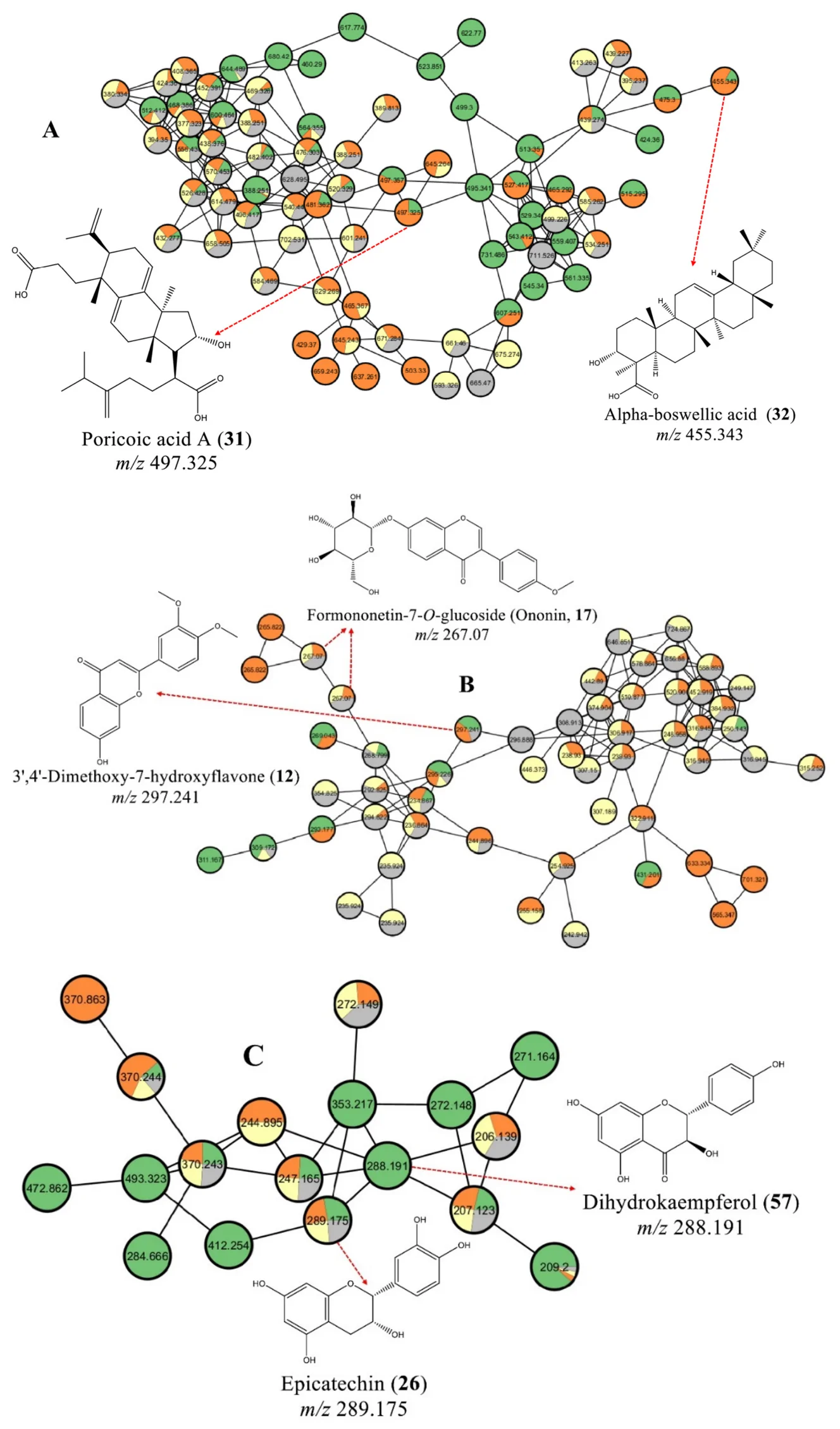

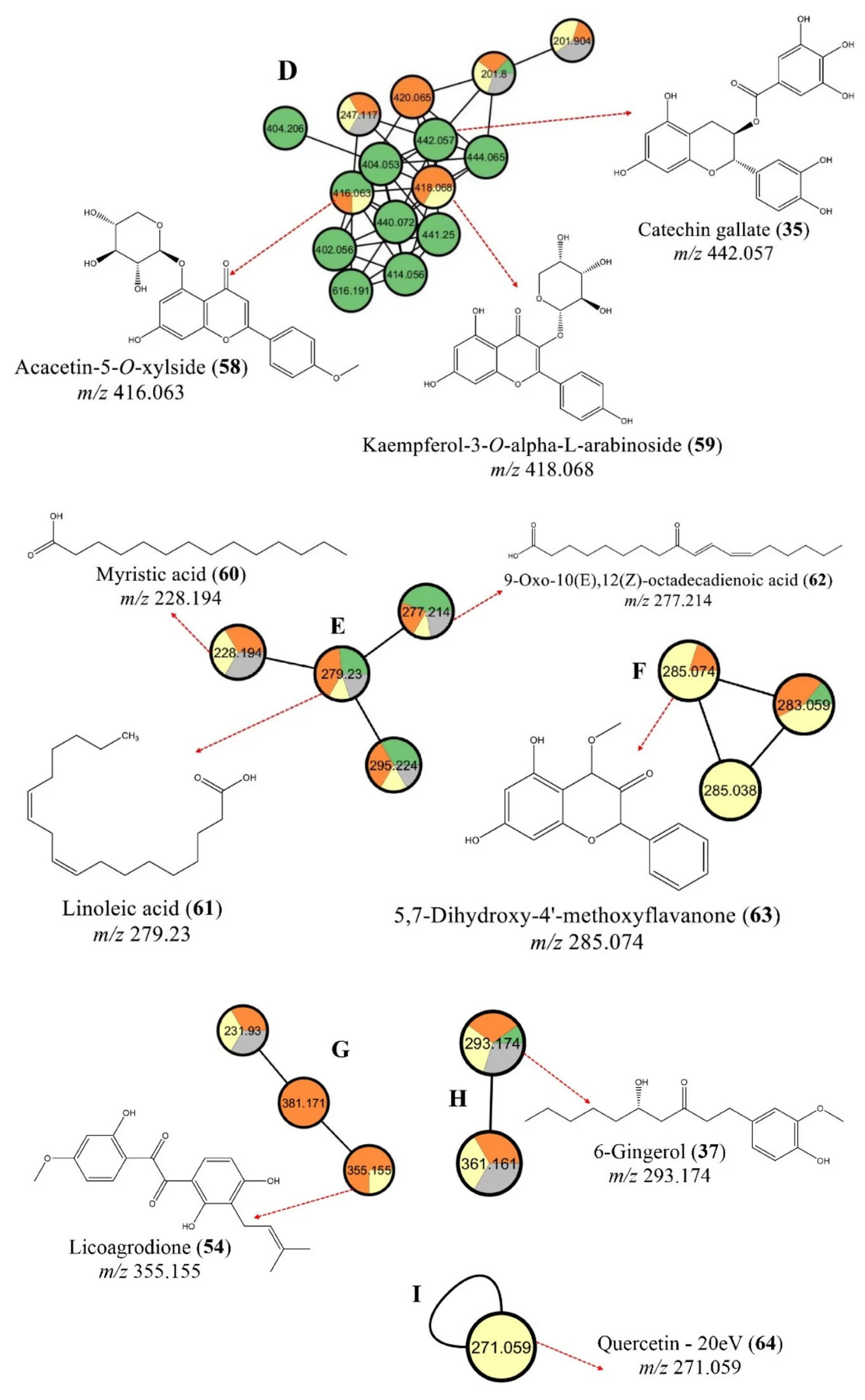
Molecular network of *Morinda lucida* crude extracts. Nodes of cluster (**A**) (lipids and lipid-like molecules), cluster (**B**) (phenylpropanoids and polyketides), cluster (**C**) (phenylpropanoids and polyketides), cluster (**D**) (phenylpropanoids and polyketides), cluster (**E**) (lipids and lipid-like molecules), cluster (**F**) (phenylpropanoids and polyketides), cluster (**G**) (phenylpropanoids and polyketides), cluster (**H**) (benzenoids), and cluster (**I**) (phenylpropanoids and polyketides) were labeled with the precursor mass. Nodes represent annotated compounds and are colored according to the respective crude extracts: dichloromethane leaf crude extract—orange nodes, methanol leaf crude extract—green nodes, root crude extract—gray nodes, and stem bark crude extract—yellow nodes.

To explore the chemical profiles of *Morinda* species, various extracts from different plant parts of *Morinda lucida*, including leaves, root, and stem bark, were analyzed using UHPLC–Q Exactive Orbitrap MS. This analysis was completed by molecular networking in negative ESI mode via the Global Natural Products Social Networking (GNPS) platform (http://gnps.ucsd.edu, accessed on 7 July 2025). The UHPLC–Q Exactive Orbitrap MS chromatograms of both ESI (−/+); Figures S5 and S6) of the *M. lucida* crude extracts are included in the Supplementary Materials. A detailed molecular network was constructed based on converted MS^2^ data, which reflected the similarity in metabolite fragmentation patterns. The resulting molecular networks (Clusters **A**–**I**) were visualized in Cytoscape version 3.9.1 [17], as shown in Figure 1. A total of 850 molecular ions exhibited MS^2^ spectra across four crude extracts, represented by nodes interconnected by 1258 edges within the molecular network.

### 3.4. Antioxidant Activity of M. lucida Extracts

As shown in Table 3, the root extract exhibited significant radical scavenging activity, exhibiting an IC_50_ value of 7.7550 ± 6.9142 µg/mL, while the methanol leaf extract showed the lowest radical scavenging activity, with an IC_50_ = 48.2661 ± 7.1855 µg/mL. The reducing power activity ranged from 0.0052 ± 0.0025 to 0.0487 ± 0.0212 µg/mL (IC_0.5_), in the following descending order: root extract > dichloromethane leaf extract > stem bark > methanol leaf extract (Table 3). Furthermore, the root extract exhibited high reducing power activity with an IC_0.5_ value of 0.0052 ± 0.0025 µg/mL, whereas the methanol leaf extract displayed the lowest reducing power activity (IC_0.5_ = 0.4146 ± 0.1429 µg/mL), as indicated in Table 3.

### 3.5. Inhibition of α-Glucosidase Activity of M. lucida Extracts and Computational Docking

**Figure 2:**
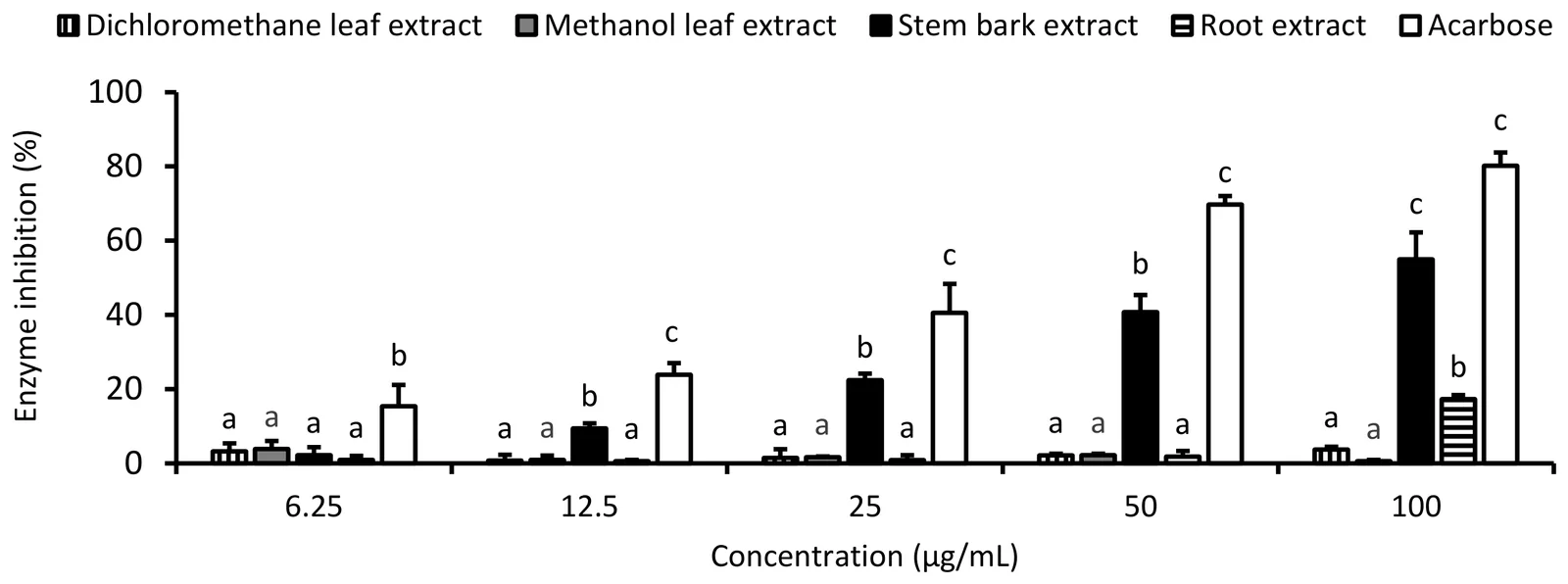
Dose-dependent α-glucosidase-inhibitory action of *Morinda lucida* extracts and Acarbose (standard drug). Data are presented as mean ± standard deviation (SD) of triplicate experiments. For each concentration, statistical multiple comparisons (Tukey and one-way ANOVA; IBM SPSS) were performed between the different treatment groups. The letters at the top of the error bars of the different treatments represent a significant difference (*p* < 0.05) when there are no letters that are common to the compared groups.

**Figure 3:**
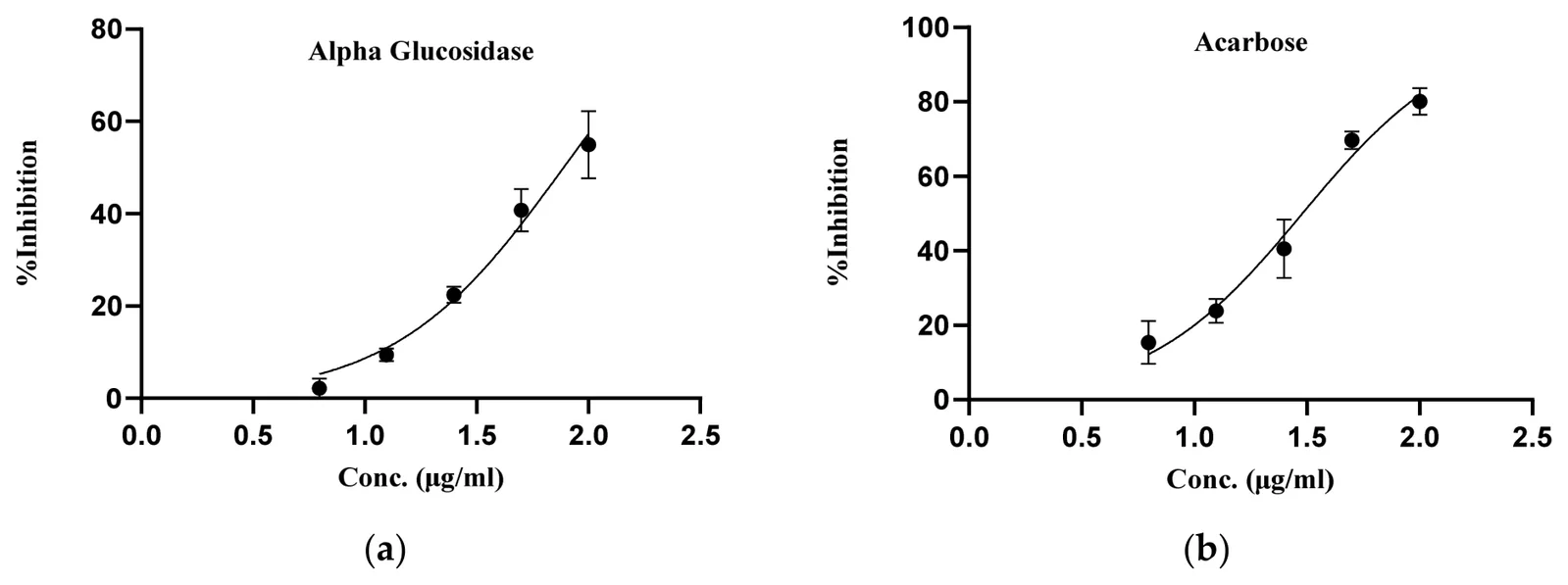
IC_50_ inhibition curves for the α-glucosidase inhibition by (**a**) stem bark extract and (**b**) Acarbose.

**Figure 4:**
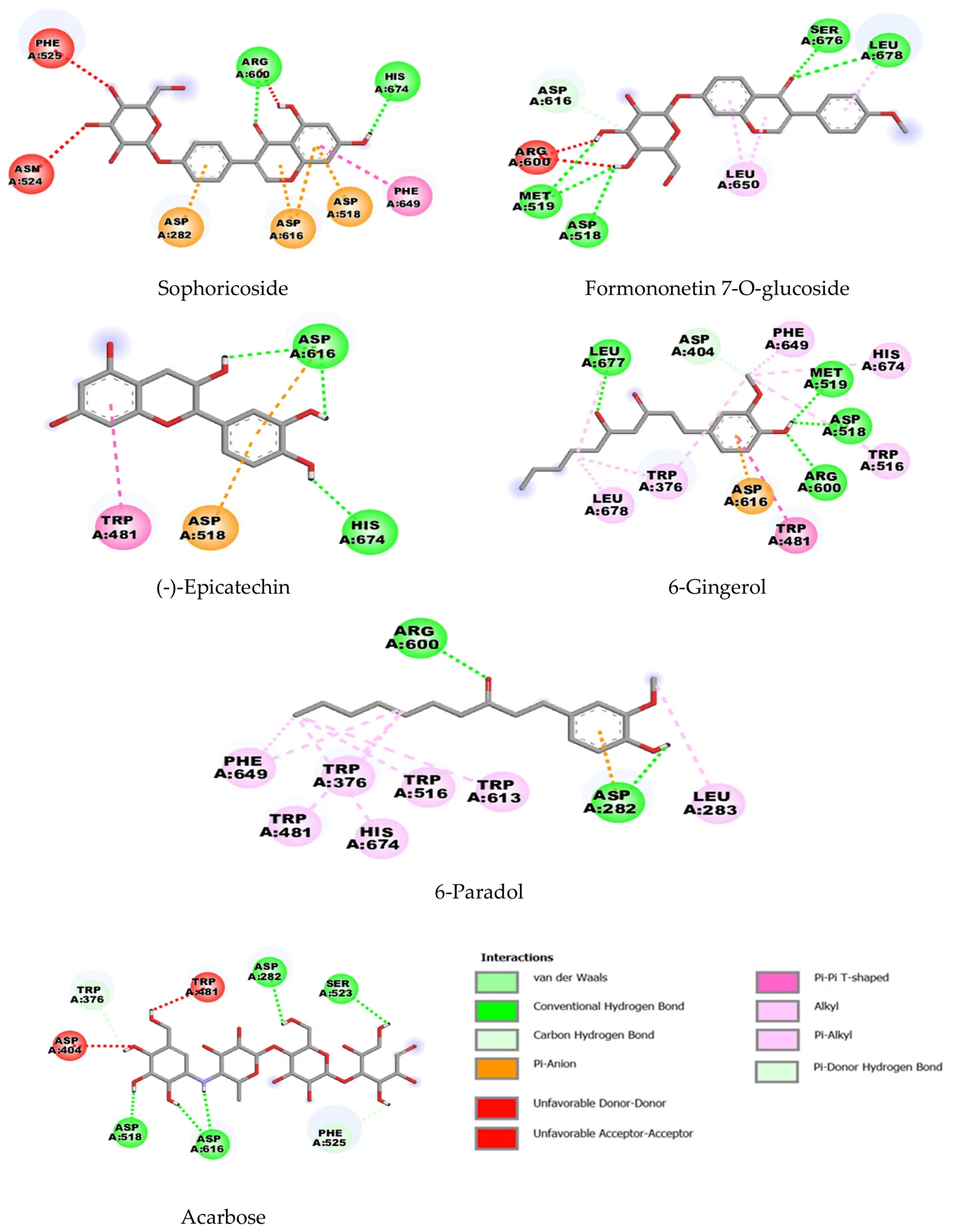
Images showing the interactions between identified compounds and α-glucosidase protein.

As shown in Figure 2 and Figure 3 and Table 4, the stem bark extract exhibited appreciable α-glucosidase inhibitory activity (IC_50_ = 79.9 ± 16.1 µg/mL), whereas the other extracts showed no notable α-glucosidase inhibition. However, the inhibitory activity of the stem bark extract was significantly lower (*p* ˂ 0.05) than that of acarbose (IC_50_ = 30.1 ± 4.00 µg/mL). In addition, flavonoid glycosides sophoricoside and formononetin 7-O-glucoside, together with epicatechin identified in the stem bark extract, showed notable in silico interactions with the α-glucosidase protein (Estimated Binding Energy = −7.5, −7.8 and −7.6 kcal/mol, respectively), which was comparable to that of acarbose (Estimated Binding Energy = −6.5 kcal/mol) Table 5 and Figure 4. The 3D visualization of the molecular docking analysis against 5NN5 for the isolated compounds are presented in Figure S63 of the Supplementary Material.

### 3.6. Cytotoxicity of the Crude Extracts of M. lucida

**Figure 5:**
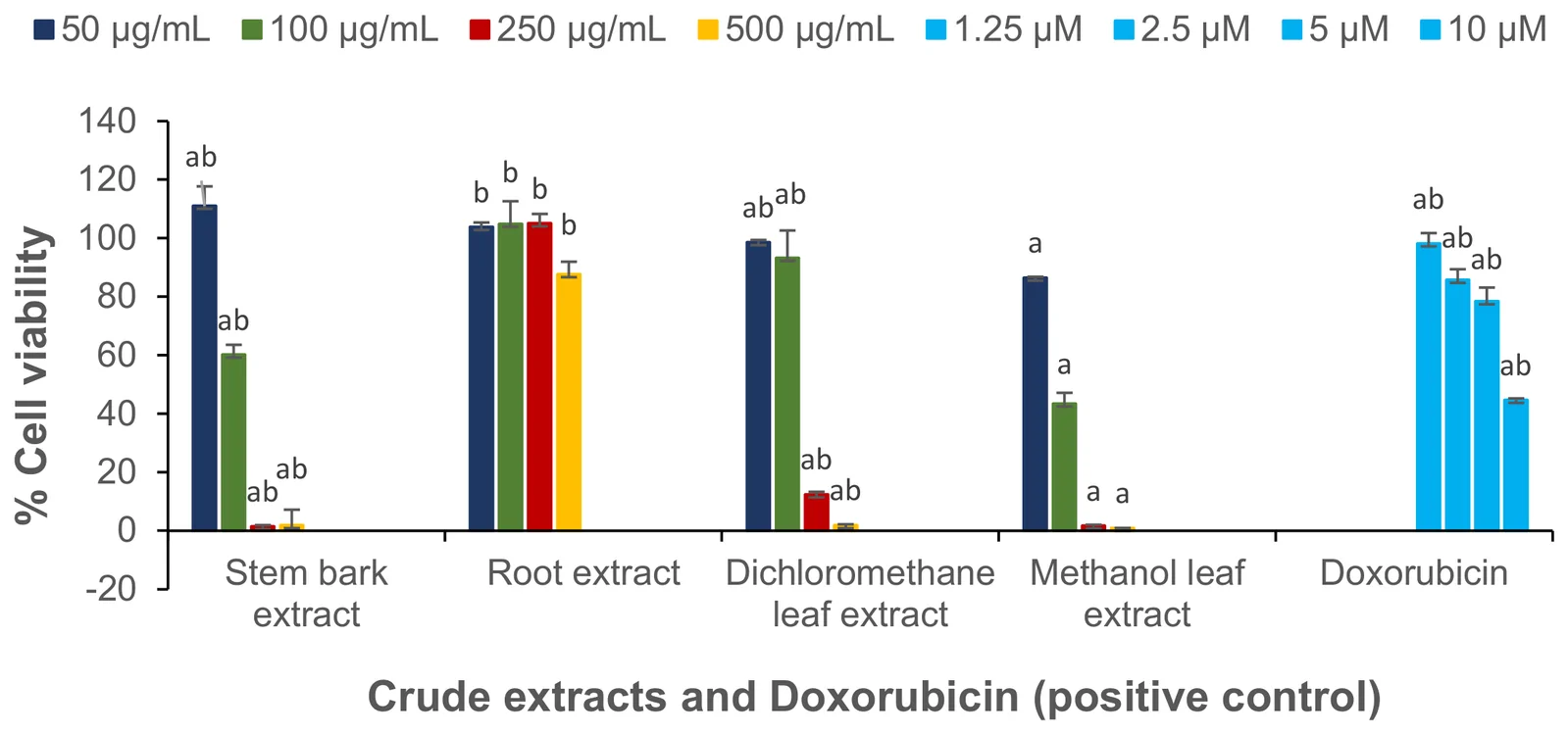
Cytotoxicity of *Morinda lucida* extracts against Vero cells at different concentrations of 50, 100, 250, and 500 μg/mL. Mean differences between extracts and doxorubicin were evaluated using one-way ANOVA with DMRT. Different letters indicate significant differences among extracts; the difference with *p* < 0.05 was considered statistically significant. Error bars indicate the SD of triplicate (n = 3) values obtained from a single experiment. All tests were performed in triplicate (n = 3), and the data are presented as the mean ± SD of two independent experiments. Data for the positive control (doxorubicin) at different concentrations (1.25, 2.5, 5, and 10) are shown for comparison.

The cytotoxicity of *M. lucida* crude extracts was evaluated using the MTT assay against African green monkey (Vero) kidney cells (Figure 5). The cytotoxic concentration (CC_50_ in μg/mL, Table 6) was determined from the percentage of cell viability relative to that of the positive control (doxorubicin, Table 6 and Figure 5). As shown in Figure 5, at 50 μg/mL onward, no toxicity was observed in the root extract. In contrast, the stem bark and leaf extracts exhibited cytotoxicity at concentrations of 250 and 500 μg/mL (Figure 5).

## 4. Discussion

The study explored the metabolomic profile, antioxidant, α-glucosidase inhibitory, and cytotoxic activities of compounds from different plant parts of *Morinda lucida*. A wide range of metabolites were putatively annotated from the leaf, root, and stem bark extracts of *M. lucida* using ^1^H-NMR, UHPLC–Q Exactive Orbitrap MS, and molecular networking (refer to Table 1 and Table 2 and Figure 1). Most of these metabolites were annotated in the leaf extracts. Metabolomic profiling indicated that different *Morinda lucida* plant organs produced both similar and distinct chemical compounds.

The combination of GNPS-based molecular networking and manual confirmation facilitated the annotation of unique secondary metabolites from various plant parts of *M. lucida*. Compounds **57**–**64** were annotated through GNPS, whereas compounds **12**, **17**, **26**, **31**, **32**, **35**, **37**, and **54** were manually identified. Clusters A and B (Figure 1) were characterized by precursor ions with *m*/*z* values of 497.325 and 455.343; as well as 297.241 and 267.07, which were annotated as poricoic acid A (**31**) and alpha-boswellic acid (**32**), and 3′,4′-dimethoxy-7-hydroxyflavone (**11**) and formononetin-7-*O*-glucoside (Ononin, **16**), respectively. In cluster C, the precursor ions at *m*/*z* 289.175 and *m*/*z* 288.191 were designated as epicatechin (**26**) and dihydrokaempferol (**57**). Cluster D included precursor ions with *m*/*z* values of 416.063, 418.068, and 442.057, which were annotated as acacetin-5-*O*-xyloside (**58**), kaempferol-3-*O*-alpha-L-arabinoside (**59**), and catechin gallate (**35**), respectively. Myristic acid (**60**), with a precursor ion at *m*/*z* 228.194, was identified in the dichloromethane extracts of leaves, roots, and stem bark (see cluster E, Figure 1). Additionally, linoleic acid (**61**) and 9-oxo-10(E),12(Z)-octadecadienoic acid (**62**), with precursor ions at *m*/*z* 279.23 and 277.214, were detected in all crude extracts (see cluster E, Figure 1). Compounds **63** (*m*/*z* 285.074 [M − H]^−^) and **54** (*m*/*z* 355.155 [M − H]^−^), annotated as 5,7-dihydroxy-4′-methoxyflavanone and licoagrodione, were exclusively found in the dichloromethane leaf and stem bark extracts (as shown in clusters F and G, Figure 1). In contrast, cluster H contained the metabolite at *m*/*z* 293.174, annotated as 6-gingerol (**37**), which was only present in the dichloromethane leaf, methanol leaf, stem bark, and root extracts. Cluster I included the precursor ion with *m*/*z* 271.059 [M − H]^−^, which was annotated as quercetin −20 eV (**64**).

The results from radical scavenging and reducing power assays showed that the presence of various compounds, including glabranine (**1**), apigenin 6-C-glucoside 8-C-arabinoside (**2**), isookanin-7-*O*-glucoside (**3**), myricetin-3-*O*-xyloside (**4**), isoschaftoside (**7**), 6,4′-limethoxyisoflavone-7-glucoside (Wistin, **8**), luteolin 6-C-glucoside 8-C-arabinoside (**9**), pinocembrin (**10**), acacetin-7-*O*-rutinoside (Linarin, **11**), 3′,4′-dimethoxy-7-hydroxyflavone (**12**), kaempferol-7-*O*-rhamnoside (**14**), apigenin 8-C-glucoside (Vitexin, **15**), hyperoside (**16**), formononetin-7-*O*-glucoside (Ononin, **17**), quercetin-3-*O*-glucoside (Isoquercitrin, **18**), myricitrin (**19**), 7-hydroxy-3-methylflavone (**21**), eriodictyol-7-*O*-neohesperidoside (Neoeriocitrin, **22**), quercetin-3-arabinoglucoside (Peltatoside, **23**), quercetin-3-*O*-vicianoside (**24**), quercitrin (**25**), epicatechin (**26**), corosolic acid (**28**), medicagenic acid (**34**), 6-gingerol (**37**), (−)-epigallocatechin (**39**), behenic acid (**40**), eicosadienoic acid (**42**), stearic acid (**43**), arachidic acid (**41**), chlorogenic acid (**56**), delphinidin-3-*O*-sambubioside (**47**), and 5,7-dihydroxy-4′-methoxyflavanone (**63**) might have contributed to the antioxidant activities of the crude extracts. These phytochemicals have been previously documented for their antioxidant properties [75,76,77,78,79,80,81,82,83,84,85,86,87,88,89,90,91,92,93,94,95,96,97,98,99,100,101,102,103,104,105,106,107,108]. A study conducted by Nigussie et al. (2023) and Nwozo et al. (2023) indicated that the antioxidant capacities of numerous medicinal plant crude extracts are attributed to the high quality of phenolic compounds, flavonoids, and terpenoids [109,110]. The presence of hydroxyl groups (-OH) in phenolic compounds reacts with reactive nitrogen species (RNS) and oxygen species in a termination reaction, thereby inhibiting the formation of new radicals [111]. Kubiak-Tomaszewska et al. (2022) noted that esterification or blocking of hydroxyl groups (-OH) in the flavonoid structures diminishes their ability to scavenge free radicals or chelate metals [112]. Conversely, Zhou et al. (2022) reported that the varying numbers of unsaturated carbon–carbon double bonds (C=C) in terpenoids contribute to the strong antioxidant capacities of these secondary metabolites [113]. The findings of this study reveal that the IC_50_ and IC_0.5_ values of the different crude extracts of *M. lucida* were significantly lower than those of gallic acid, quercetin, and ascorbic acid, as determined through DPPH (2,2-diphenyl-1-picrylhydrazin) scavenging and reducing power assays. This suggests that *M. lucida* may serve as a valuable antioxidant in the treatment of free radicals.

Moreover, the results from the α-glucosidase inhibitory assay revealed that the presence of metabolites, including epicatechin (**26**), formononetin-7-*O*-glucoside (Ononin, **17**), and quercetin −20 eV (**64**) may have contributed to the α-glucosidase inhibitory activity of the stem bark extract. This is consistent with several studies reporting the anti-diabetic activity of these compounds through α-glucosidase inhibition [114,115,116,117]. Olaokun and Zubair (2023) reported that epicatechin (**26**), isolated from the ethyl acetate fraction of *Ficus lutea* leaf extract by silica gel column chromatography, exhibited potent α-glucosidase inhibitory activity (IC_50_ = 5.72 ± 2.7 µg/mL, [114]). Similarly, Zhang et al. (2022) reported that epicatechin (**26**), tentatively identified in the ethanol extract of *Senegalia catechu*, contributed to the inhibition of α-glucosidase activity of this plant species [115]. Dilshad et al. (2023) demonstrated that formononetin-7-*O*-glucoside (Ononin, **17**), identified by reverse-phase ultra-high-performance liquid chromatography–MS (RP-UHPLC–MS), contributed to the inhibition of α-glucosidase activity of the methanolic extract of *Typha domingensis* [116]. Furthermore, Bouslamti et al. (2023) reported that quercetin −20 eV (**64**), detected by high-performance liquid chromatography with a diode array detector (HPLC-DAD), contributed to the α-glucosidase inhibitory activity of the hydroethanolic leaf, fruit, and flower extracts of *Solanum elaeagnifolium* Cav [117]. Molecular docking results support the possible contribution of sophoricoside, formononetin 7-O-glucoside, and epicatechin to the α-glucosidase inhibitory activity of the stem bark.

The results of the cytotoxicity test demonstrated that the stem bark and leaf extracts were more toxic to African green monkey (Vero) kidney cells than the root extract at different concentrations (Figure 5). This might be due to the presence of isoschaftoside (**7**), formononetin-7-*O*-glucoside (Ononin, **17**), myricitrin (**19**), quercetin-3-*O*-vicianoside (**24**), quercitrin (**25**), corosolic acid (**28**), maslinic acid (**30**), medicagenic acid (**34**), behenic acid (**40**), eicosadienoic acid (**42**), isomajdine (**45**), delphinidin-3-*O*-sambubioside (**47**), bufalin (**55**), and chlorogenic acid (**56**), which were detected in the stem bark and root extracts. This is consistent with several studies reporting the cytotoxicity of these natural compounds [90,100,104,118,119,120,121,122,123,124]. According to Ahmadi et al. (2021) and Rasool et al. (2023), no natural products or crude plant extracts should be regarded as safe until they are evaluated for cellular toxicity [125,126]. Furthermore, it is important to assess the cytotoxicity of natural products using more than one cell line, as conclusions regarding the efficacy and safety of a natural product based on a single cell line may be misleading.

## 5. Conclusions

^1^H-NMR- and UHPLC–Q Exactive Orbitrap MS-based metabolomics combined with molecular networking and molecular docking analysis were used to annotate metabolites associated with the antioxidant, α-glucosidase inhibitory, and cytotoxic activities of different plant parts of *M. lucida*. A total of 77 metabolites were annotated using the ^1^H-NMR, UHPLC–Q Exactive Orbitrap MS, and molecular networking approaches. Phytochemical profiling revealed both similarities and differences in the compounds produced by different plant parts of *M. lucida*. Additionally, the findings indicated that root extracts exhibited significantly higher antioxidant activities compared with leaf and stem bark extracts. The study also demonstrated that stem bark extracts provided notable inhibition of α-glucosidase, potentially attributed to the identified flavonoid glycosides (sophoricoside and formononetin 7-O-glucoside) and epicatechin. Furthermore, the in silico interactions between these compounds and the α-glucosidase protein were stronger than those of acarbose. The findings of this study indicate that flavonoids, flavonoid glycosides, terpenoids, phenolic compounds, polyphenols, saturated fatty acids, quinic acids, anthocyanins, alkaloids, and bufadienolides contribute to antioxidant and α-glucosidase activities, as well as the cytotoxicity of *M. lucida*. These findings provide strong scientific support for the use of analytical techniques such as ^1^H-NMR and UHPLC–Q Exactive Orbitrap MS to expedite drug discovery and development. Further research is recommended to isolate and validate the annotated phytochemicals, investigate phytochemicals in various *Morinda* species, and assess their bioactivities through in vitro, in vivo, and in silico experiments to identify and develop potential drug candidates.

## Figures and Tables

**Table 1:** Characteristic signals of the putatively annotated metabolites in the ^1^H-NMR spectra of *Morinda lucida* extracts: s = singlet; d = doublet; dd = doublet of doublets; m = multiplet; q = quartet.

Compounds No.	Metabolites	Chemical Shifts (δ ppm) and Multiplicity (Experiment)	Chemical Shifts (δ ppm) and Multiplicity (Literature)	Human Metabolome Database	Crude Extracts	References
**1**	Hexadecanedioic acid	1.262 (m), 1.277 (m), 1.263–1.280 (m), 1.279 (m), 1.464 (m), 1.578 (m), 1.580 (m), 1.649–1.657 (m)	1.26 (m), 1.57 (m)	1.25 (m), 1.27 (m), 1.56 (m), 1.64 (m), 1.65 (m)	Dichloromethane leaf, Methanol leaf, Stem bark	[24]
**2**	*α*-Linoleic acid	0.964 (t), 1.277 (m), 1.604 (m), 5.320 (m)	0.96 (t), 1.27 (m), 5.32 (m)	1.61 (m), 5.33 (m)	Dichloromethane leaf	[25]
**3**	Leucine	0.947 (dd), 0.978 (dd), 0.989 (dd), 0.996 (dd), 0.967–0.987 (m), 1.009 (dd), 2.321 (m)	0.94 (dd), 1.00 (dd)	0.99 (dd), 1.00 (dd), 2.34 (dd)	Dichloromethane leaf, Methanol leaf, Stem bark	[26]
**4**	Valine	0.904 (m), 0.906 (m), 0.956 (m), 0.964 (m), 0.965 (m) 0.996 (d), 1.030 (d), 2.030 (m), 2.034 (m), 2.037 (m)	0.95 (m), 1.02 (d)	0.90 (m), 0.95 (m), 1.02 (d), 2.04 (m),	Dichloromethane leaf, Methanol leaf, Stem bark	[27]
**5**	Betaine	3.304 (s)	3.278 (s)	3.32 (s)	Root	[28]
**6**	Alanine	3.605 (q)	3.60(q)	3.60 (q)	Dichloromethane leaf	[29]
**7**	Rhamnose	3.841 (m), 3.848 (m)	3.84 (m), 3.85 (m),	3.84 (m), 3.85 (m),	Methanol leaf, Stem bark	[30]
**8**	Sucrose	3.841 (m), 3.848 (m), 3.834 (m), 3.845 (m)	3.83 (m), 3.86 (m)	3.83 (m), 3.86 (m)	Stem bark, Root	[31]
**9**	Trehalose	3.643 (dd), 3.645 (dd), 3.834 (m), 3.841 (m), 3.845 (m), 3.847 (m), 3.848 (m), 3.857 (m), 3.662 (m)	3.64 (dd), 3.83 (m), 3.85 (m), 3.6 (m)	3.65 (dd), 3.84 (m), 3.85 (m)	Dichloromethane leaf, Stem bark, Root	[32]
**10**	Fructose	3.923 (dd), 3.971–4.033 (m)	3.93–3.98 (dd), 3.98–4.08 (m)	3.95–3.99 (m), 3.99–4.10 (m)	Stem bark	[32]
**11**	Glucose	3.869–3.923 (dd), 3.923–3.971 (dd)	3.86–3.92 (dd)	3.86–3.93 (dd), 3.93–3.98 (dd)	Stem bark	[32]
**12**	Chlorogenic acid	2.032 (m), 3.869 (dd), 3.898 (dd), 5.302 (m), 6.955 (d), 6.940 (d)	2.03 (m), 3.88 (dd), 6.95 (d), 6.96 (d)	2.02 (m), 3.86 (dd), 3.89 (dd), 5.36 (m), 6.93 (d), 6.95 (d)	Stem bark	[33]
**13**	Quercetin	6.889 (d), 6.906 (d)	6.88 (d), 6.90 (d)	6.89 (d), 6.90 (d)	Stem bark	[34]

**Table 2:** Putative annotation of compounds from crude extracts of *M. lucida* by UHPLC-Q Exactive Orbitrap MS in the negative ion mode (DCM leaf—dichloromethane leaf extract; MeOH leaf—methanol leaf extract; stem bark—stem bark extract; root—root extract; X—compound absent in the extract; and √—compound present in the extract).

Compound No.	tR (min)	Theoretical Mass [M − H]^−^ (*m*/*z*)	Observed Mass [M − H]^−^ (*m*/*z*)	Molecular Formula	MS^2^ Fragment Ions (*m*/*z*)	Compound Name	Compound Class	DCM Leaf	MeOH Leaf	Root	Stem Bark	References
**1**	3.04	323.12	323.26	C_20_H_20_O_4_	323	Glabranine	Flavonoid	√	X	X	X	MassBank-BML00154
**2**	4.11	563.13	563.43	C_26_H_28_O_14_	545,520,503	Apigenin 6-C-glucoside 8-C-arabinoside	Flavonoid	√	X	X	X	[35]
**3**	3.34	449.11	449.32	C_21_H_22_O_11_	313,269	isookanin-7-*O*-glucoside	Flavonoid	√	X	X	X	[36]
**4**	1.50	449.07	449.32	C_20_H_18_O_12_	447,316	Myricetin-3-*O*-xyloside	Flavonoid	√	X	X	X	[37]
**5**	3.13	479.08	479.28	C_21_H_20_O_13_	324,323,316	Gossypetin-8-C-glucoside	Flavonoid glycoside	√	X	X	X	[38]
**6**	1.52	339.12	339.20	C_20_H_20_O_5_	339	8-Prenylnaringenin	Prenylated flavonoid	√	X	X	X	[39]
**7**	2.35	563.14	563.01	C_26_H_28_O_14_	563	Isoschaftoside	Flavonoid	√	X	X	X	[40]
**8**	8.89	459.13	459.76	C_23_H_24_O_10_	296,207	6,4′-Dimethoxyisoflavone-7-glucoside (Wistin)	Flavonoid	√	X	X	X	MassBank- BS-BS003443
**9**	2.41	609.14	609.41	C_27_H_30_O_16_	579,519,487	Luteolin 6-C-glucoside 8-C-arabinoside	Flavonoid	√	X	X	X	[35]
**10**	1.31	255.06	255.23	C_15_H_12_O_4_	217,211	Pinocembrin	Flavonoid	√	X	X	X	[41]
**11**	1.79	591.13	591.13	C_28_H_32_O_14_	283	Acacetin-7-*O*-rutinoside (Linarin)	Flavone glycoside	√	X	X	X	[42]
**12**	2.96	297.24	297.24	C_17_H_14_O_5_	313,311	3′,4′-Dimethoxy-7-hydroxyflavone	Flavonoid	√	√	√	X	MassBank-BS-BS003750
**13**	0.35	431.20	431.23	C_21_H_20_O_10_	323,311,238	Sophoricoside	Isoflavone glycoside	√	X	√	√	[43]
**14**	0.40	431.10	431.23	C_21_H_20_O_10_	283	Kaempferol-7-*O*-rhamnoside	Flavonoid	√	X	X	X	[44]
**15**	0.40	431.09	431.23	C_21_H_20_O_10_	341,314	Apigenin 8-C-glucoside (Vitexin)	Flavonoid	√	√	X	X	[45]
**16**	7.46	463.05	463.32	C_21_H_20_O_12_	455,453	Hyperoside	Flavonoid	√	√	X	X	[46]
**17**	2.12	267.02	267.07	C_22_H_22_O_9_	267	Formononetin-7-*O*-glucoside (Ononin)	Isoflavone glycoside	√	X	√	√	[47]
**18**	2.06	464.38	464.09	C_21_H_20_O_12_	463,304	Quercetin-3-*O*-glucoside (Isoquercitrin)	Flavonoid	√	X	X	X	[48]
**19**	1.45	464.09	464.88	C_21_H_20_O_12_	464	Myricitrin	Flavonoid	√	√	X	X	[49]
**20**	1.49	515.16	515.16	C_26_H_28_O_11_	353	Luteone 7-glucoside	Flavonoid	X	√	X	X	[50]
**21**	1.96	251.07	251.03	C_16_H_12_O_3_	209,207	7-Hydroxy-3-Methylflavone	Flavonoid	X	X	√	X	[51]
**22**	1.68	595.16	595.15	C_27_H_32_O_15_	595	Eriodictyol-7-*O*-neohesperidoside (Neoeriocitrin)	Flavonoid	X	X	√	X	[52]
**23**	0.48	595.12	595.27	C_26_H_28_O_16_	595	Quercetin-3-arabinoglucoside (Peltatoside)	Flavonoid	X	X	√	X	[53]
**24**	1.68	595.13	595.15	C_26_H_28_O_16_	594,593	Quercetin-3-*O*-vicianoside	Flavonoid	√	X	√	X	[54]
**25**	1.97	447.09	447.09	C_21_H_20_O_11_	455,303	Quercitrin	Flavonoid	√	X	X	X	[46]
**26**	0.60	289.10	289.18	C_15_H_14_O_6_	259,243	Epicatechin	Flavonoid	√	√	√	√	[55]
**27**	2.54	511.33	511.33	C_32_H_48_O_5_	511	3-*O*-Acetyl-16alpha-hydroxydehydrotrametenolic acid	Triterpenoid	√	X	X	√	[56]
**28**	1.45	471.42	471.38	C_30_H_48_O_4_	426,418	Corosolic acid	Pentacyclic triterpenoid	√	X	X	X	[57]
**29**	2.06	471.34	471.07	C_30_H_48_O_4_	463,437	Sumaresinolic acid	Pentacyclic triterpenoid	√	X	X	X	[57]
**30**	3.34	471.34	471.34	C_30_H_48_O_4_	238	Maslinic acid	Pentacyclic triterpenoid	√	√	X	X	[58]
**31**	4.86	497.32	497.31	C_31_H_46_O_5_	405,377,216	Poricoic acid A	Tricyclic triterpenoid	√	√	X	X	[59]
**32**	1.45	455.35	455.30	C_30_H_48_O_3_	454,453	Alpha-boswellic acid	Pentacyclic terpenoid	√	√	X	X	[60]
**33**	1.50	483.31	483.12	C_30_H_44_O_5_	483	Poricoic acid B	Triterpenoid	X	X	√	X	[61]
**34**	1.55	501.32	501.11	C_30_H_46_O_6_	485,421	Medicagenic acid	Triterpenoid	X	X	X	√	[62]
**35**	6.20	442.09	442.07	C_22_H_18_O_10_	440,313,293	Catechin gallate	Polyphenol	X	√	X	X	MassBank-BS-BS003891
**36**	2.58	295.17	295.23	C_16_H_25_NO_4_	-	Esmolol	Phenolic compound	√	√	√	√	[55]
**37**	1.46	293.17	293.18	C_17_H_26_O_4_	248,237,209	6-Gingerol	Phenolic compound	√	√	√	√	[55]
**38**	2.33	277.18	277.20	C_17_H_26_O_3_	283,233,205	6-Paradol	Phenolic ketone	√	√	√	√	[55]
**39**	1.51	305.06	305.92	C_15_H_14_O_7_	289,221	(−)-Epigallocatechin	Polyphenol	√	X	X	X	[49]
**40**	1.55	339.32	339.94	C_22_H_44_O_2_	337,319	Behenic acid	Saturated fatty acid	√	X	X	X	[63]
**41**	1.16	311.29	311.22	C_20_H_40_O_2_	293	Arachidic acid	Saturated fatty acid	X	X	√	X	[63]
**42**	1.83	307.26	307.19	C_20_H_36_O_2_	290,289	Eicosadienoic acid	Polyunsaturated fatty acid	√	√	X	X	[64]
**43**	2.33	283.26	283.03	C_18_H_36_O_2_	283	Stearic acid	Saturated fatty acid	√	X	X	X	MSBNK-Antwerp_Univ-METOX_N109326_B8BB
**44**	0.23	339.17	339.12	C_20_H_24_N_2_O_3_	321,297	Yohimbic acid	Monoterpene indole alkaloids	√	X	X	X	[65]
**45**	0.65	427.18	427.18	C_23_H_28_N_2_O_6_	397,381,285	Isomajdine	Indole Alkaloid	√	X	X	X	[66]
**46**	2.06	464.08	464.09	[C_21_H_21_O_12_]^+^	463,337	Delphinidin 3-galactoside	Anthocyanin	√	√	X	X	[67]
**47**	1.76	596.17	596.16	[C_26_H_29_O_16_]^+^	595,593	Delphinidin-3-*O*-sambubioside	Anthocyanin	√	X	√	X	[68]
**48**	2.78	675.411	675.30	C_38_H_60_O_10_	635,619	Hederagenin base + *O*-AcetylHex	Saponin	X	√	X	X	[69]
**49**	10.43	974.55	974.90	C_48_H_78_O_20_	718,645,511	Bayogenin base + *O*-Hex, *O*-Hex-Hex	Saponin	X	√	X	X	MassBank-RIKEN-PR308930
**50**	1.91	423.09	423.17	C_19_H_20_O_11_	313	3-Glucosyl-2,3′,4,4′,6- pentahydroxybenzophenone	Glycoside	√	X	X	X	[55]
**51**	1.46	533.12	533.38	C_24_H_22_O_13_	533	6ʹʹ -*O*-Malonylgenistin	Glycosyloxy isoflavone	√	X	X	X	[70]
**52**	1.52	339.06	339.07	C_15_H_16_O_9_	338	6,7-Dihydroxycoumarin-6-glucoside (Esculin)	Coumarin glucoside	√	X	X	X	[71]
**53**	1.31	255.03	255.23	C_14_H_8_O_5_	226	Purpurin	Anthraquinone	√	X	X	X	[72]
**54**	1.14	355.14	355.16	C_20_H_20_O_6_	353,265,226	Licoagrodione	Stilbenoid	√	X	X	√	[73]
**55**	8.45	386.53	386.88	C_24_H_34_O_4_	431	Bufalin	Bufadienolide	√	√	X	X	[74]
**56**	1.49	353.50	353.11	C_16_H_18_O_9_	309,295,283	Chlorogenic acid	Quinic acid	X	√	X	X	[50]

**Table 3:** Antioxidant activity of *Morinda lucida* extracts.

Samples	DPPH IC_50_ (µg/mL)	Reducing Power IC_0.5_ (µg/mL)
Dichloromethane leaf extract	12.2042 ± 5.0489 ^ab^	0.0487 ± 0.0212 ^a^
Methanol leaf extract	48.2661 ± 7.1855 ^d^	0.4146 ± 0.1429 ^a^
Root extract	7.7550 ± 6.9142 ^a^	0.0052 ± 0.0025 ^a^
Stem bark extract	18.7235 ± 18.3297 ^abc^	0.1979 ± 0.0361 ^a^
Gallic acid	24.8936 ± 24.8680 ^abcd^	5.4730 ± 9.2647 ^a^
Quercetin	37.3613 ± 21.3565 ^bcd^	3.7606 ± 2.9793 ^a^
Ascorbic acid	44.3083 ± 9.8132 ^cd^	0.2366 ± 0.3453 ^a^

Notes: Values are expressed as mean ± standard deviation of three replicates (n = 3). Different letters indicate significant differences among extracts using one-way analysis of variance (ANOVA); Duncan’s Multiple Range Test (DMRT); at *p* < 0.05.

**Table 4:** IC_50_ values for the α-glucosidase inhibition of extracts and Acarbose.

	Dichloromethane Leaf Extract	Methanol Leaf Extract	Stem Bark Extract	Root Extract	Acarbose
IC_50_ values (µg/mL)	NA-ND	NA-ND	79.9 ± 16.1 ^a^	NA-ND	30.1 ± 4.00 ^b^

“NA-ND” means “no activity-not determined”. Data are shown as mean ± SD of a triplicate experiment. For each concentration, statistical multiple comparisons (Tukey and one-way ANOVA; IBM SPSS) were performed between the different treatment groups. The superscript letters next to the IC_50_ values of the different treatments represent a significant difference (*p* < 0.05) when there are no letters that are common to the compared groups.

**Table 5:** Molecular docking scores.

Proteins	Compounds	Estimated Binding Energy (kcal/mol)	Hydrogen Bonds (Distance Å)	Electrostatic Interactions	Hydrophobic Interaction
α-glucosidase (GAA–5NN5)	Sophoricoside	−7.5	ARG600 (2.34577) HIS674 (2.42431)	ASP282 ASP518 ASP616 ASP616	PHE649
	Formononetin 7-O-glucoside (Ononin)	−7.8	SER676 (2.9883) LEU678 (3.081) ASP518 (2.97188) MET519 (2.75112) MET519 (2.98221) ASP616 (3.03683)		LEU650 (x2) LEU678
	(-)-Epicatechin	−7.6	ASP616 (2.52276) HIS674 (2.27707) ASP616 (2.75691)	ASP518 ASP616	TRP481
	6-Gingerol	−6.1	ARG600 (2.80342) LEU677 (3.05526) ASP518 (1.95766) MET519 (3.07399) ASP404 (3.39197)	TRP481	LEU677 LEU678 TRP376 (x2) TRP516 PHE649 HIS674
	6-Paradol	−6.1	ARG600 (2.95729) ASP282 (2.03181)	ASP282	LEU283 TRP376 TRP481 TRP516 TRP613 PHE649 (x2) HIS674
	Acarbose	−6.5	ASP616 (2.46815) ASP616 (2.85218) ASP518 (1.80743) ASP282 (2.56368) SER523 (2.3205) TRP376 (3.27691) PHE525 (2.82991)		

**Table 6:** Inhibitory concentrations (CC_50_ in µg/mL) of *Morinda lucida* extracts and doxorubicin (positive control) in Vero monkey kidney cell lines.

Samples	Vero IC_50_ (µg/mL)
Dichloromethane leaf extract	169.00 ± 1.61 ^d^
Methanol leaf extract	91.30 ± 1.75 ^b^
Root extract	> 500
Stem bark extract	101.80 ± 4.69 ^c^
Doxorubicin (positive control)	9.16 ± 1.54 ^a^

Notes: The values above are presented as mean ± SD of three replicates (n = 3). Different letters indicate significant differences among extracts using one-way ANOVA; DMRT; at *p* < 0.05.

## Data Availability

Data are contained within the article and Supplementary Materials.

## Supplementary Materials

The following supporting information can be downloaded at https://www.mdpi.com/article/10.3390/metabo16080523/s1, Figure S1: (A) The representative ^1^H-NMR spectra of the dichloromethane leaf extract. 1, hexadecanedioic acid; (B) The representative ^1^H-NMR spectra of the dichloromethane leaf extract. 2, *α*-linoleic acid; (C) The representative ^1^H-NMR spectra of the dichloromethane leaf extract. 3, leucine; (D) The representative ^1^H-NMR spectra of the dichloromethane leaf extract. 4, valine; (E) The representative ^1^H-NMR spectra of the dichloromethane leaf extract. 6, alanine; (F) The representative ^1^H-NMR spectra of the dichloromethane leaf extract. 9, trehalose; Figure S2: (A) The representative ^1^H-NMR spectra of the methanol leaf extract. 1, hexadecanedioic acid; (B) The representative ^1^H-NMR spectra of the methanol leaf extract. 3, leucine; (C) The representative ^1^H-NMR spectra of the methanol leaf extract. 4, valine; (D) The representative ^1^H-NMR spectra of the methanol leaf extract. 7, rhamnose; Figure S3: (A) The representative ^1^H-NMR spectra of the stem bark extract. 1, hexadecanedioic acid; 3, leucine; 4, valine; (B) The representative ^1^H-NMR spectra of the stem bark extract. 7, rhamnose; 8, sucrose; 9, trehalose; 10, fructose; 11, glucose; (C) The representative ^1^H-NMR spectra of the stem bark extract. 12, chlorogenic acid; (D) The representative ^1^H-NMR spectra of the stem bark extract. 12, chlorogenic acid; 13, quercetin; Figure S4: The representative ^1^H-NMR spectra of the root extract. 5, betaine; 8, sucrose; 9, trehalose; Figure S5: UHPLC–Q/Orbitrap/MS HRMS chromatograms with relative abundance and retention time (min) of (**1**) dichloromethane leaf; (**2**) methanol leaf; (**3**); stem bark and (**4**) root extracts of *Morinda lucida* obtained in positive mode electrospray ionization; Figure S6: UHPLC–Q/Orbitrap/MS HRMS chromatograms with relative abundance and retention time (min) of (**1**) dichloromethane leaf; (**2**) methanol leaf; (**3**) stem bark; and (**4**) root extracts of *Morinda lucida* obtained in negative mode electrospray ionization; Figure S7: UHPLC–Q/Orbitrap/MS HRMS spectrum showing fragments of glabranine (**1**); Figure S8: UHPLC–Q/Orbitrap/MS HRMS spectrum showing fragments of apigenin 6-C-glucoside 8-C-arabinoside (**2**); Figure S9: UHPLC–Q/Orbitrap/MS HRMS spectrum showing fragments of isookanin-7-*O*-glucoside (**3**); Figure S10: UHPLC–Q/Orbitrap/MS HRMS spectrum showing fragments of myricetin-3-*O*-xyloside (**4**); Figure S11: UHPLC–Q/Orbitrap/MS HRMS spectrum showing fragments of gossypetin-8-C-glucoside (**5**); Figure S12: UHPLC–Q/Orbitrap/MS HRMS spectrum showing fragments of 8-prenylnaringenin (**6**); Figure S13: UHPLC–Q/Orbitrap/MS HRMS spectrum showing fragments of isoschaftoside (**7**); Figure S14: UHPLC–Q/Orbitrap/MS HRMS spectrum showing fragments of 6,4′-dimethoxyisoflavone-7-glucoside (Wistin) (**8**); Figure S15: UHPLC–Q/Orbitrap/MS HRMS spectrum showing fragments of luteolin 6-C-glucoside 8-C-arabinoside (**9**); Figure S16: UHPLC–Q/Orbitrap/MS HRMS spectrum showing fragments of pinocembrin (**10**); Figure S17: UHPLC–Q/Orbitrap/MS HRMS spectrum showing fragments of acacetin-7-*O*-rutinoside (Linarin) (**11**); Figure S18: UHPLC–Q/Orbitrap/MS HRMS spectrum showing fragments of 3′,4′-dimethoxy-7-hydroxyflavone (**12**); Figure S19: UHPLC–Q/Orbitrap/MS HRMS spectrum showing fragments of sophoricoside (**13**); Figure S20: UHPLC–Q/Orbitrap/MS HRMS spectrum showing fragments of kaempferol-7-*O*-rhamnoside (**14**); Figure S21: UHPLC–Q/Orbitrap/MS HRMS spectrum showing fragments of apigenin 8-C-glucoside (Vitexin) (**15**); Figure S22: UHPLC–Q/Orbitrap/MS HRMS spectrum showing fragments of hyperoside (**16**); Figure S23: UHPLC–Q/Orbitrap/MS HRMS spectrum showing fragments of formononetin-7-*O*-glucoside (Ononin) (**17**); Figure S24: UHPLC–Q/Orbitrap/MS HRMS spectrum showing fragments of quercetin-3-*O*-glucoside (Isoquercitrin) (**18**); Figure S25: UHPLC–Q/Orbitrap/MS HRMS spectrum showing fragments of myricitrin (**19**); Figure S26: UHPLC–Q/Orbitrap/MS HRMS spectrum showing fragments of luteone 7-glucoside (**20**); Figure S27: UHPLC–Q/Orbitrap/MS HRMS spectrum showing fragments of 7-hydroxy-3-methylflavone (**21**); Figure S28: UHPLC–Q/Orbitrap/MS HRMS spectrum showing fragments of eriodictyol-7-*O*-neohesperidoside (Neoeriocitrin) (**22**); Figure S29: UHPLC–Q/Orbitrap/MS HRMS spectrum showing fragments of quercetin-3-arabinoglucoside (Peltatoside) (**23**); Figure S30: UHPLC–Q/Orbitrap/MS HRMS spectrum showing fragments of quercetin-3-*O*-vicianoside (**24**); Figure S31: UHPLC–Q/Orbitrap/MS HRMS spectrum showing fragments of quercitrin (**25**); Figure S32: UHPLC–Q/Orbitrap/MS HRMS spectrum showing fragments of epicatechin (**26**); Figure S33: UHPLC–Q/Orbitrap/MS HRMS spectrum showing fragments of 3-*O*-acetyl-16alpha-hydroxydehydrotrametenolic acid (**27**); Figure S34: UHPLC–Q/Orbitrap/MS HRMS spectrum showing fragments of corosolic acid (**28**); Figure S35: UHPLC–Q/Orbitrap/MS HRMS spectrum showing fragments of sumaresinolic acid (**29**); Figure S36: UHPLC–Q/Orbitrap/MS HRMS spectrum showing fragments of maslinic acid (**30**); Figure S37: UHPLC–Q/Orbitrap/MS HRMS spectrum showing fragments of poricoic acid A (**31**); Figure S38: UHPLC–Q/Orbitrap/MS HRMS spectrum showing fragments of alpha-boswellic acid (**32**); Figure S39: UHPLC–Q/Orbitrap/MS HRMS spectrum showing fragments of poricoic acid B (**33**); Figure S40: UHPLC–Q/Orbitrap/MS HRMS spectrum showing fragments of medicagenic acid (**34**); Figure S41: UHPLC–Q/Orbitrap/MS HRMS spectrum showing fragments of catechin gallate (**35**); Figure S42: UHPLC–Q/Orbitrap/MS HRMS spectrum showing fragments of esmolol (**36**); Figure S43: UHPLC–Q/Orbitrap/MS HRMS spectrum showing fragments of 6-gingerol (**37**); Figure S44: UHPLC–Q/Orbitrap/MS HRMS spectrum showing fragments of 6-paradol (**38**); Figure S45: UHPLC–Q/Orbitrap/MS HRMS spectrum showing fragments of (−)-epigallocatechin (**39**); Figure S46: UHPLC–Q/Orbitrap/MS HRMS spectrum showing fragments of behenic acid (**40**); Figure S47: UHPLC–Q/Orbitrap/MS HRMS spectrum showing fragments of arachidic acid (**41**); Figure S48: UHPLC–Q/Orbitrap/MS HRMS spectrum showing fragments of eicosadienoic acid (**42**); Figure S49: UHPLC–Q/Orbitrap/MS HRMS spectrum showing fragments of stearic acid (**43**); Figure S50: UHPLC–Q/Orbitrap/MS HRMS spectrum showing fragments of yohimbic acid (**44**); Figure S51: UHPLC–Q/Orbitrap/MS HRMS spectrum showing fragments of isomajdine (**45**); Figure S52: UHPLC–Q/Orbitrap/MS HRMS spectrum showing fragments of delphinidin 3-galactoside (**46**); Figure S53: UHPLC–Q/Orbitrap/MS HRMS spectrum showing fragments of delphinidin-3-*O*-sambubioside (**47**); Figure S54: UHPLC–Q/Orbitrap/MS HRMS spectrum showing fragments of hederagenin base + *O*-AcetylHex (**48**); Figure S55: UHPLC–Q/Orbitrap/MS HRMS spectrum showing fragments of bayogenin base + *O*-Hex, *O*-Hex-Hex (**49**); Figure S56: UHPLC–Q/Orbitrap/MS HRMS spectrum showing fragments of 3-glucosyl-2,3′,4,4′,6- pentahydroxybenzophenone (**50**); Figure S57: UHPLC–Q/Orbitrap/MS HRMS spectrum showing fragments of 6ʹʹ -*O*-malonylgenistin (**51**); Figure S58: UHPLC–Q/Orbitrap/MS HRMS spectrum showing fragments of 6,7-dihydroxycoumarin-6-glucoside (Esculin) (**52**); Figure S59: UHPLC–Q/Orbitrap/MS HRMS spectrum showing fragments of purpurin (**53**); Figure S60: UHPLC–Q/Orbitrap/MS HRMS spectrum showing fragments of licoagrodione (**54**); Figure S61: UHPLC–Q/Orbitrap/MS HRMS spectrum showing fragments of bufalin (**55**); Figure S62: UHPLC–Q/Orbitrap/MS HRMS spectrum showing fragments of chlorogenic acid (**56**); Figure S63: 3D visualization for molecular docking against 5NN5 for the isolated compounds.
