# Supplementary material for: Comprehensive Profiling of Antioxidant, Antidiabetic, and Cytotoxic Compounds from Morinda lucida Benth Using 1H-NMR- and UHPLC-Q Exactive Orbitrap MS-Based Metabolomics Combined with Molecular Networking and Molecular Docking
Source: Metabolites. 2026 Jul 24;16(8):523. doi: 10.3390/metabo16080523 (PMC13515774; doi:10.3390/metabo16080523)
Supplement: Supplementary file 1 [file metabolites-16-00523-s001.zip › metabolites-4348013-supplementary.pdf]

metabolites

Supplementary Materials

Comprehensive Profiling of Antioxidant, Antidiabetic and Cytotoxic Compounds from *Morinda lucida* Benth Using ^{1}H-NMR- and UHPLC-Q Exactive-Orbitrap-MS-Based Metabolomics Combined with Molecular Networking and Molecular Docking

Dorcas Tlhapi [1,*], Ntsoaki Malebo [2], Idah Tichaidza Manduna [1], Monizi Mawunu [3,4], Chika Ifeanyi Chukwuma [5], Ramakwala Christinah Chokwe [6] and Kolawole Olofinsan [7]

[1] Centre for Applied Food Sustainability and Biotechnology, Faculty of Health and Environmental Sciences, Central University of Technology, Bloemfontein 9301, South Africa; imanduna@cut.ac.za
[2] Department of Life Sciences, Faculty of Health and Environmental Sciences, Central University of Technology, Bloemfontein, 9301, South Africa; nmalebo@cut.ac.za
[3] Department of Agronomy, Polytechnic Institute, Kimpa Vita University, Uíge P.O. Box 77, Angola; m.mawunu2000@gmail.com
[4] Department of Biology, Faculty of Science and Technology, University of Kinshasa, Kinshasa P.O. Box 190, Congo
[5] Centre for Quality of Health and Living, Faculty of Health and Environmental Sciences, Central University of Technology, Bloemfontein 9301, South Africa; cchukwuma@cut.ac.za
[6] Department of Chemistry, College of Science Engineering and Technology, University of South Africa, Florida, Johannesburg 1710, South Africa; chokwrc@unisa.ac.za
[7] Department of Pharmacology, Faculty of Health Sciences, University of the Free State, Bloemfontein 9300, South Africa; olofinsan.k@ufs.ac.za
* Correspondence: btlhapi@cut.ac.za

Network analysis job link

1. Molecular network ESI(-):ID= 16f6e39554c4470ea69b2f2ea1f5572d

https://gnps.ucsd.edu/ProteoSAFe/status.jsp?task= 16f6e39554c4470ea69b2f2ea1f5572d

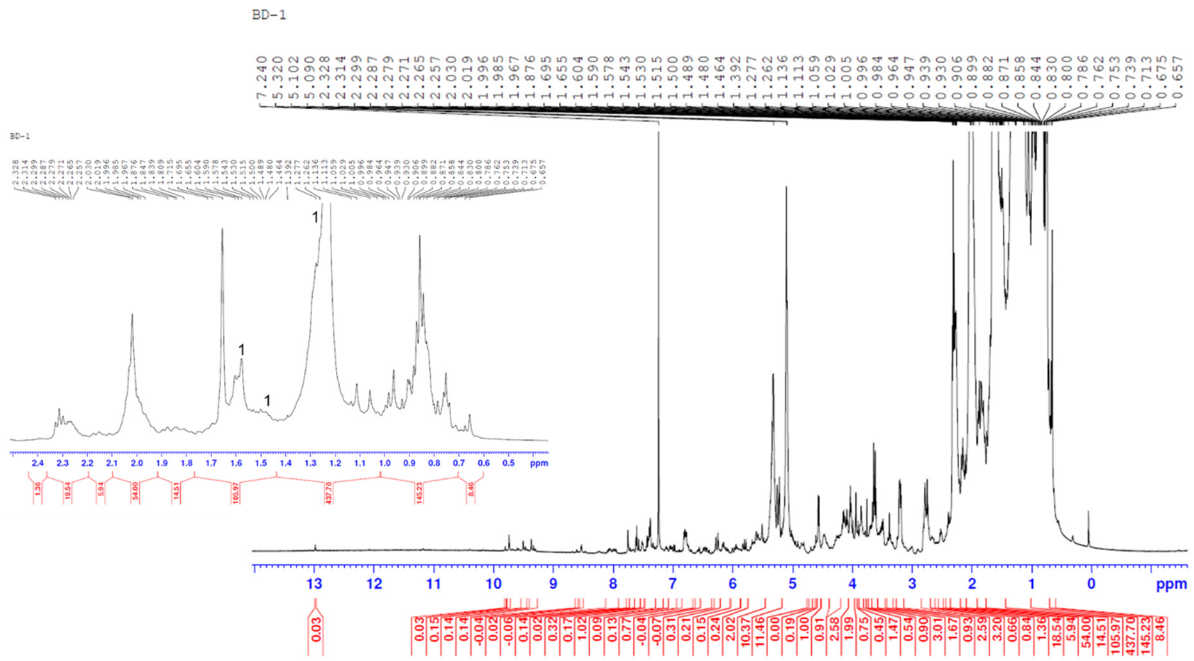

Figure S1. (A) The representative ^{1}H-NMR spectra of the dichloromethane leaf extract. 1, hexadecanedioic acid.

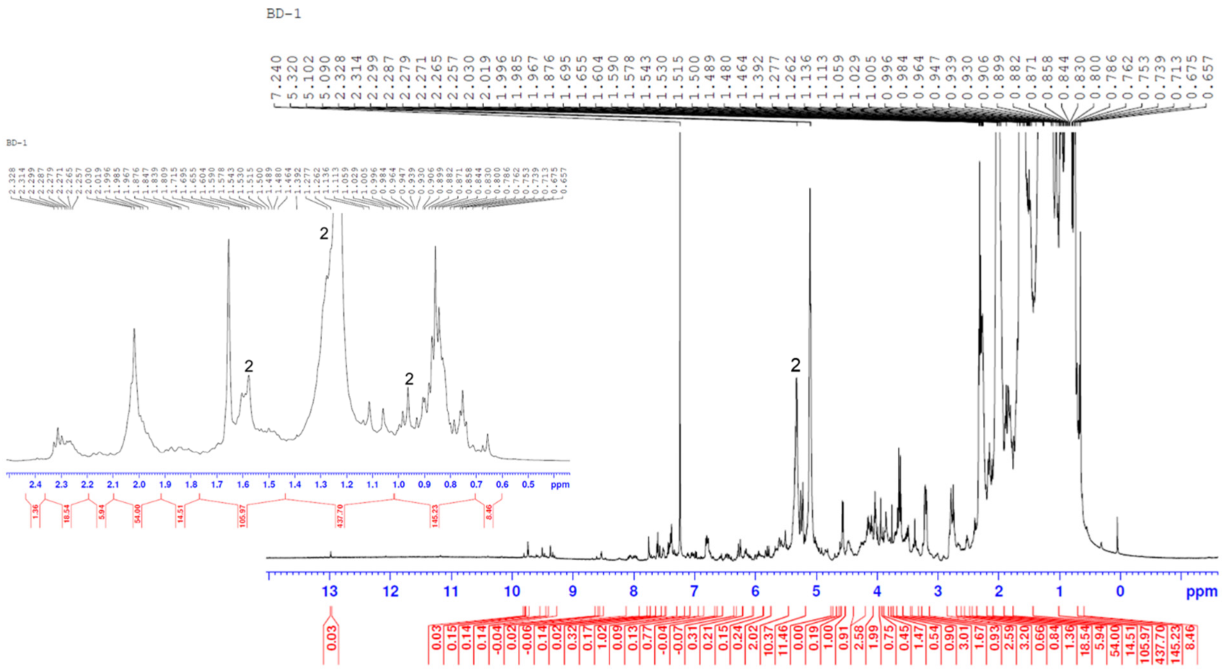

(B) The representative ^{1}H-NMR spectra of the dichloromethane leaf extract. 2, α-linoleic acid.

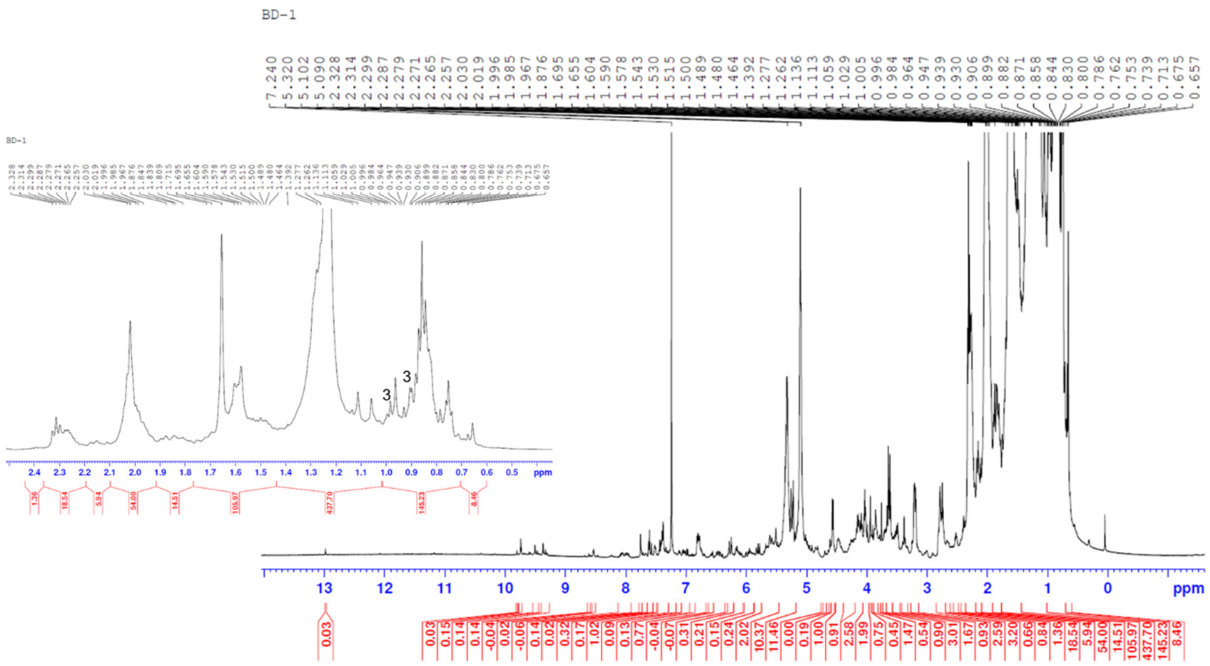

(C) The representative ¹H-NMR spectra of the dichloromethane leaf extract. 3, leucine.

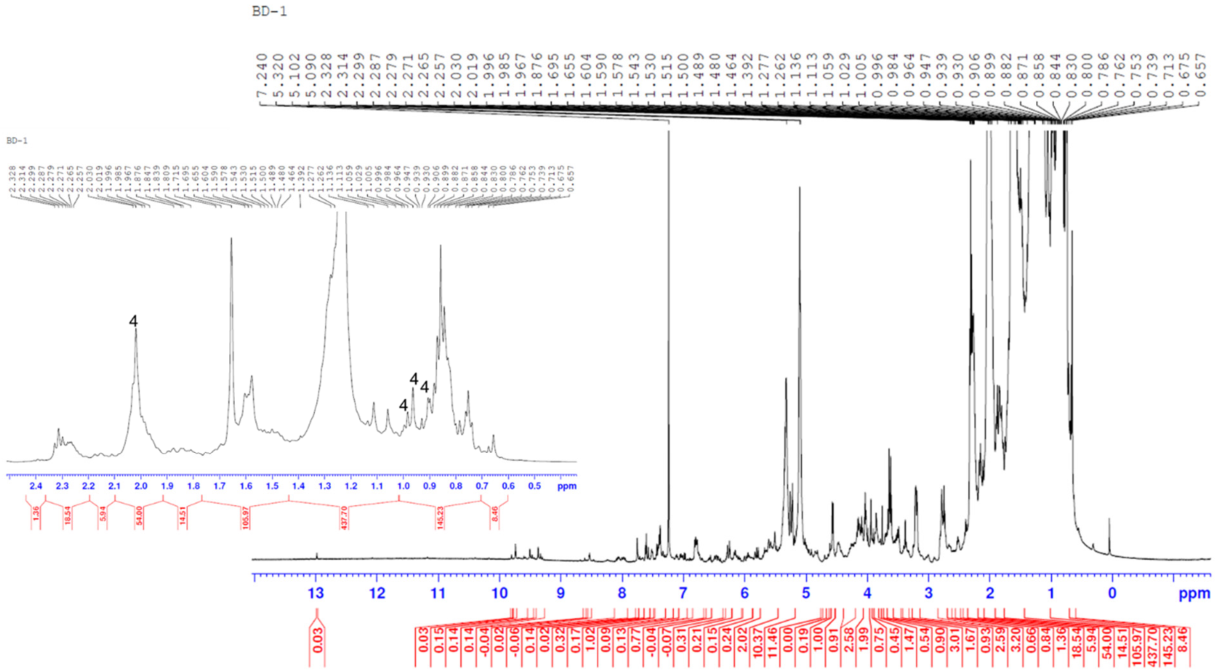

(D) The representative ¹H-NMR spectra of the dichloromethane leaf extract. 4, valine.

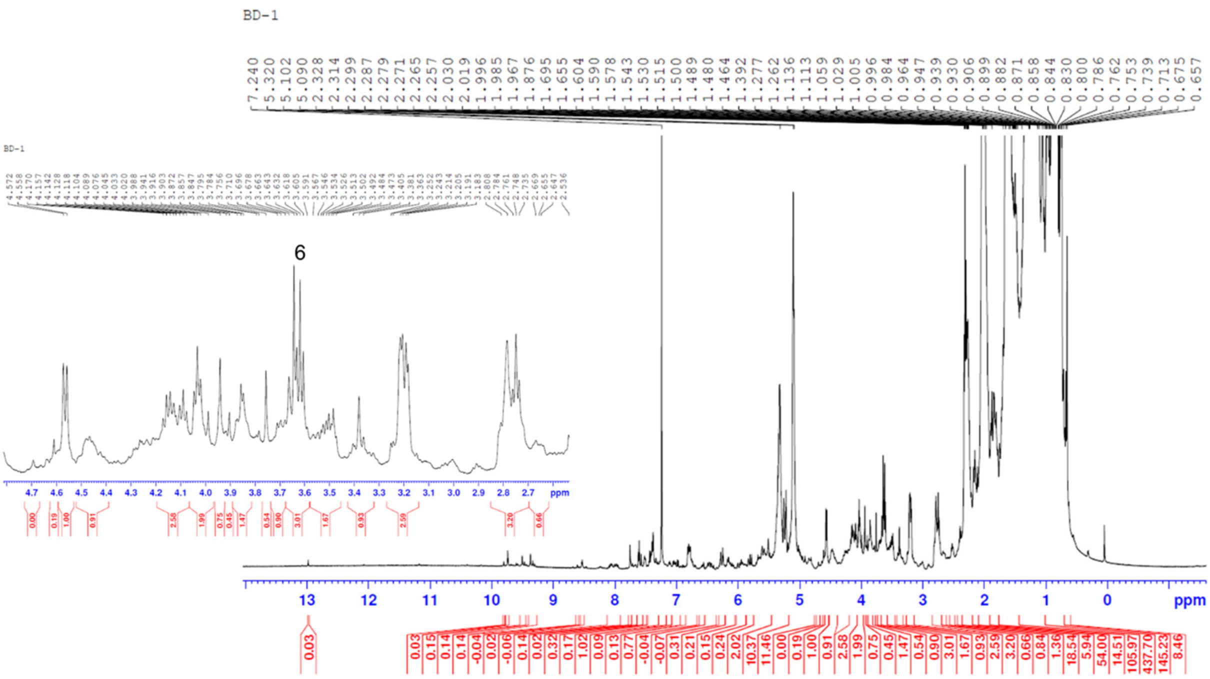

(E) The representative ^{1}H-NMR spectra of the dichloromethane leaf extract. 6, alanine.

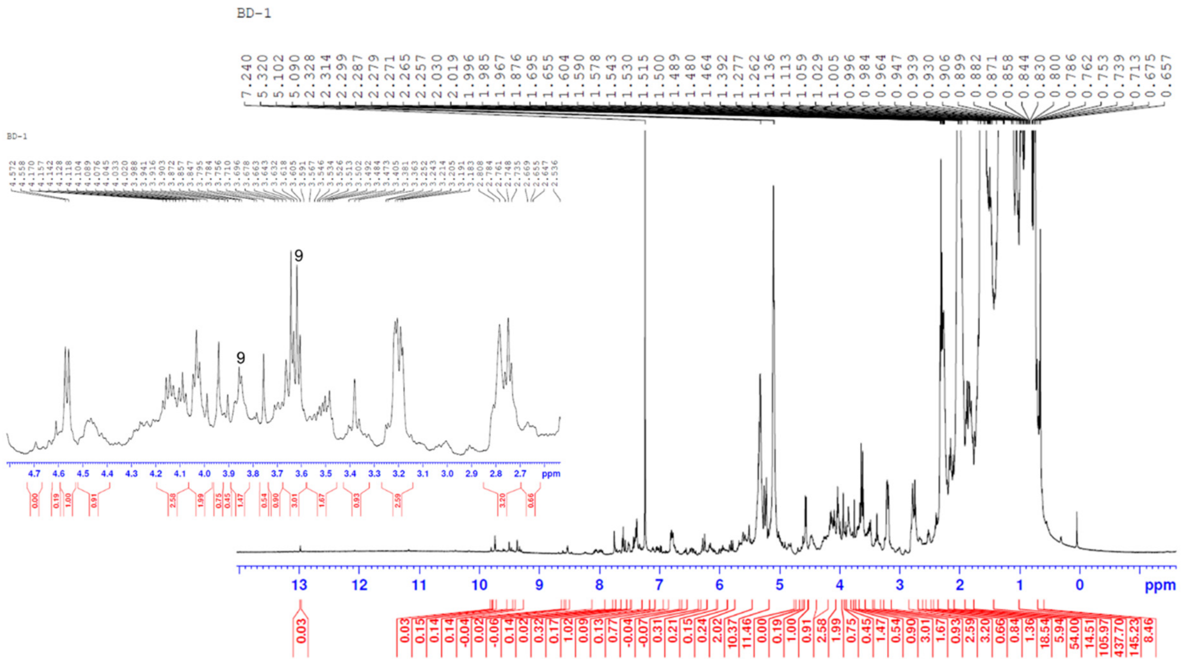

(F) The representative ^{1}H-NMR spectra of the dichloromethane leaf extract. 9, trehalose.

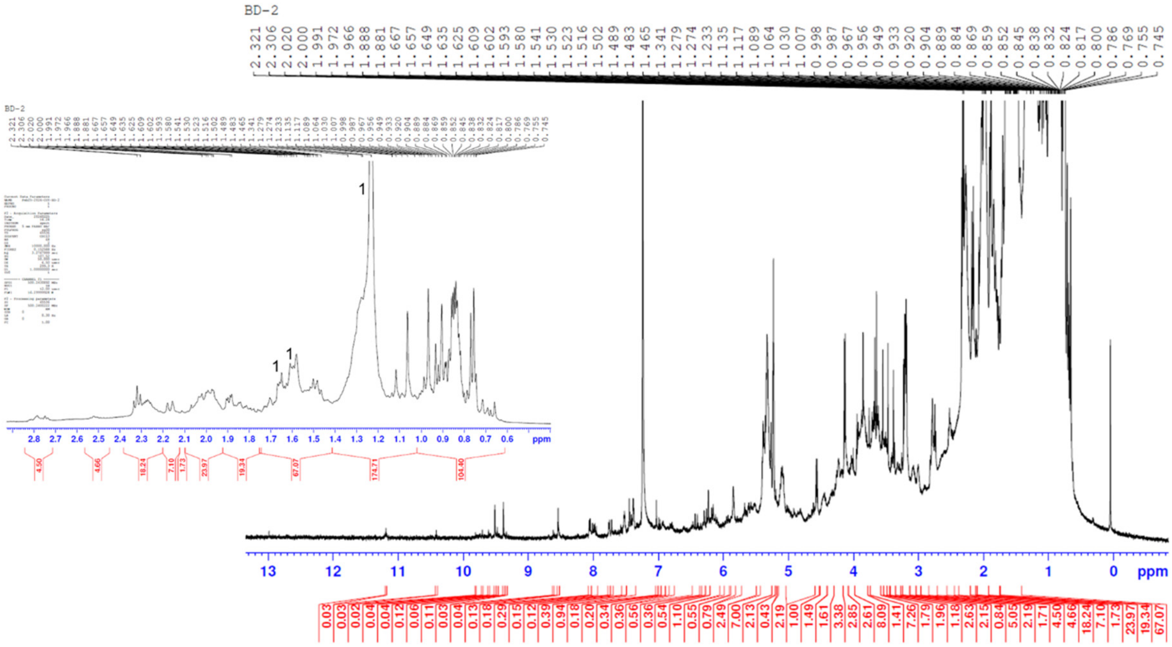

Figure S2. (A) The representative ¹H-NMR spectra of the methanol leaf extract. 1, hexadecanedioic acid.

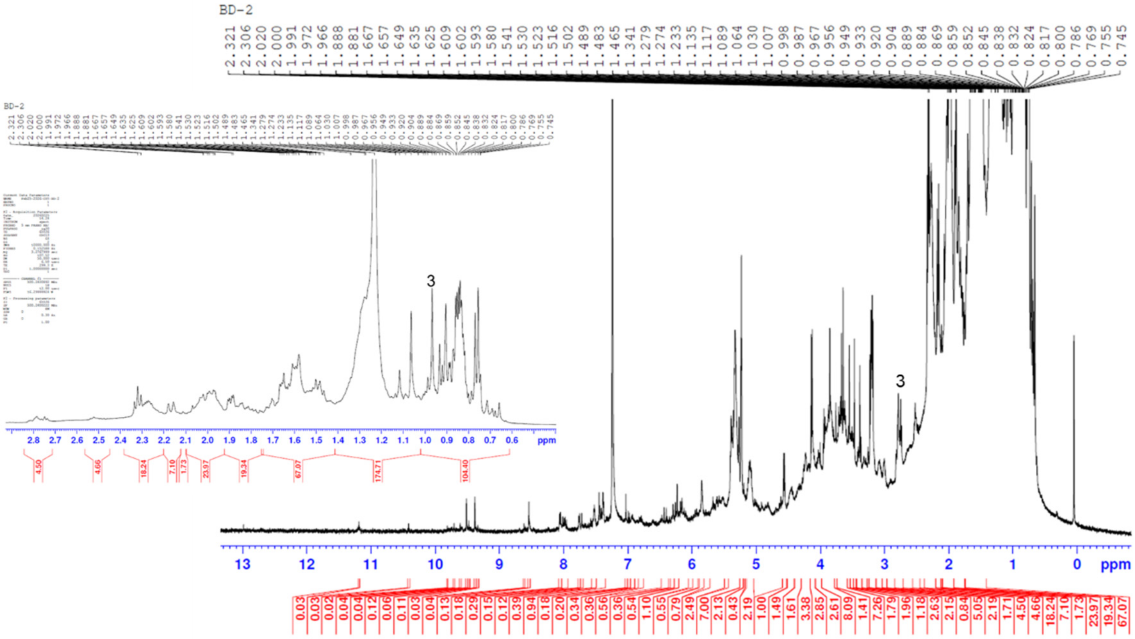

(B) The representative ¹H-NMR spectra of the methanol leaf extract. 3, leucine.

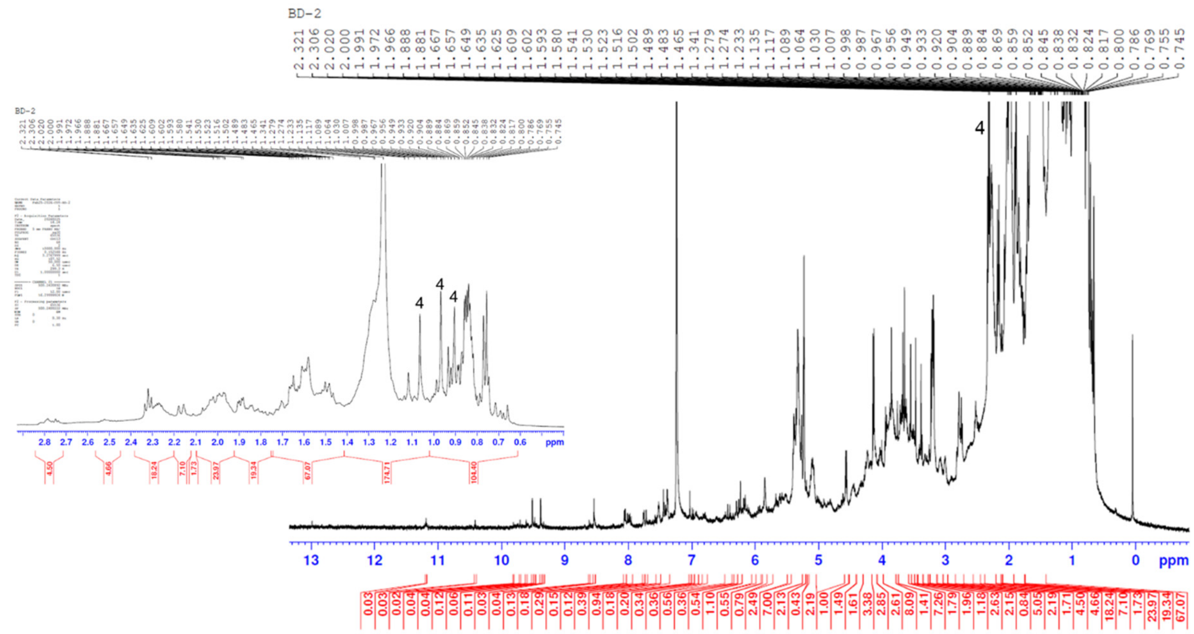

(C) The representative ^{1}H-NMR spectra of the methanol leaf extract. 4, valine.

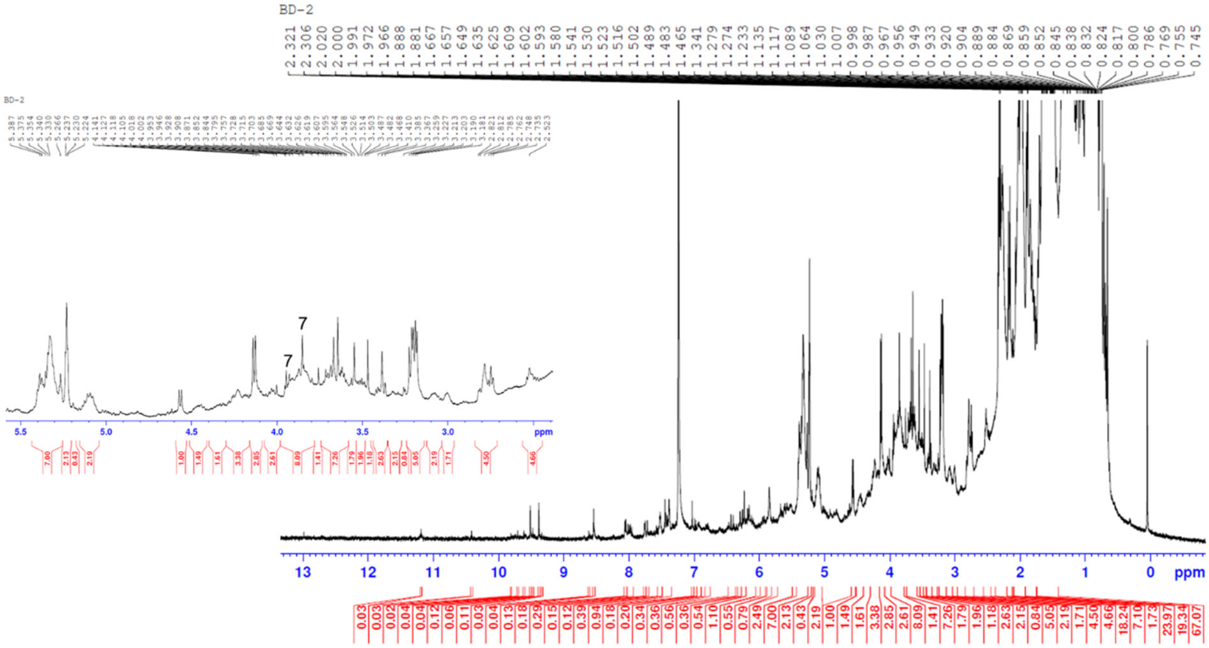

(D) The representative ^{1}H-NMR spectra of the methanol leaf extract. 7, rhamnose.

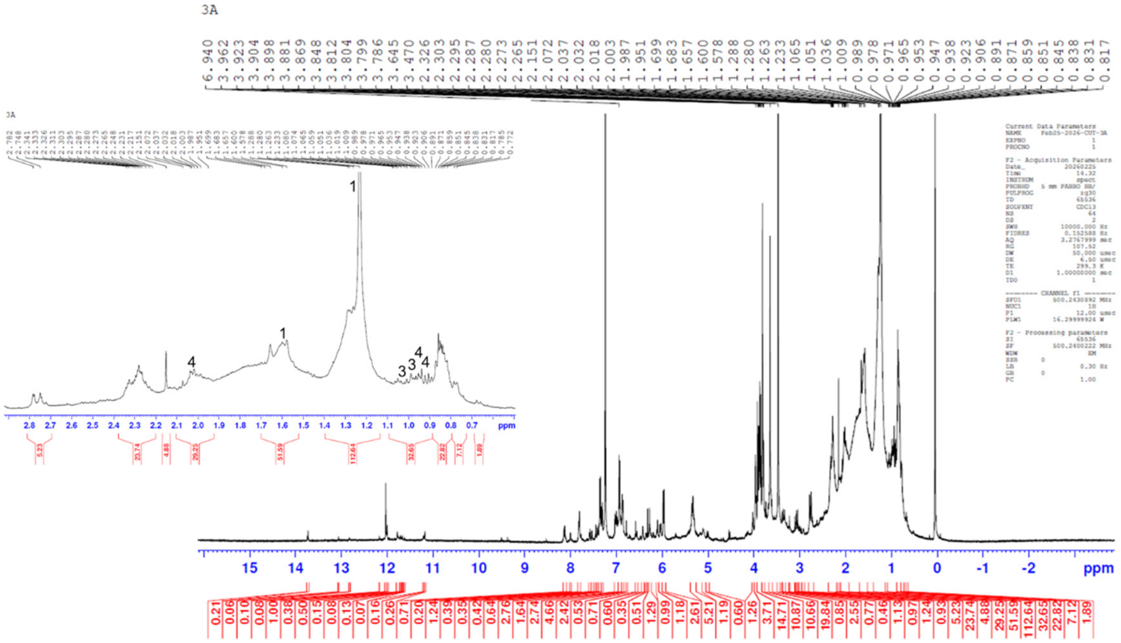

Figure S3. (A) The representative ^{1}H-NMR spectra of the stem bark extract. 1, hexadecanedioic acid; 3, leucine; 4, valine.

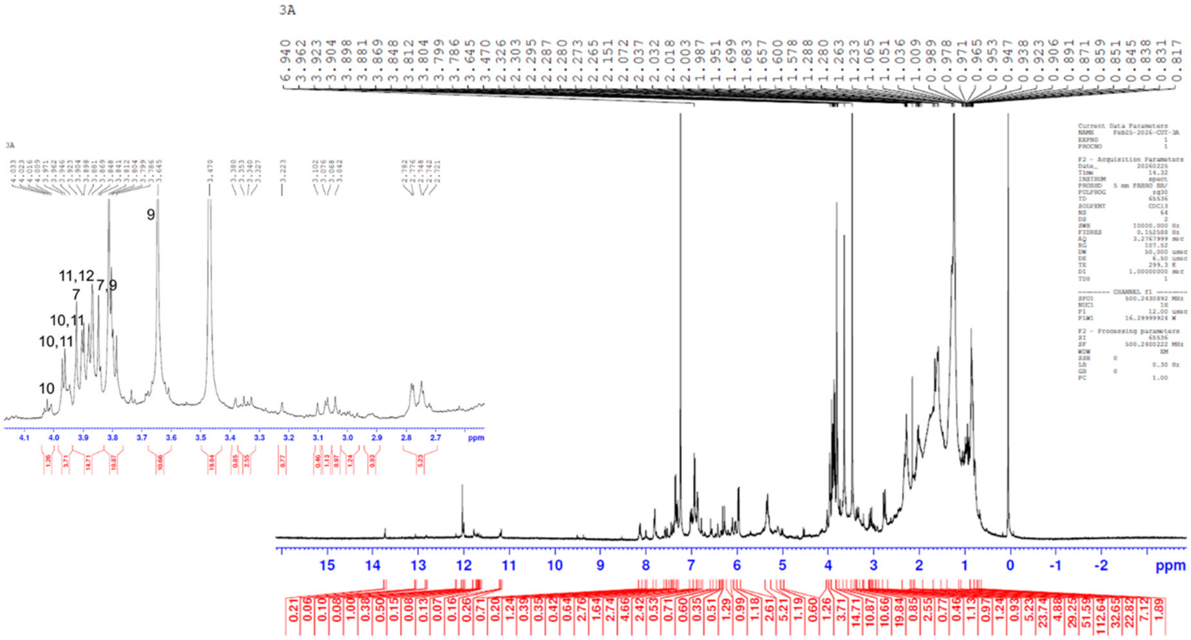

(B) The representative ^{1}H-NMR spectra of the stem bark extract. 7, rhamnose; 8, sucrose; 9, trehalose; 10, fructose; 11, glucose.

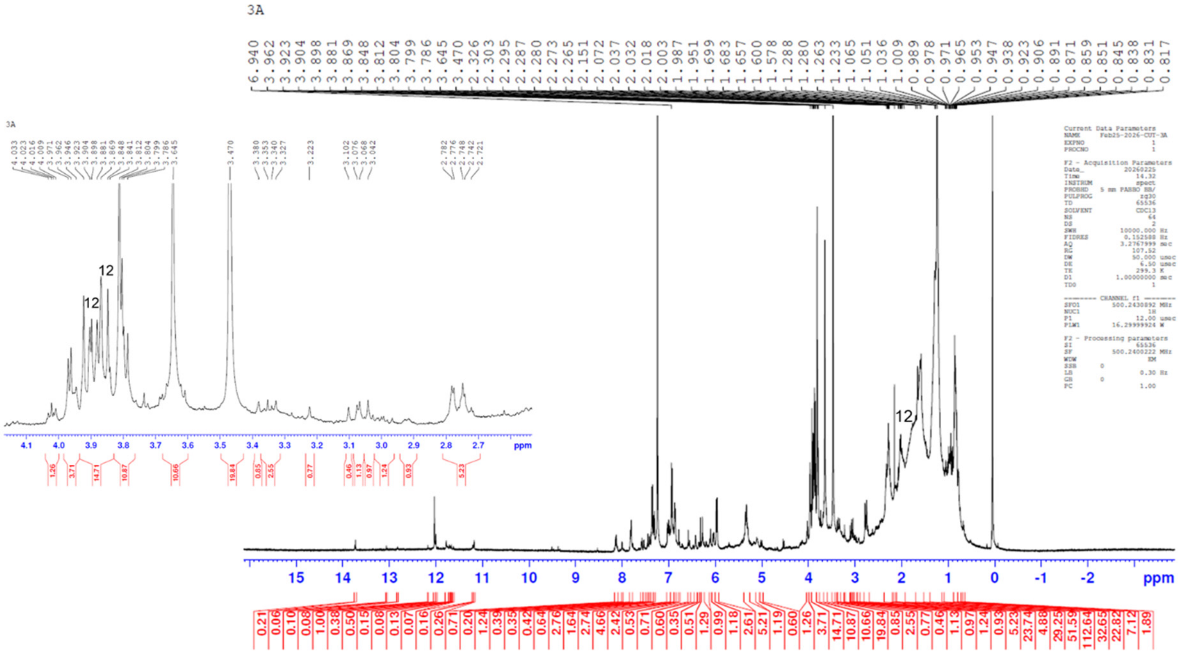

(C) The representative ¹H-NMR spectra of the stem bark extract. 12, chlorogenic acid.

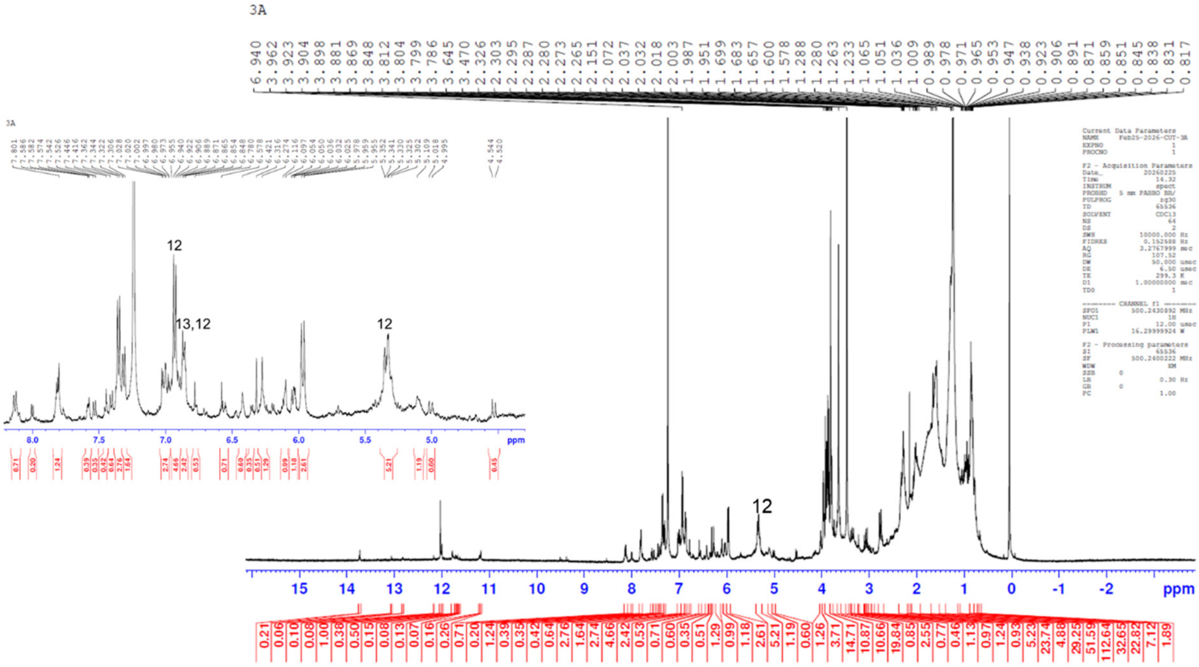

(D) The representative ¹H-NMR spectra of the stem bark extract. 12, chlorogenic acid; 13, quercetin.

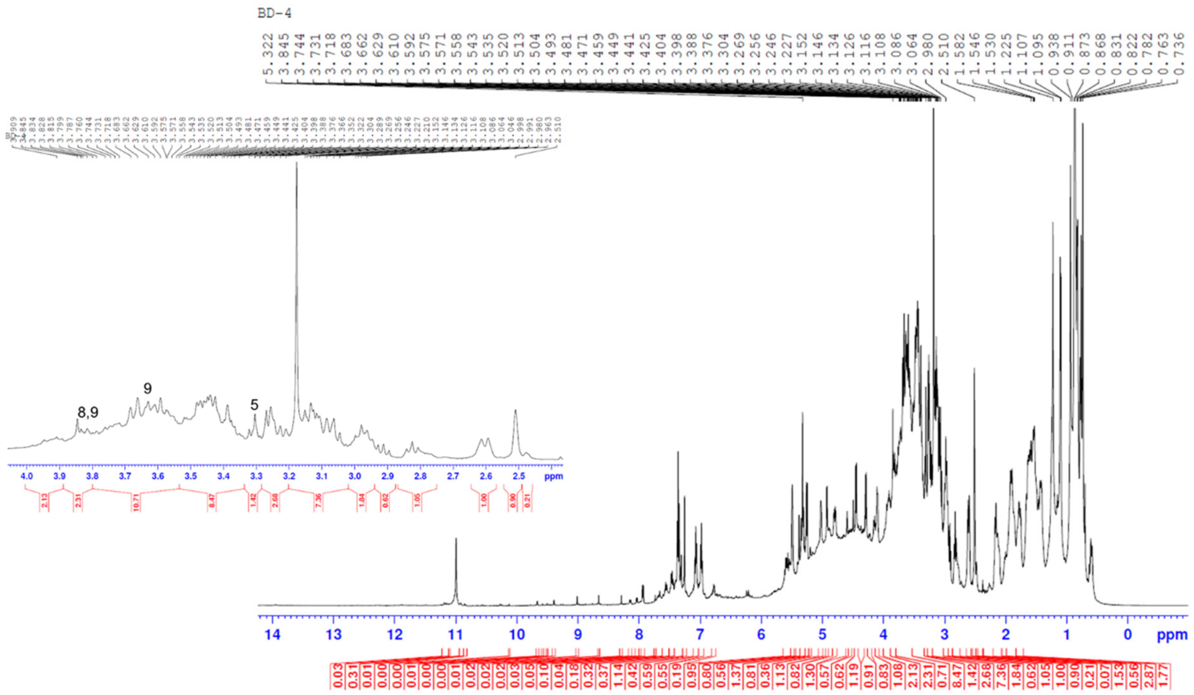

Figure S4. The representative ^{1}H-NMR spectra of the root extract. 5, betaine; 8, sucrose; 9, trehalose.

1

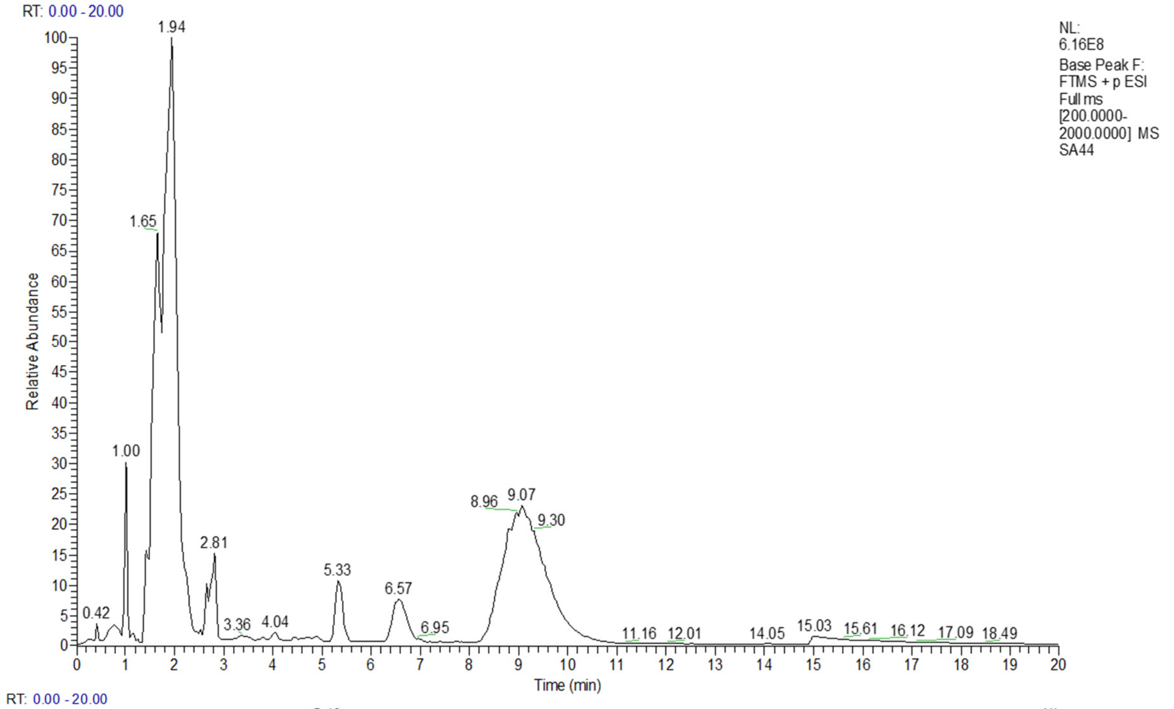
RT: 0.00 - 20.00
NL:
6.16E8
Base Peak F:
FTMS + p ESI
Full ms
[200.0000-
2000.0000] MS
SA44
Relative Abundance
Time (min)
1.94
1.65
1.00
2.81
5.33
6.57
8.96
9.07
9.30
0.42
3.36
4.04
6.95
11.16
12.01
14.05
15.03
15.61
16.12
17.09
18.49

2

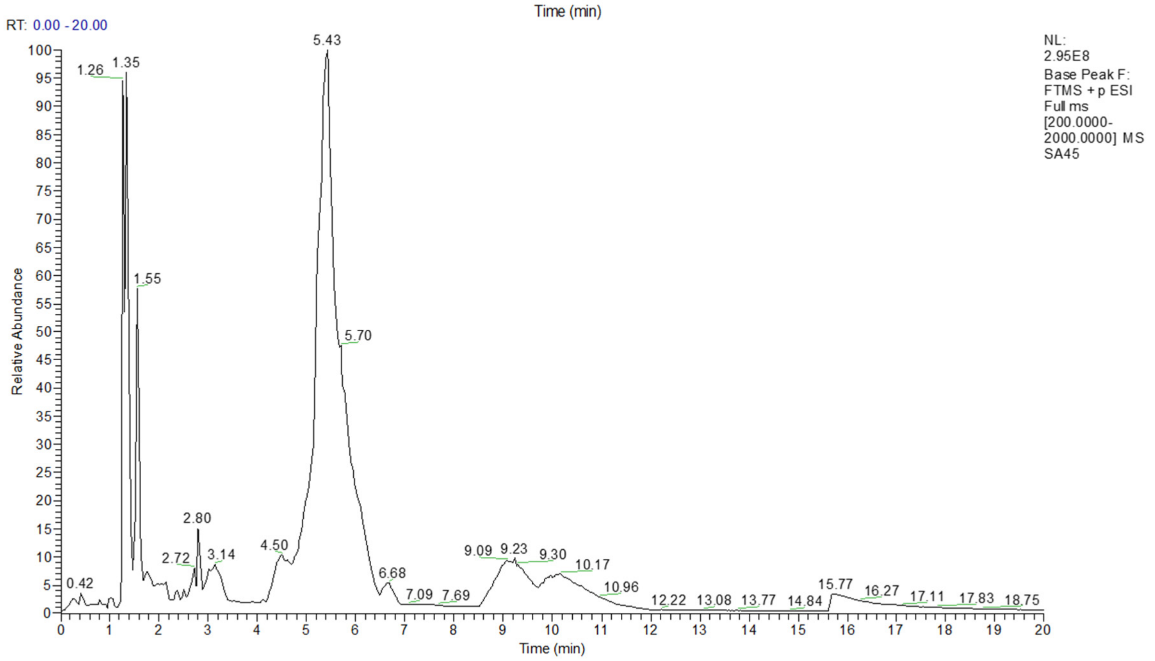
RT: 0.00 - 20.00
NL:
2.95E8
Base Peak F:
FTMS + p ESI
Full ms
[200.0000-
2000.0000] MS
SA45
Relative Abundance
Time (min)
5.43
1.26
1.35
1.55
5.70
2.80
2.72
3.14
4.50
0.42
6.68
7.09
7.69
9.09
9.23
9.30
10.17
10.96
12.22
13.08
13.77
14.84
15.77
16.27
17.11
17.83
18.75

3

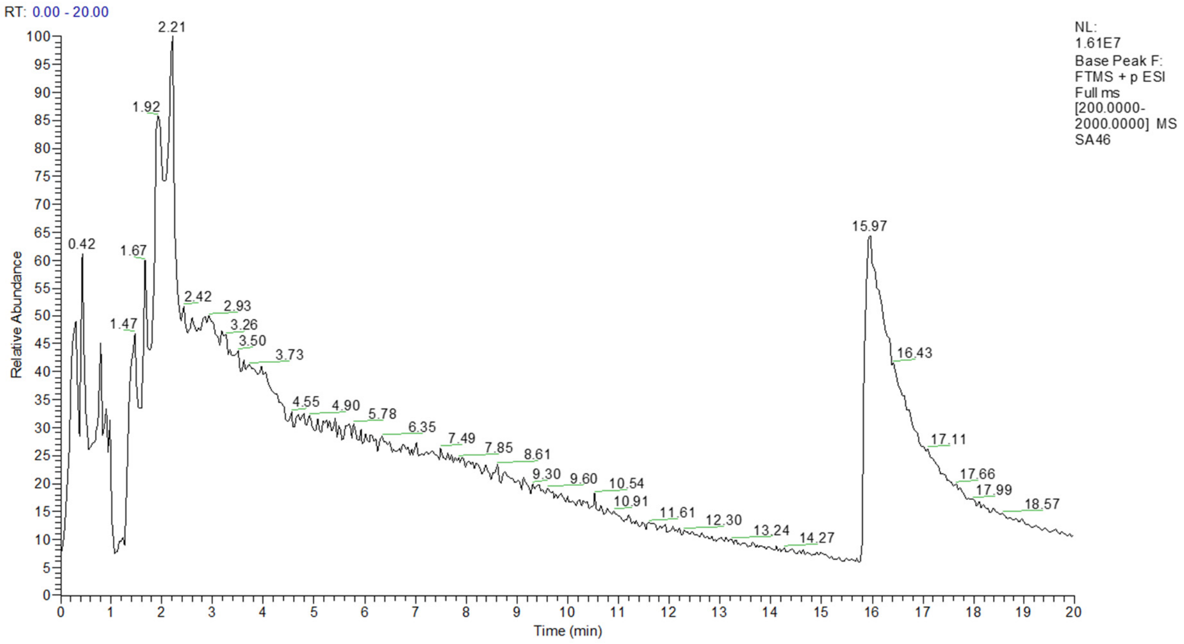

4

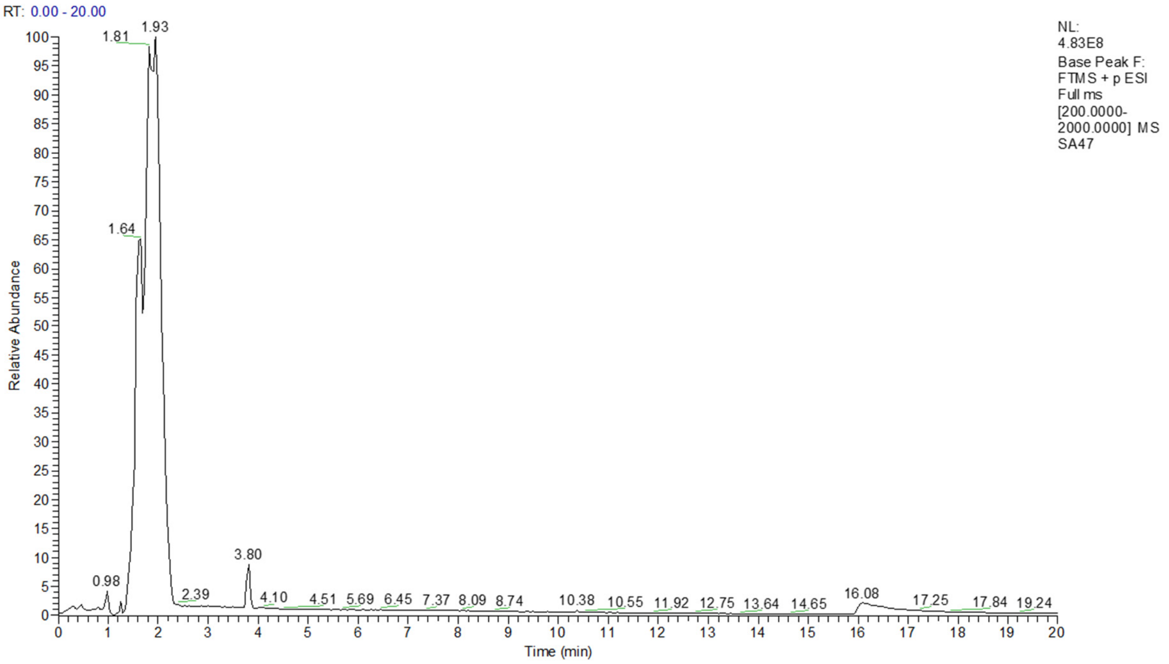

Figure S5. UHPLC–Q/Orbitrap/MS HRMS chromatograms with relative abundance and retention time (min) of (**1**) dichloromethane leaf; (**2**) methanol leaf; (**3**); stem bark and (**4**) root extracts of *Morinda lucida* obtained in positive mode electrospray ionization.

1

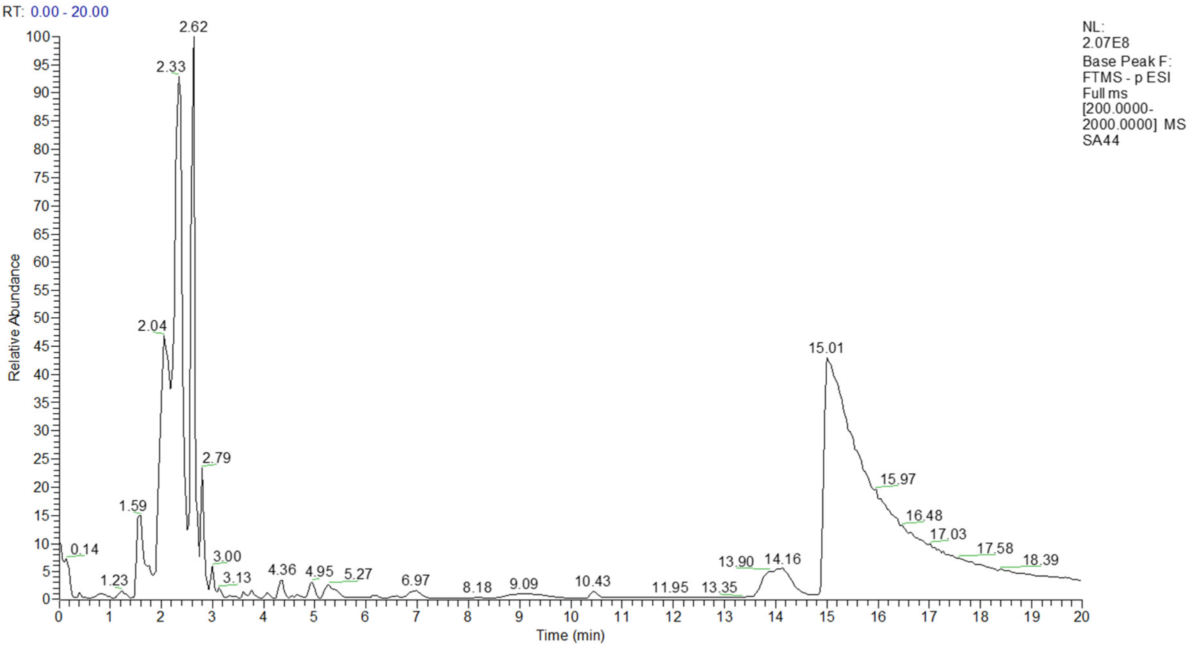

RT: 0.00 - 20.00
NL: 2.07E8 Base Peak F: FTMS - p ESI Full ms [200.0000-2000.0000] MS SA44
Relative Abundance
Time (min)
2.62
2.33
2.04
15.01
2.79
1.59
15.97
16.48
17.03
17.58
18.39
0.14
1.23
3.00
3.13
4.36
4.95
5.27
6.97
8.18
9.09
10.43
11.95
13.35
13.90
14.16

2

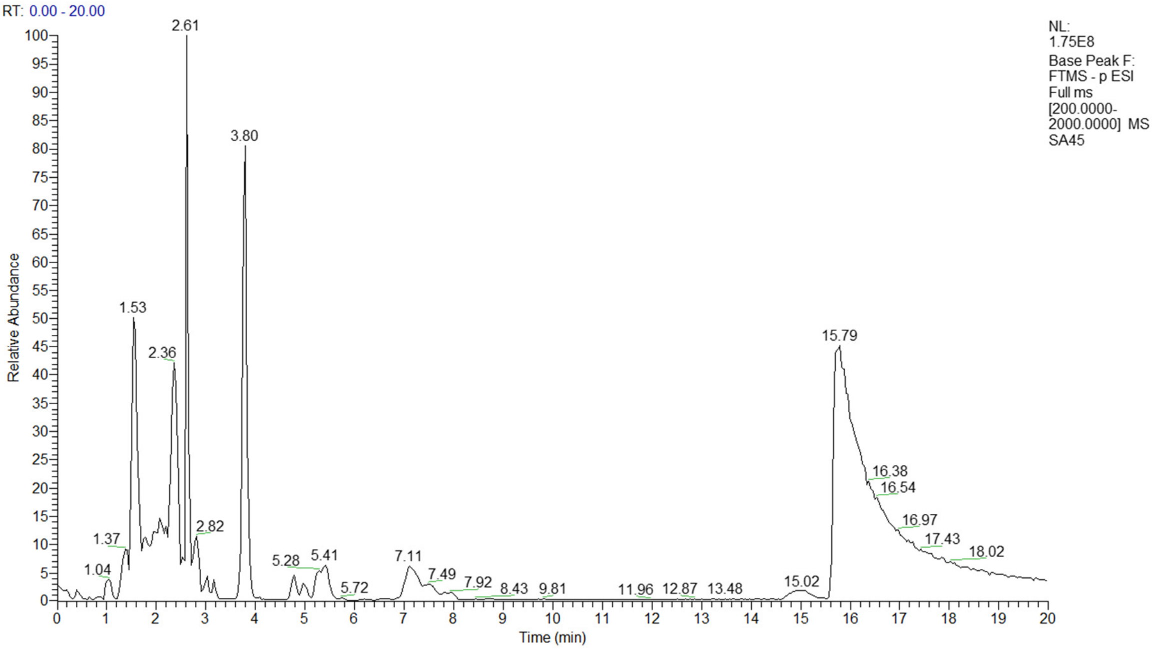

RT: 0.00 - 20.00
NL: 1.75E8 Base Peak F: FTMS - p ESI Full ms [200.0000-2000.0000] MS SA45
Relative Abundance
Time (min)
2.61
3.80
1.53
2.36
15.79
16.38
16.54
16.97
17.43
18.02
1.37
2.82
1.04
5.28
5.41
5.72
7.11
7.49
7.92
8.43
9.81
11.96
12.87
13.48
15.02

3

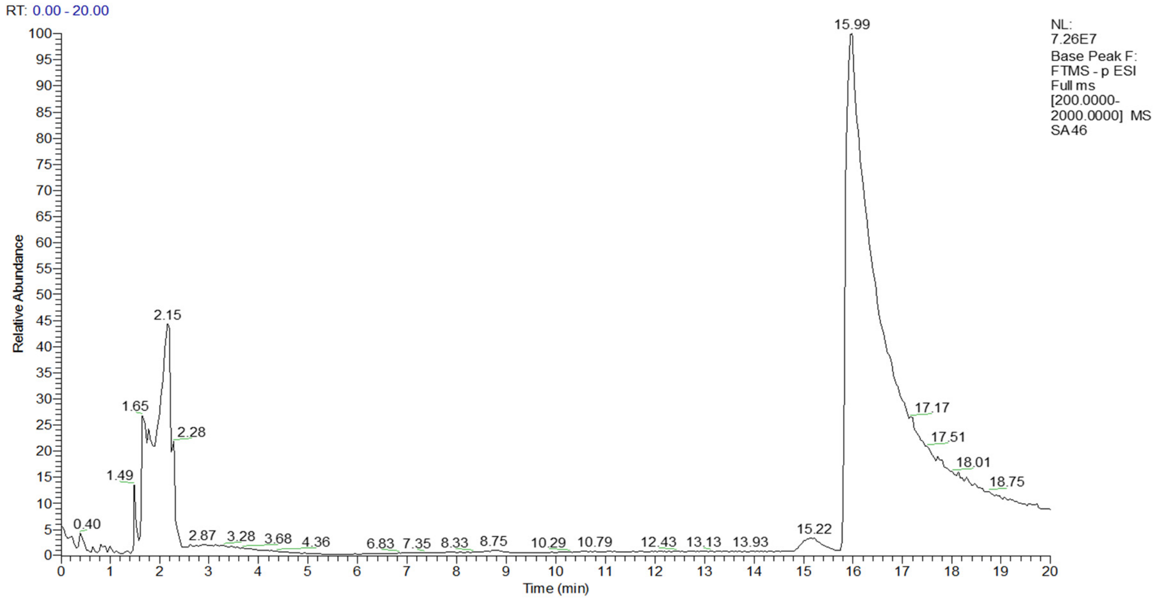

4

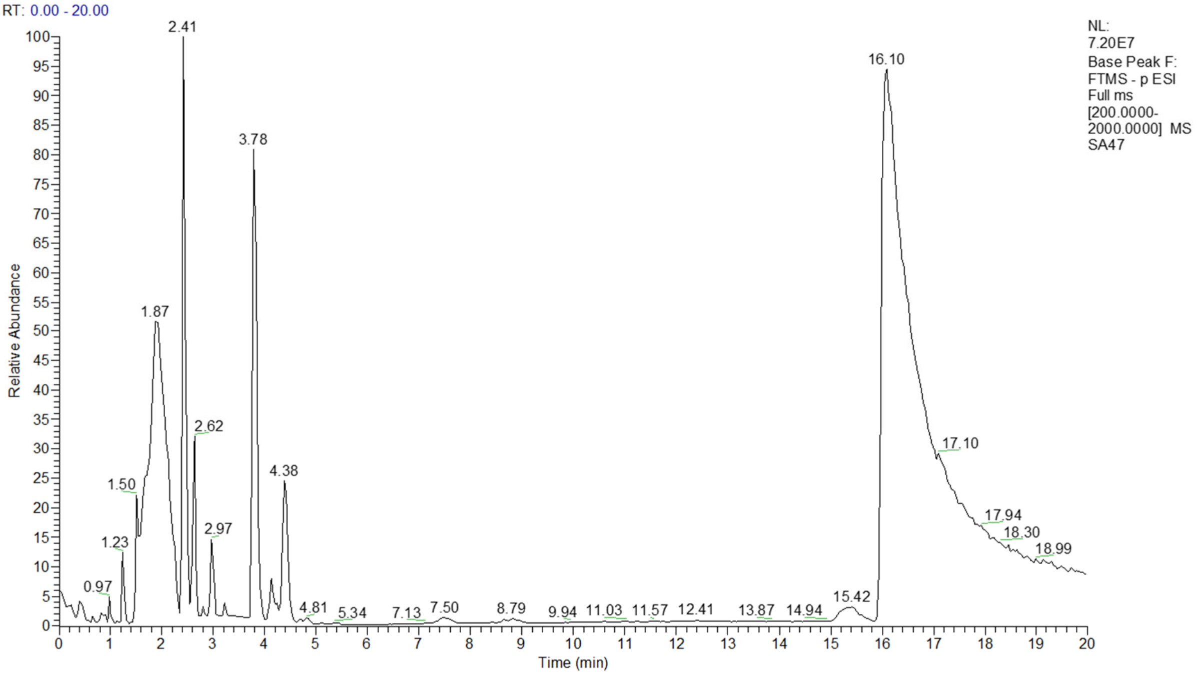

Figure S6. UHPLC–Q/Orbitrap/MS HRMS chromatograms with relative abundance and retention time (min) of (**1**) dichloromethane leaf; (**2**) methanol leaf; (**3**) stem bark; and (**4**) root extracts of *Morinda lucida* obtained in negative mode electrospray ionization.

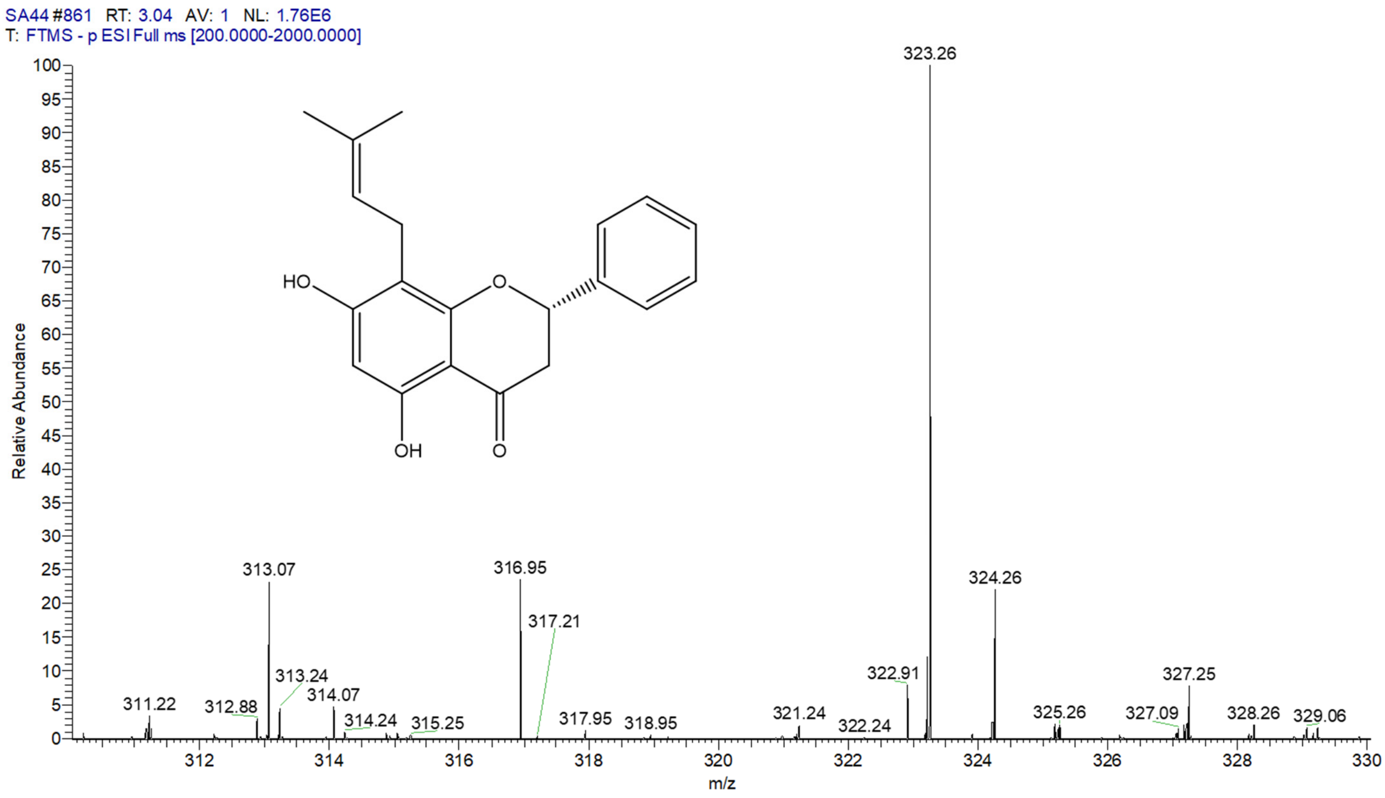

Figure S7. UHPLC–Q/Orbitrap/MS HRMS spectrum showing fragments of glabranine (**1**).

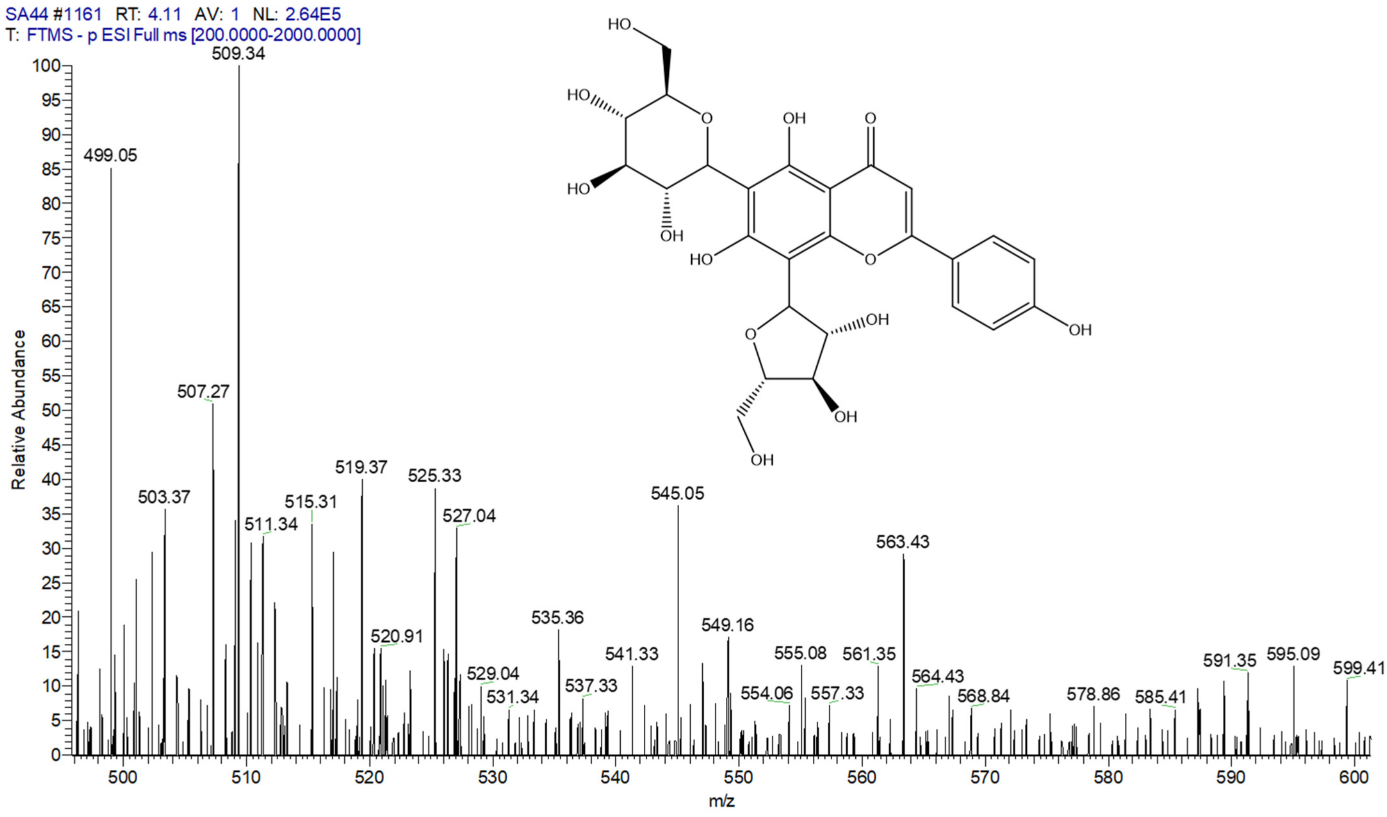

Figure S8. UHPLC–Q/Orbitrap/MS HRMS spectrum showing fragments of apigenin 6-C-glucoside 8-C-arabinoside (**2**).

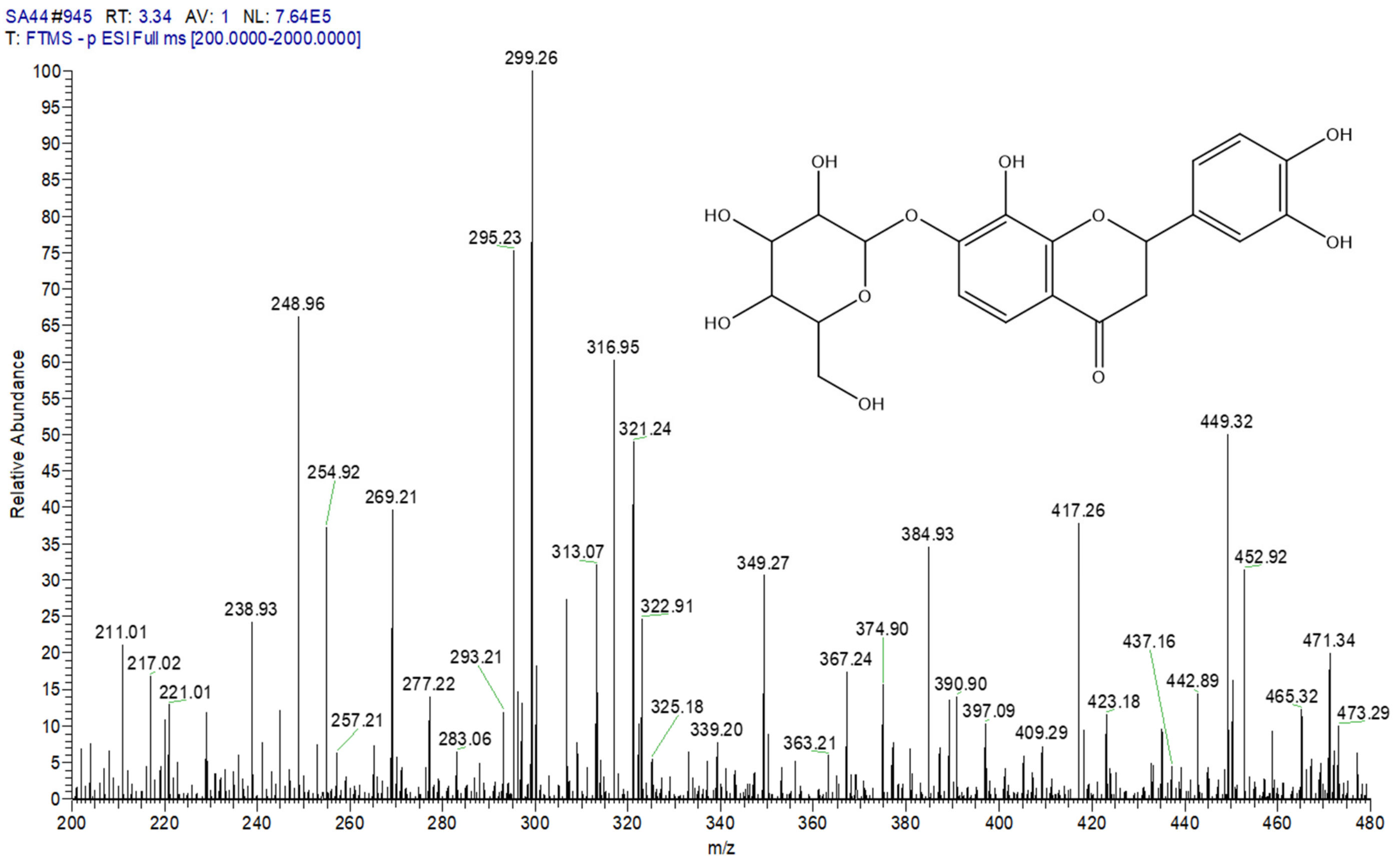

Figure S9. UHPLC–Q/Orbitrap/MS HRMS spectrum showing fragments of isookanin-7-*O*-glucoside (**3**).

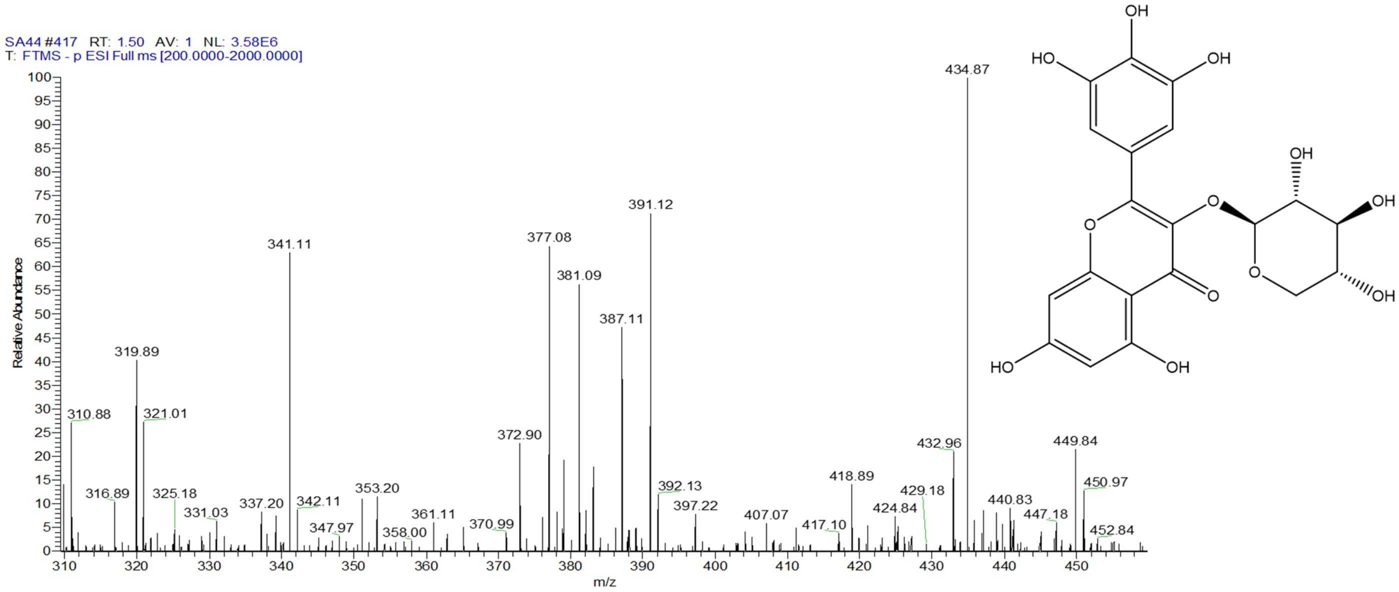

Figure S10. UHPLC–Q/Orbitrap/MS HRMS spectrum showing fragments of myricetin-3-*O*-xyloside (**4**).

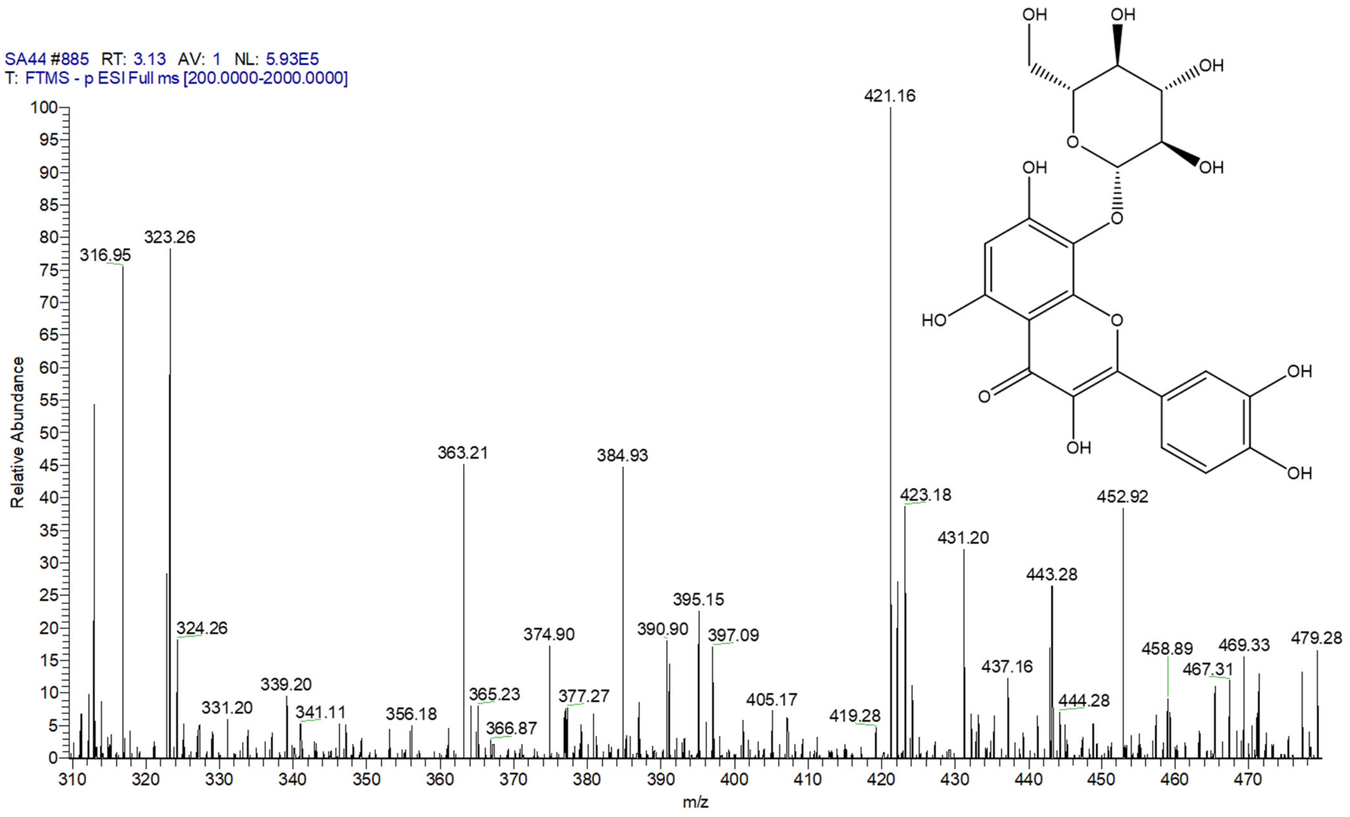

Figure S11. UHPLC–Q/Orbitrap/MS HRMS spectrum showing fragments of gossypetin-8-C-glucoside (**5**).

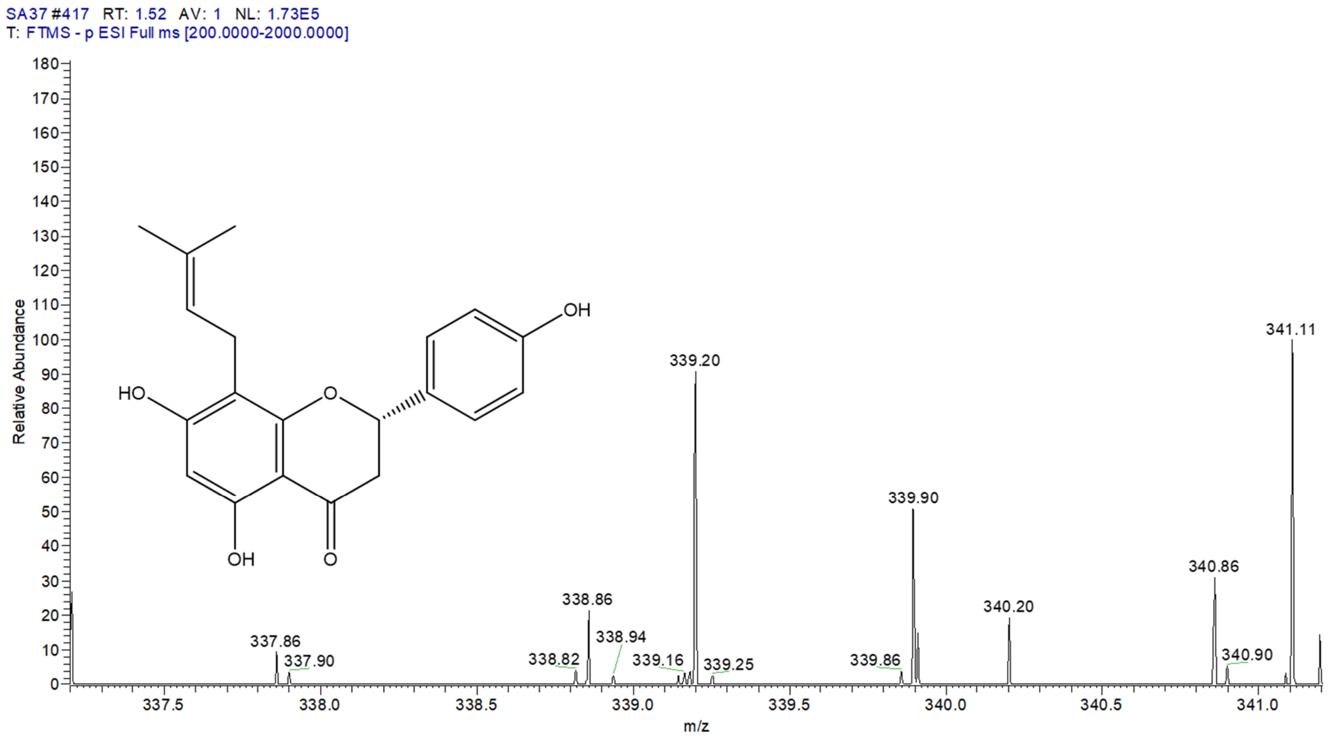

Figure S12. UHPLC–Q/Orbitrap/MS HRMS spectrum showing fragments of 8-prenylnaringenin (**6**).

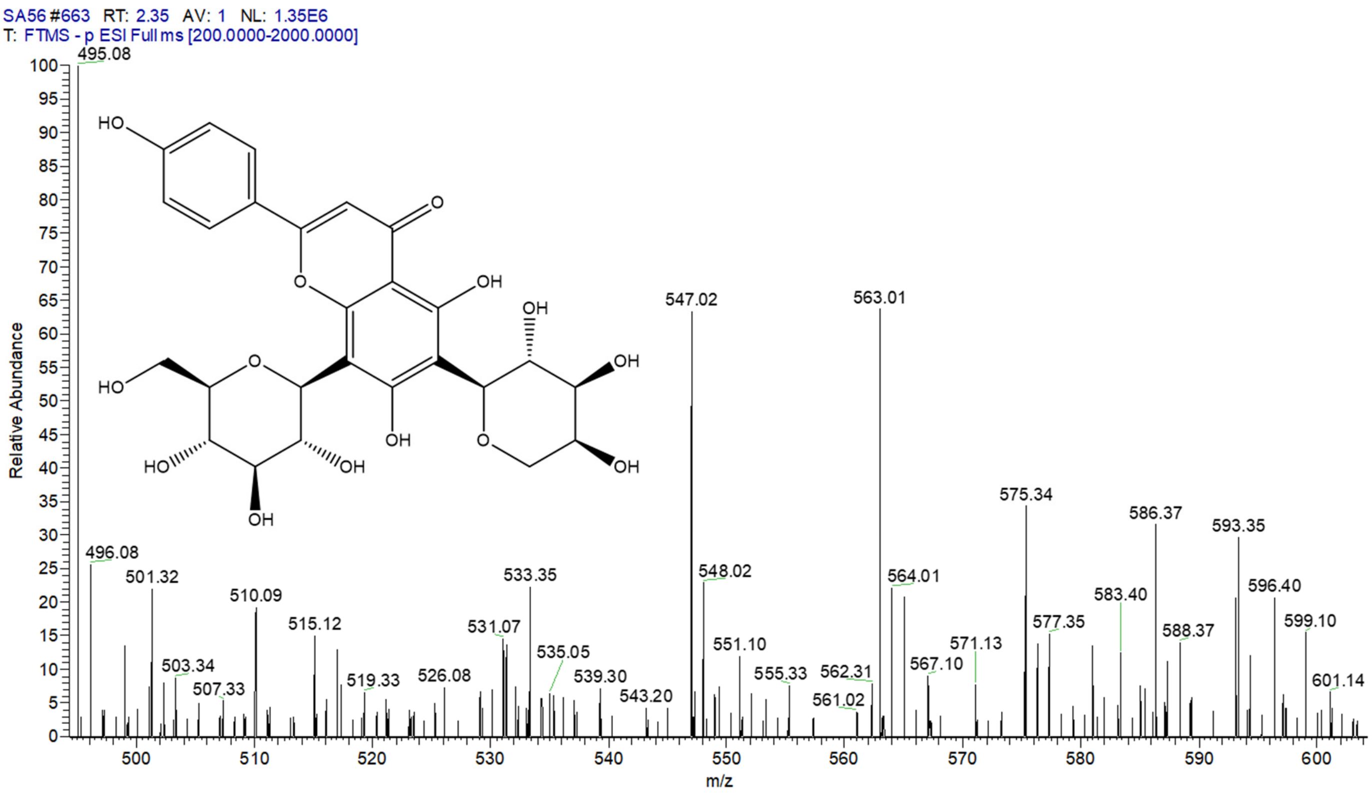

Figure S13. UHPLC–Q/Orbitrap/MS HRMS spectrum showing fragments of isoschaftoside (**7**).

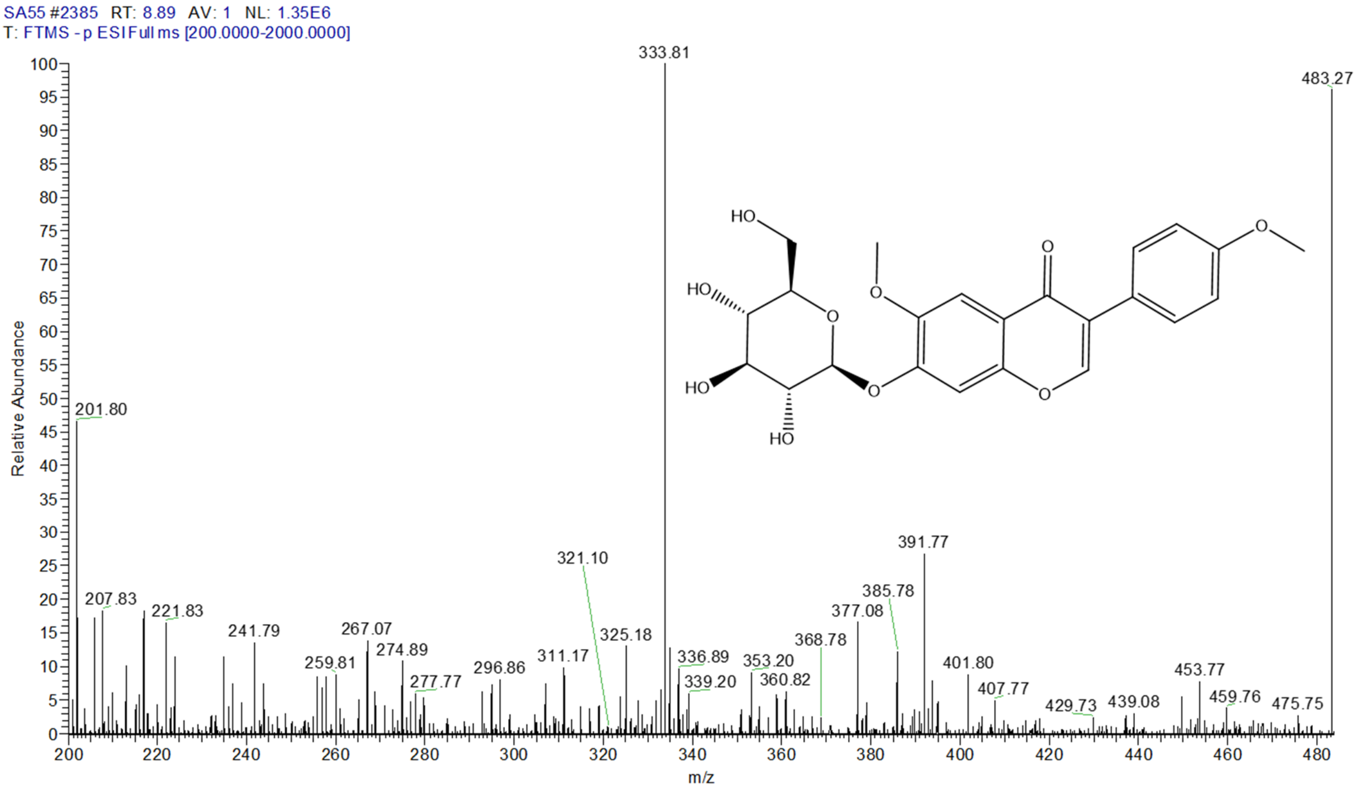

Figure S14. UHPLC–Q/Orbitrap/MS HRMS spectrum showing fragments of 6,4'-dimethoxyisoflavone-7-glucoside (Wistin) (**8**).

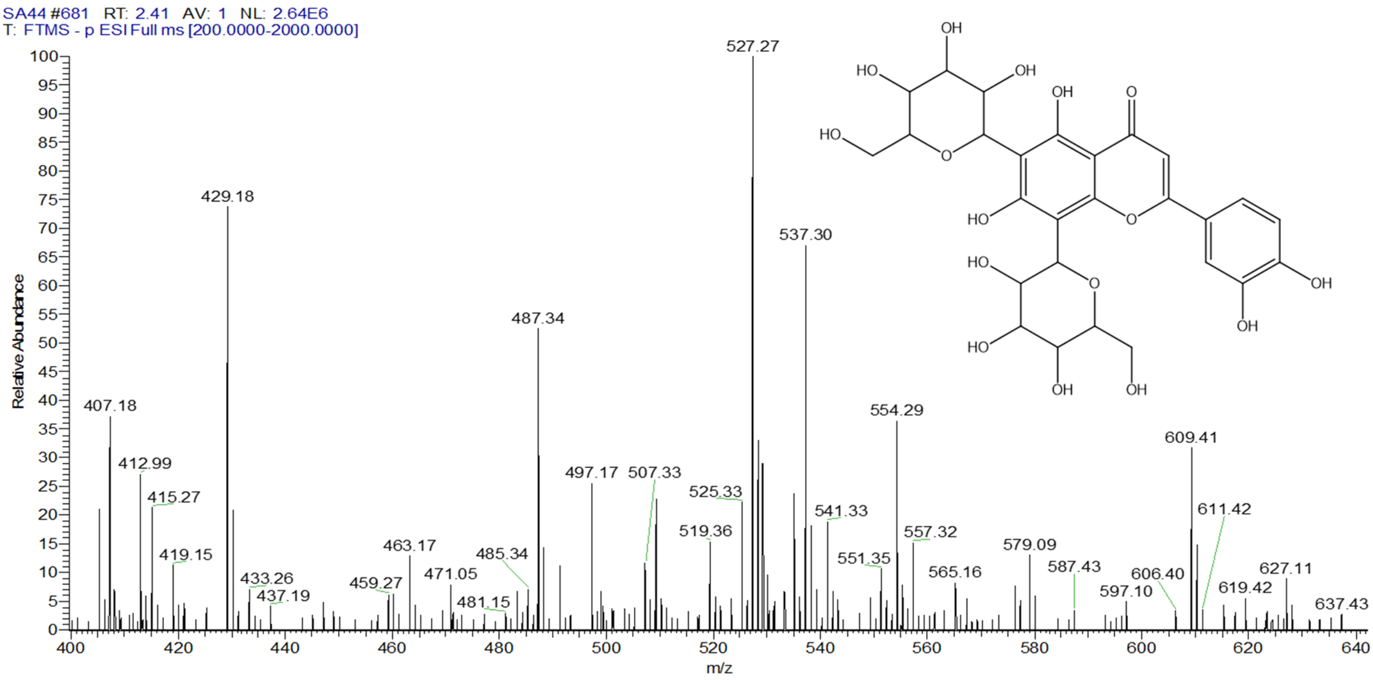

Figure S15. UHPLC–Q/Orbitrap/MS HRMS spectrum showing fragments of luteolin 6-C-glucoside 8-C-arabinoside (**9**).

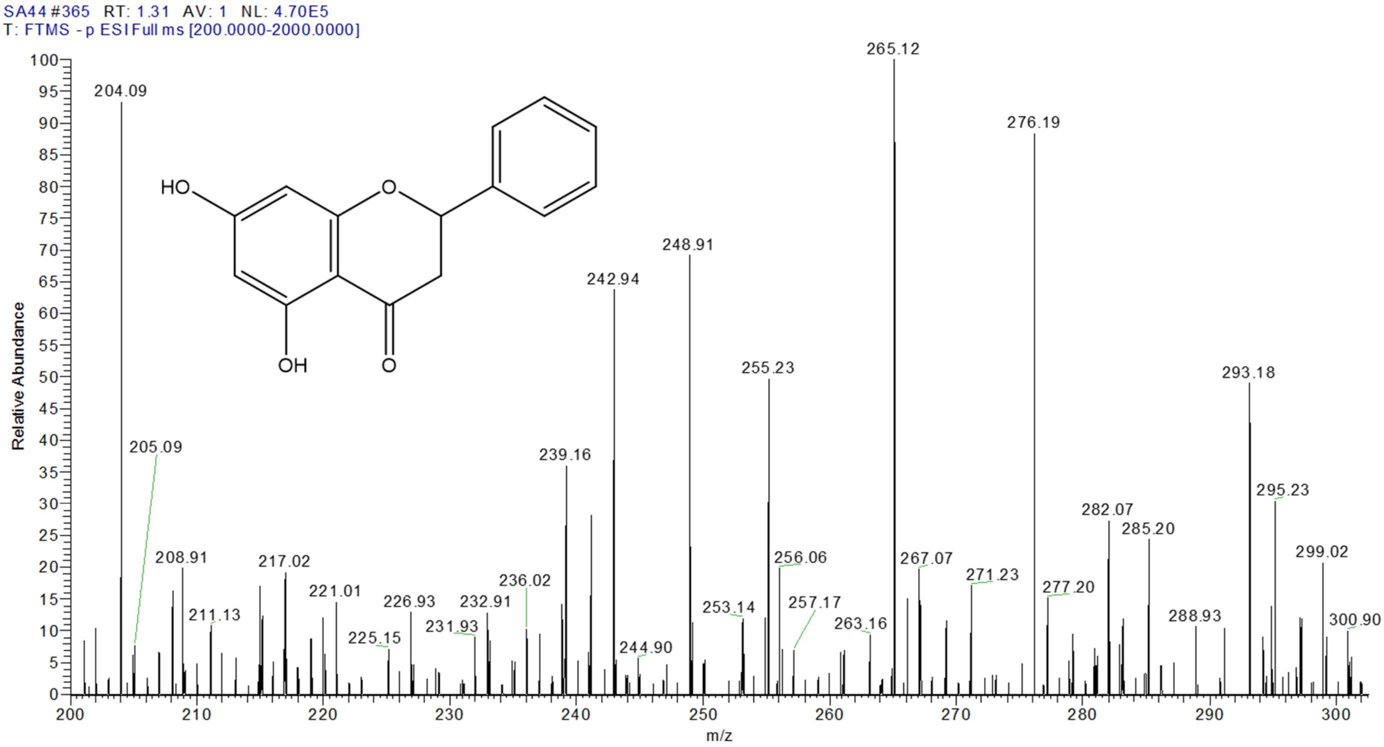

Figure S16. UHPLC–Q/Orbitrap/MS HRMS spectrum showing fragments of pinocembrin (**10**).

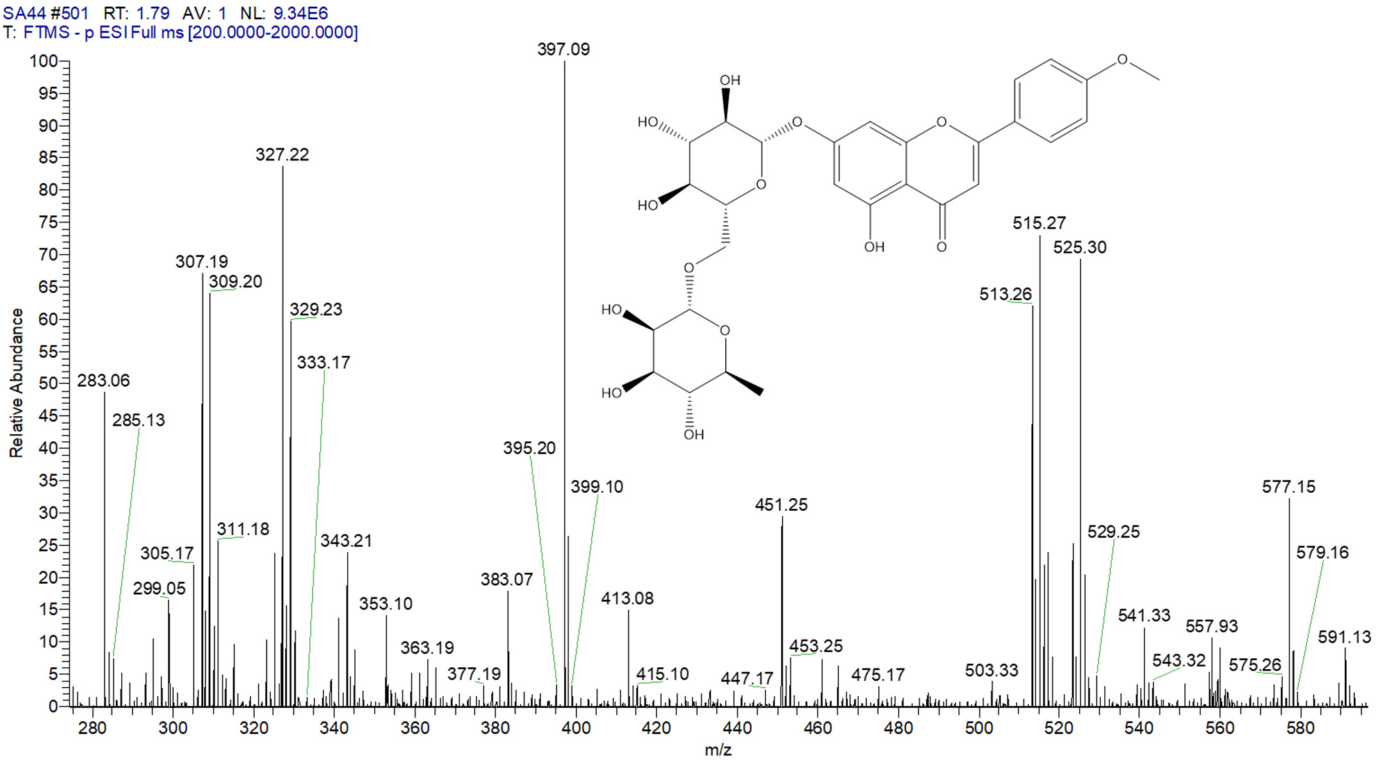

Figure S17. UHPLC–Q/Orbitrap/MS HRMS spectrum showing fragments of acacetin-7-*O*-rutinoside (Linarin) (**11**).

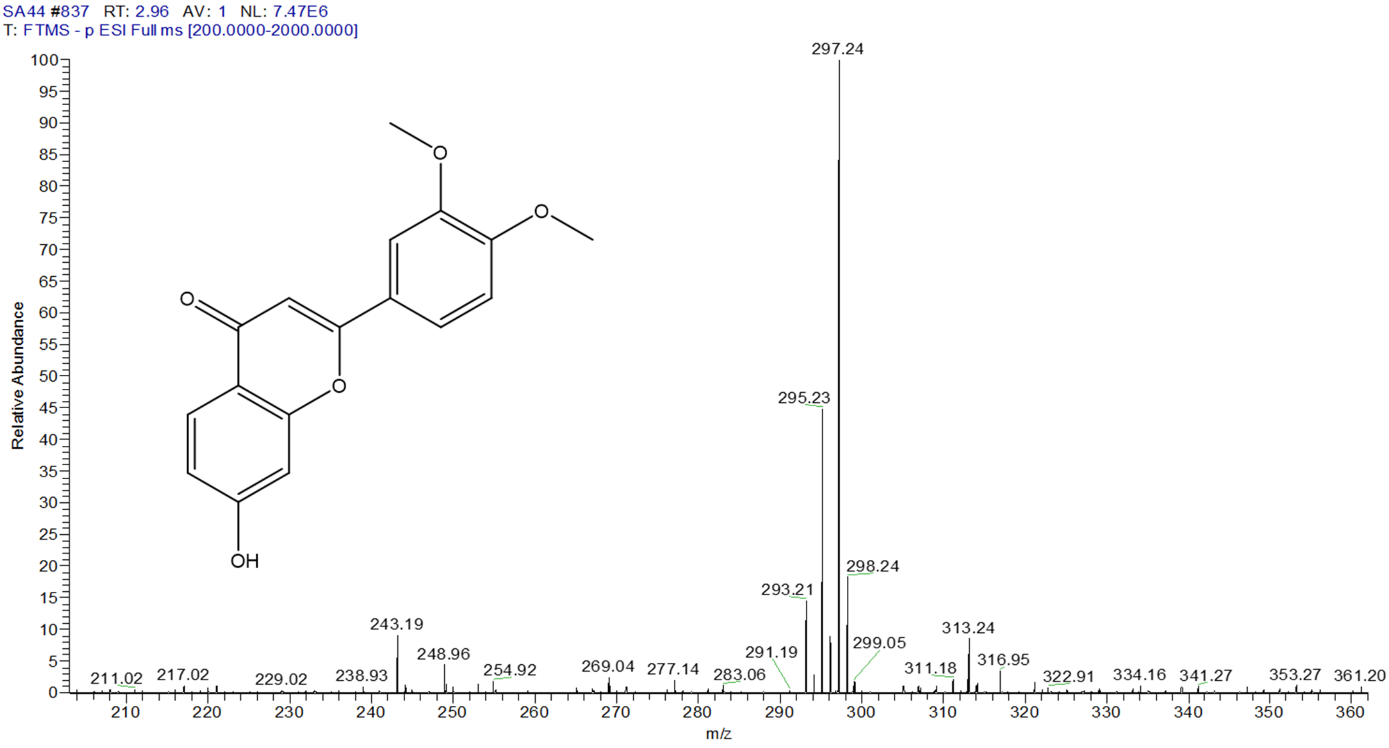

Figure S18. UHPLC–Q/Orbitrap/MS HRMS spectrum showing fragments of 3',4'-dimethoxy-7-hydroxyflavone (**12**).

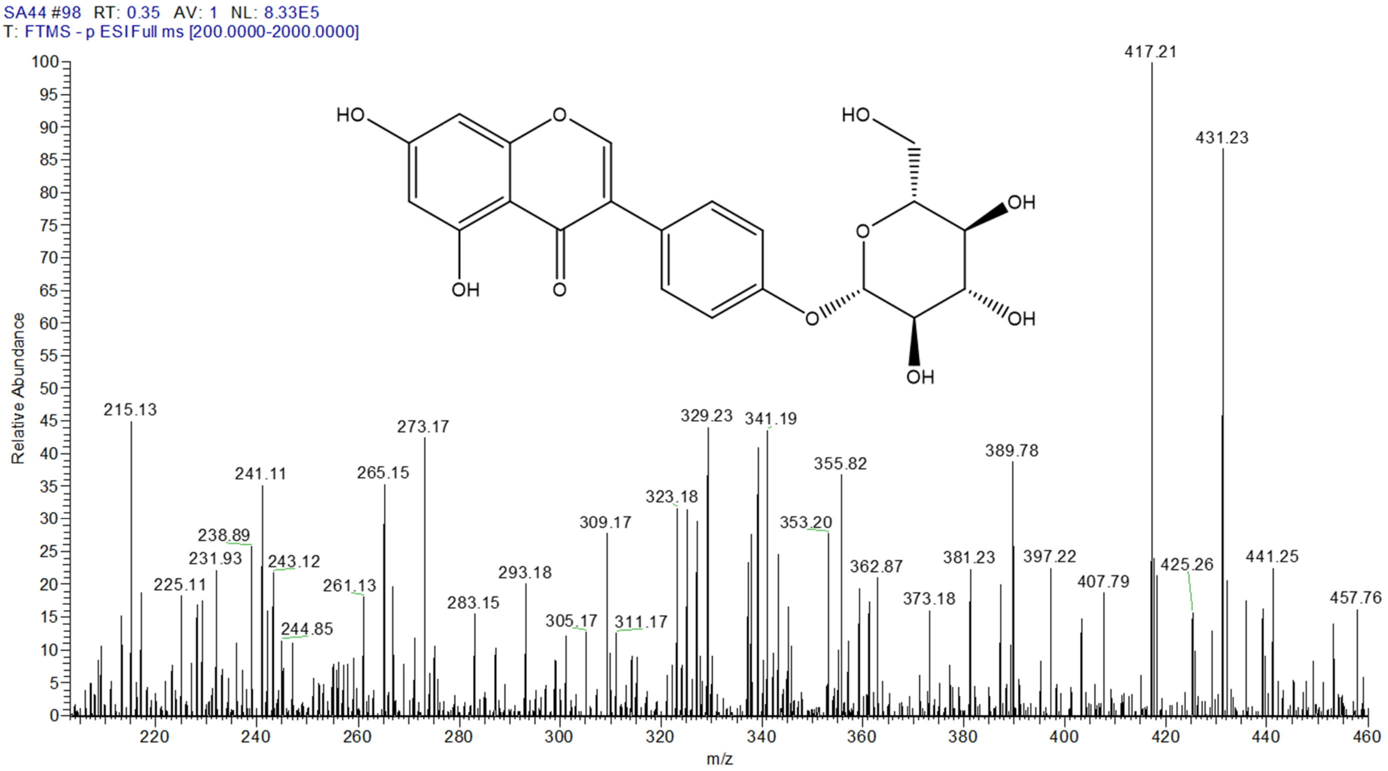

Figure S19. UHPLC–Q/Orbitrap/MS HRMS spectrum showing fragments of sophoricoside (**13**).

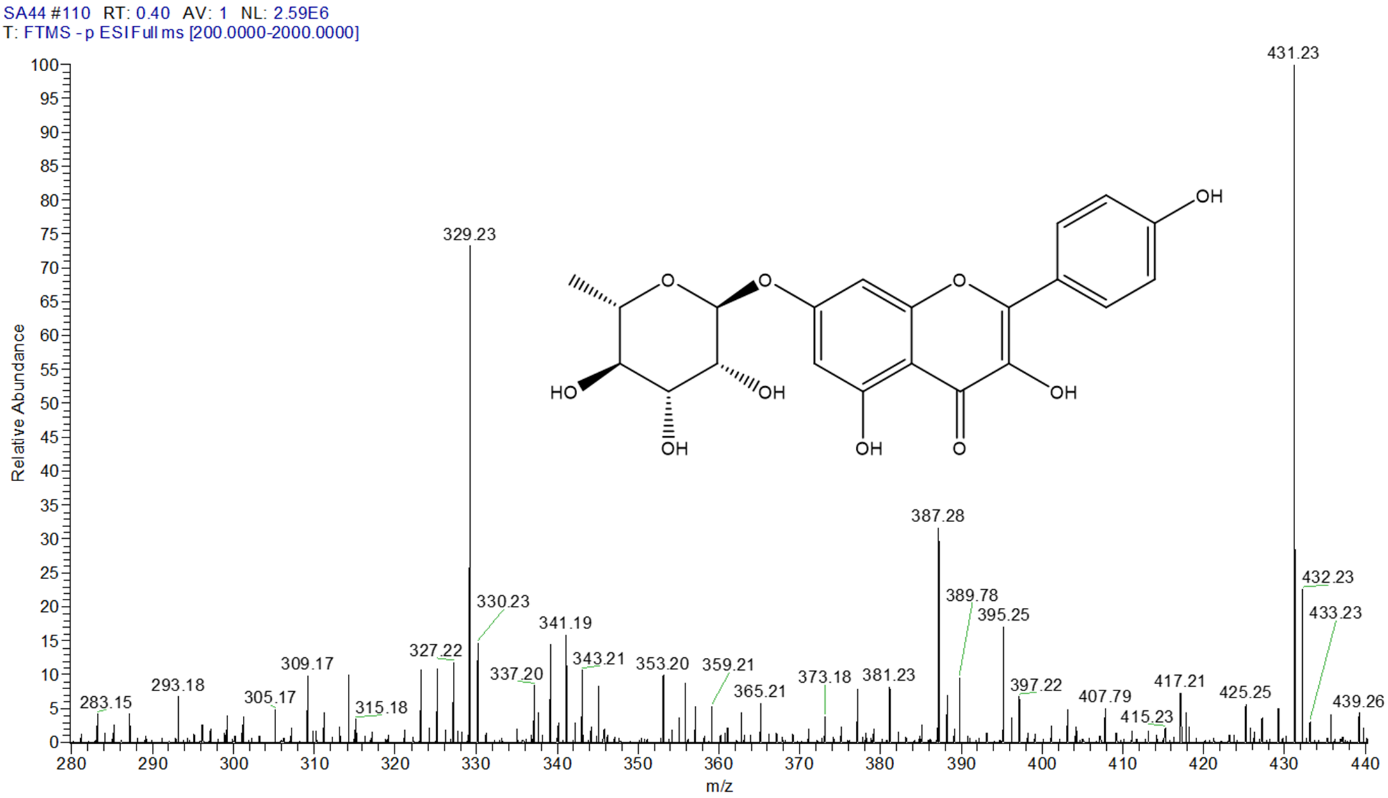

Figure S20. UHPLC–Q/Orbitrap/MS HRMS spectrum showing fragments of kaempferol-7-*O*-rhamnoside (**14**).

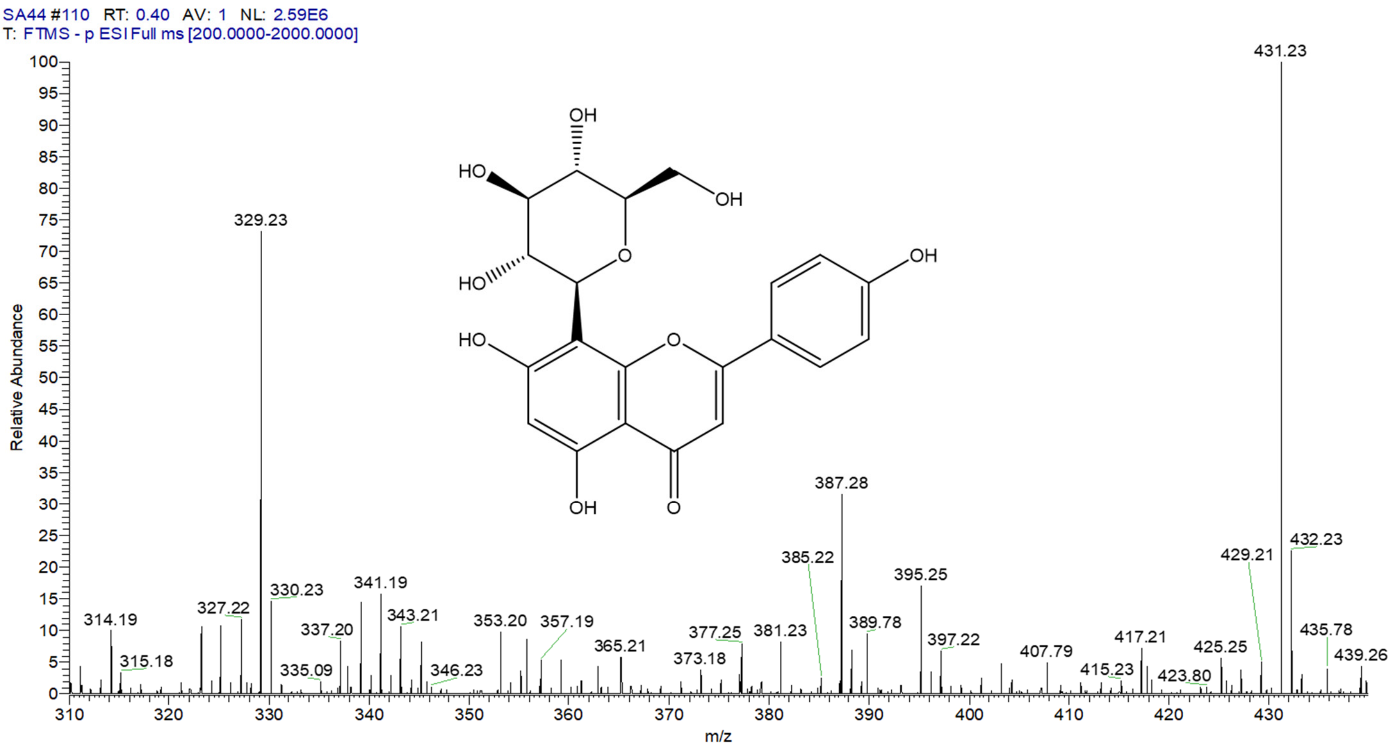

Figure S21. UHPLC–Q/Orbitrap/MS HRMS spectrum showing fragments of apigenin 8-C-glucoside (Vitexin) (**15**).

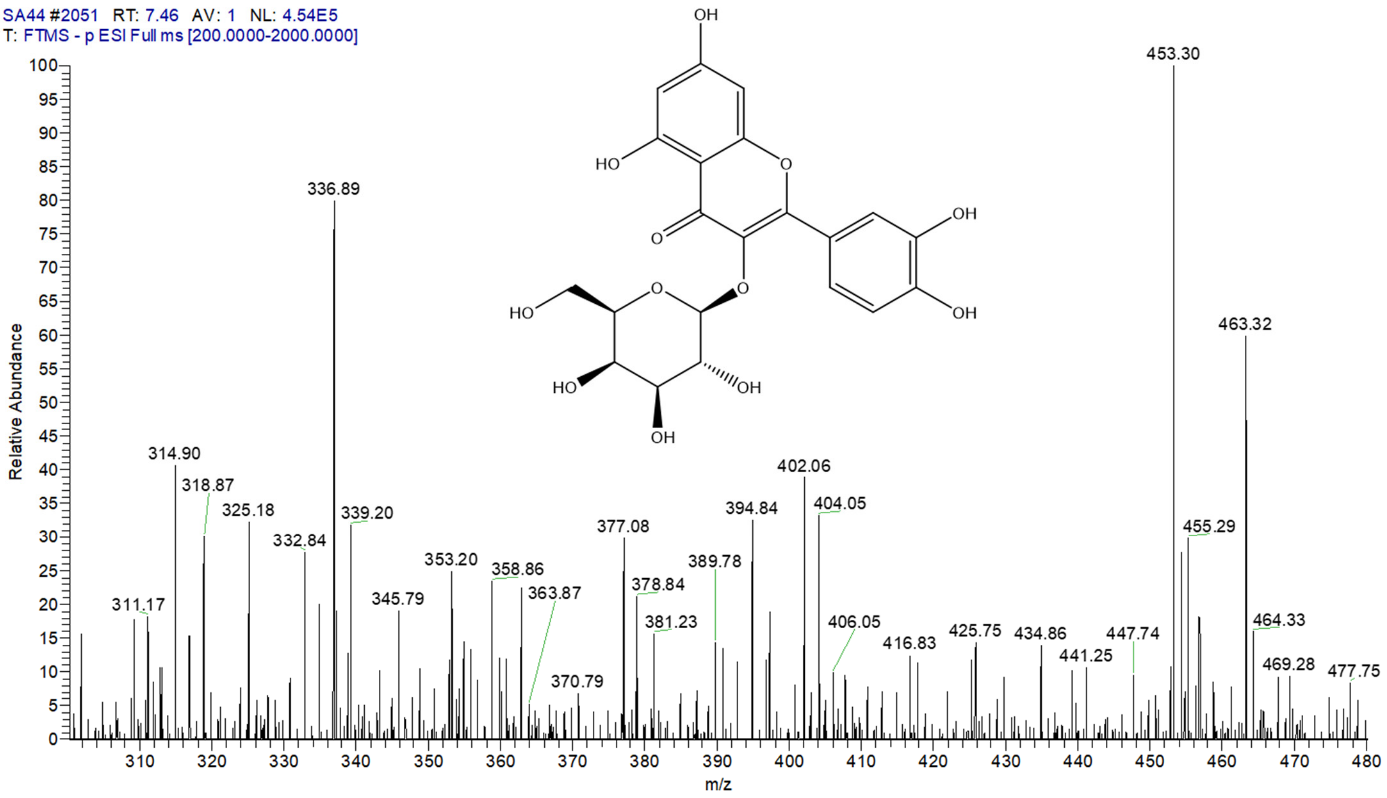

Figure S22. UHPLC–Q/Orbitrap/MS HRMS spectrum showing fragments of hyperoside (**16**).

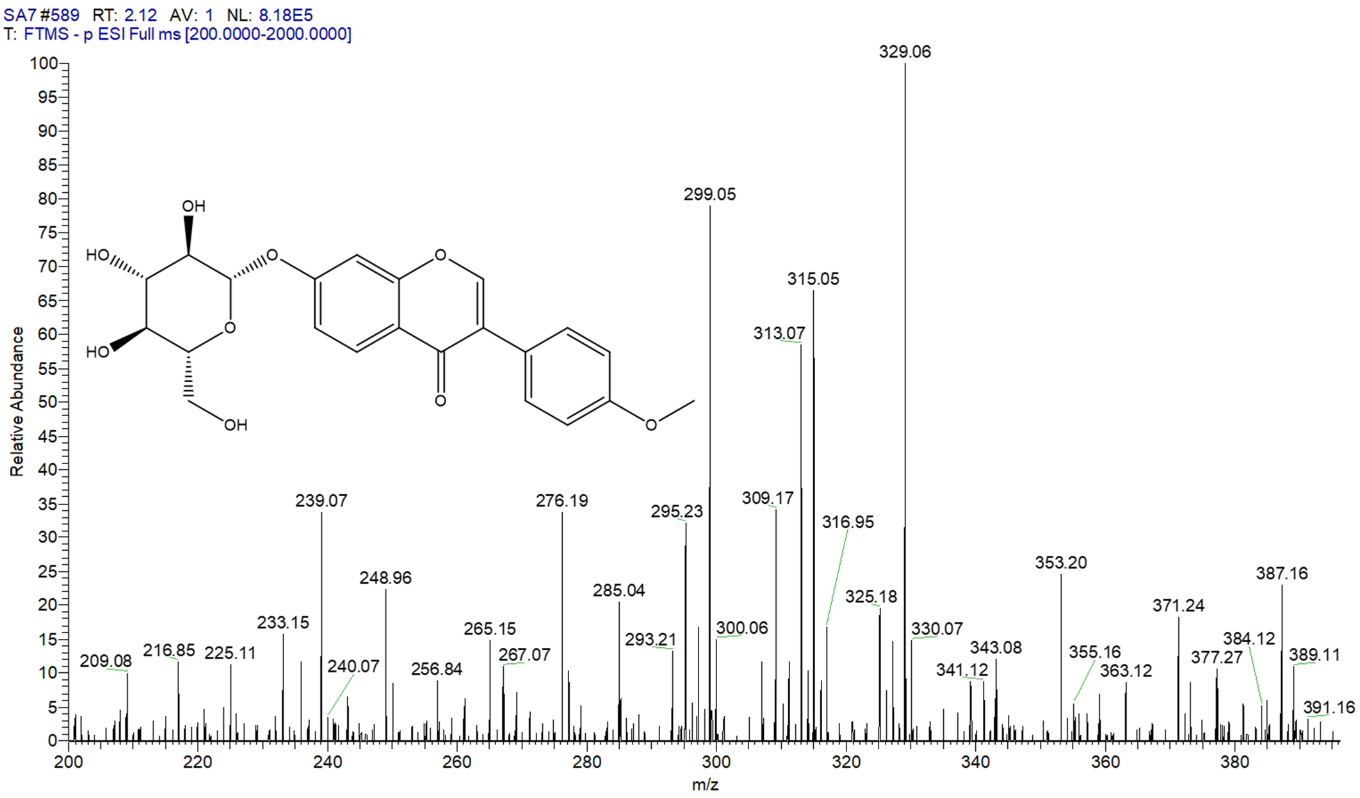

Figure S23. UHPLC–Q/Orbitrap/MS HRMS spectrum showing fragments of formononetin-7-*O*-glucoside (Ononin) (**17**).

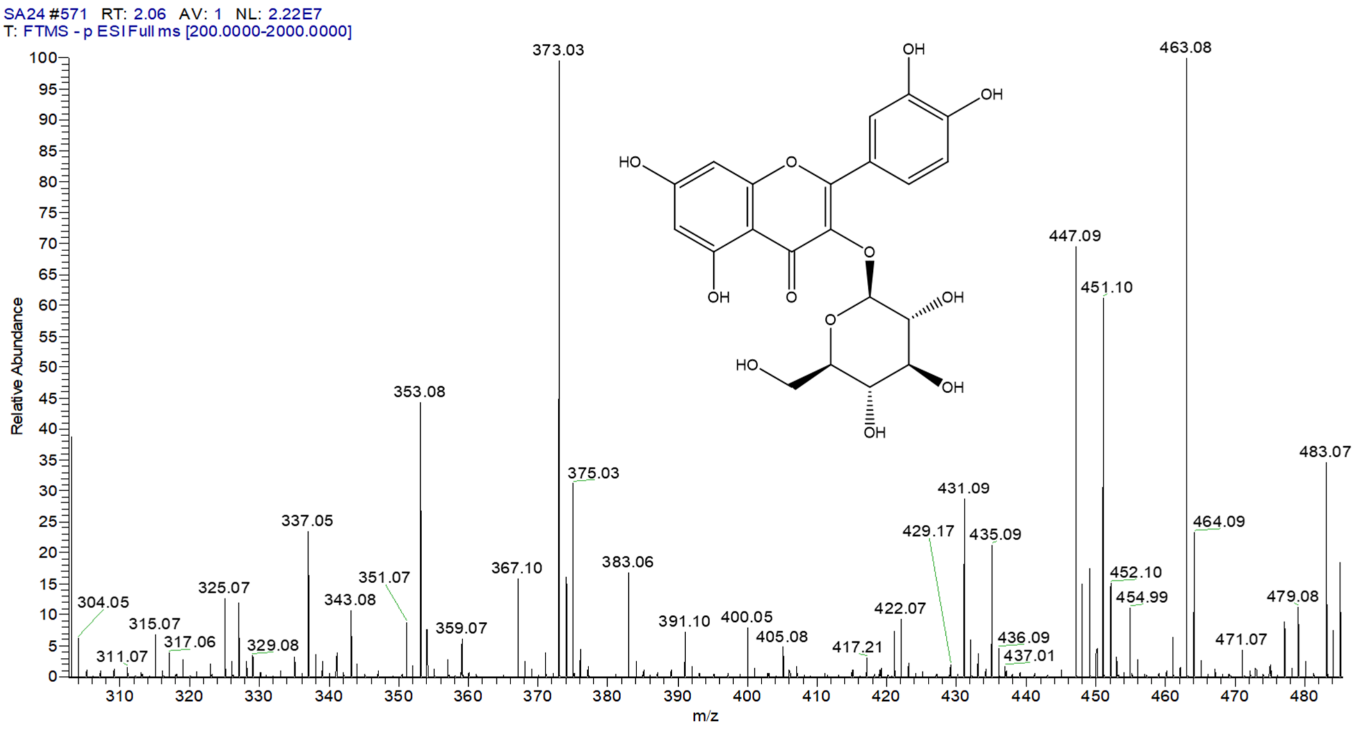

Figure S24. UHPLC–Q/Orbitrap/MS HRMS spectrum showing fragments of quercetin-3-*O*-glucoside (Isoquercitrin) (**18**).

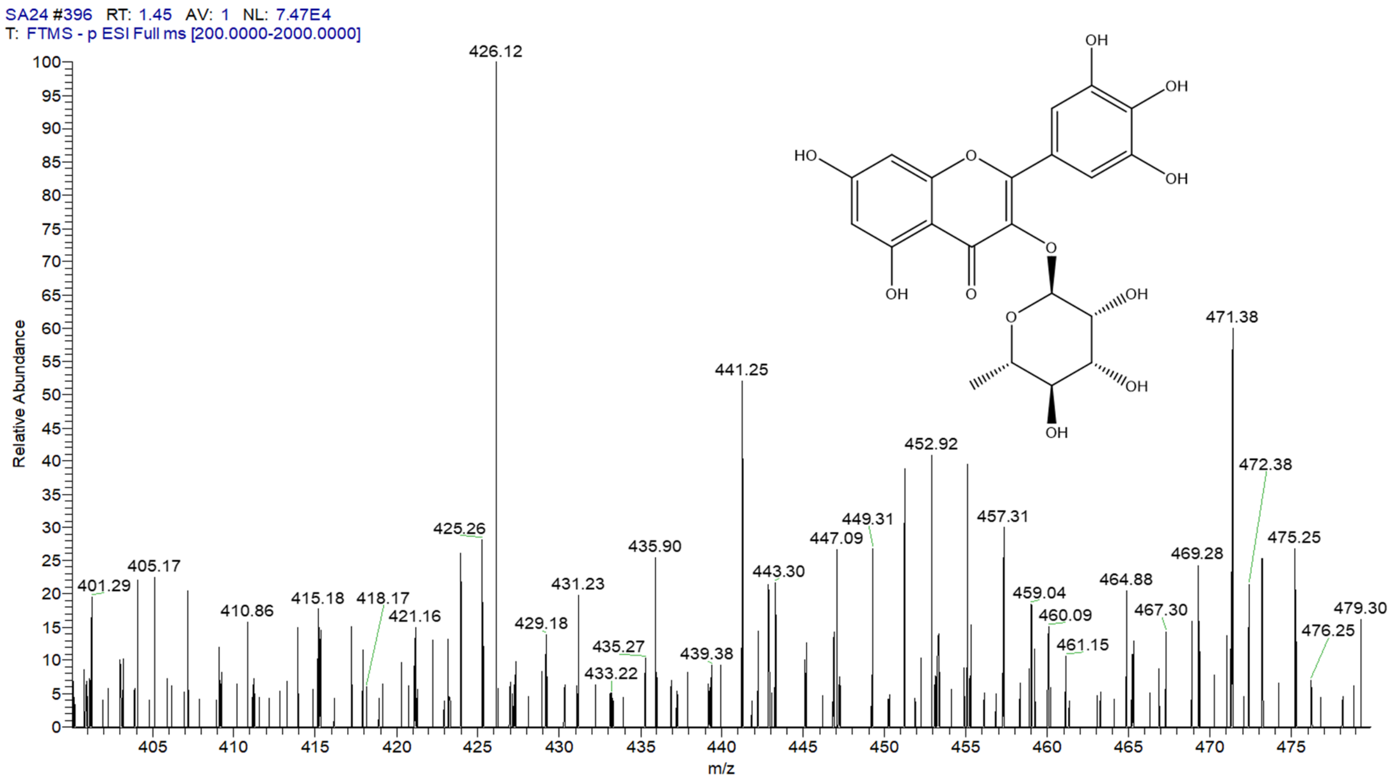

Figure S25. UHPLC–Q/Orbitrap/MS HRMS spectrum showing fragments of myricitrin (**19**).

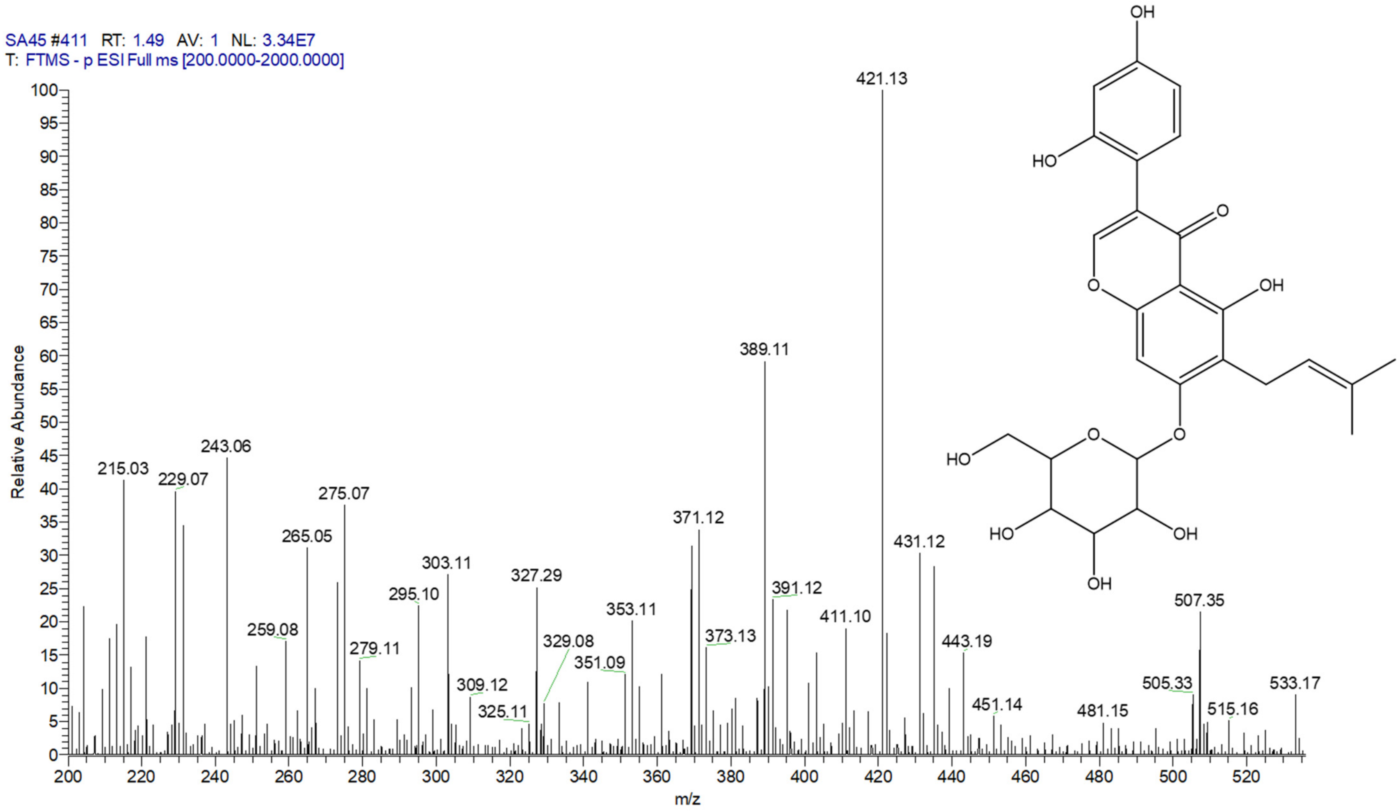

Figure S26. UHPLC–Q/Orbitrap/MS HRMS spectrum showing fragments of luteone 7-glucoside (**20**).

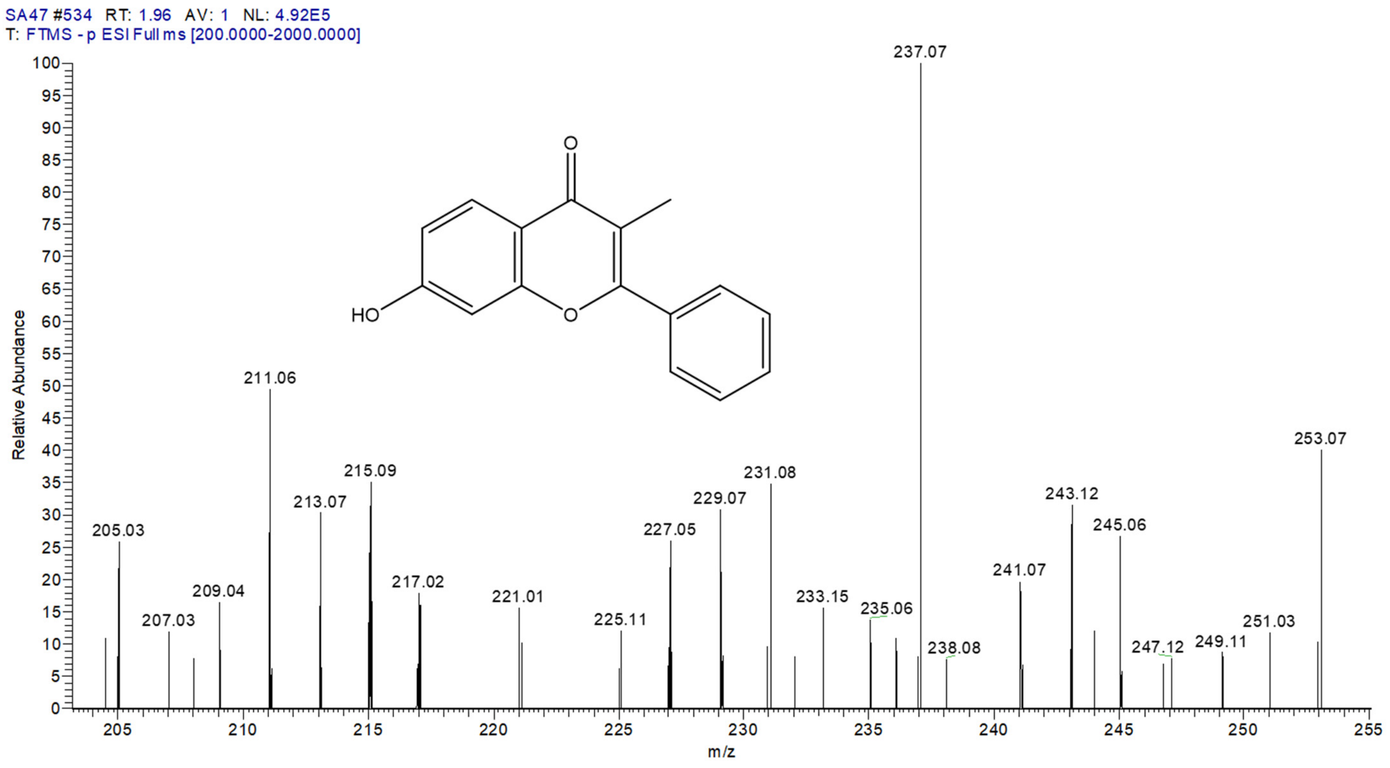

Figure S27. UHPLC–Q/Orbitrap/MS HRMS spectrum showing fragments of 7-hydroxy-3-methylflavone (**21**).

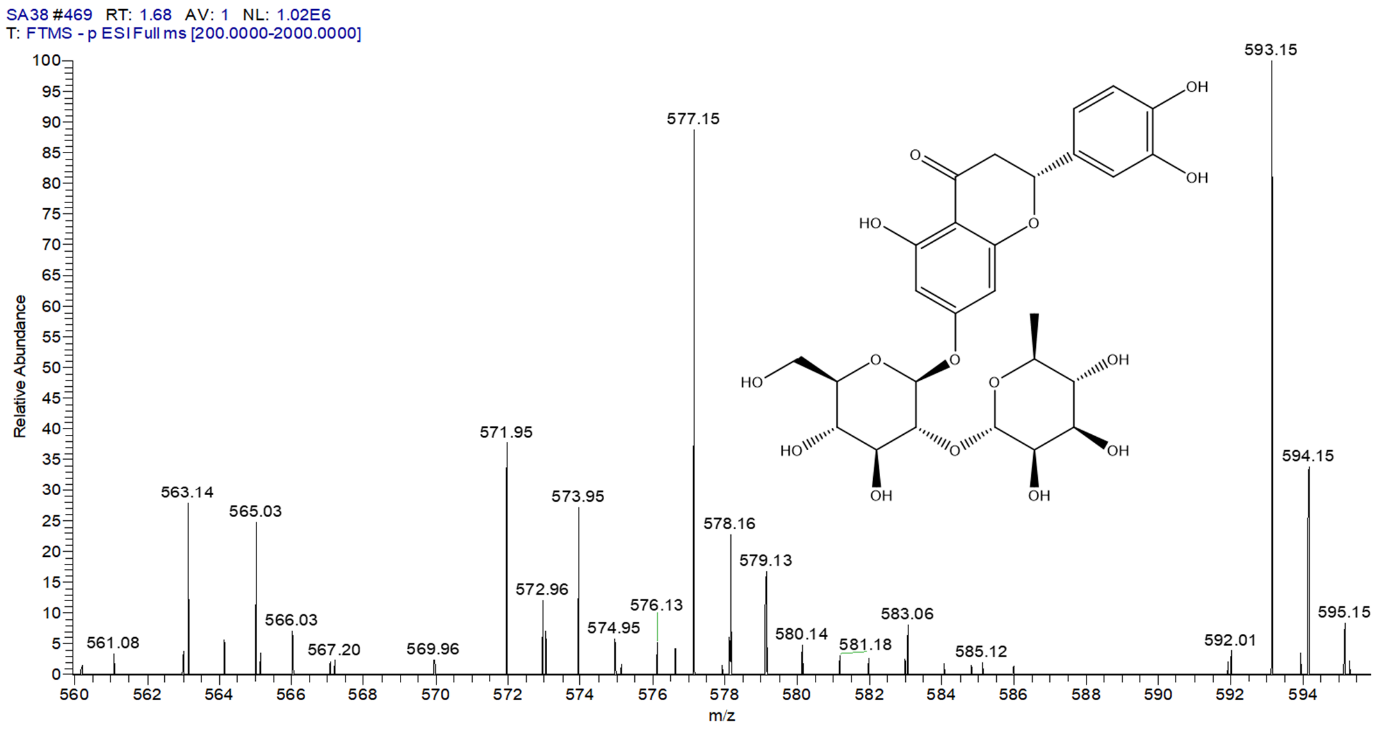

Figure S28. UHPLC–Q/Orbitrap/MS HRMS spectrum showing fragments of eriodictyol-7-*O*-neohesperidoside (Neoeriocitrin) (**22**).

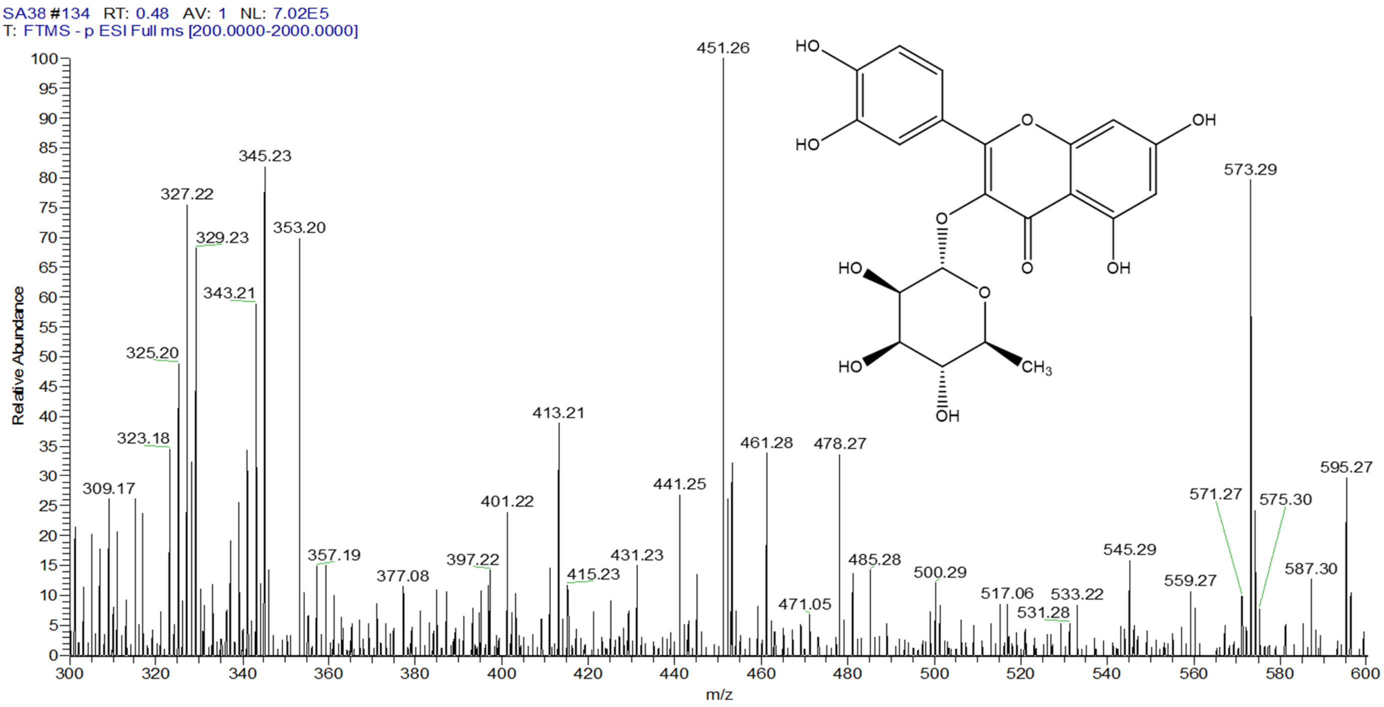

Figure S29. UHPLC–Q/Orbitrap/MS HRMS spectrum showing fragments of quercetin-3-arabinoglucoside (Peltatoside) (**23**).

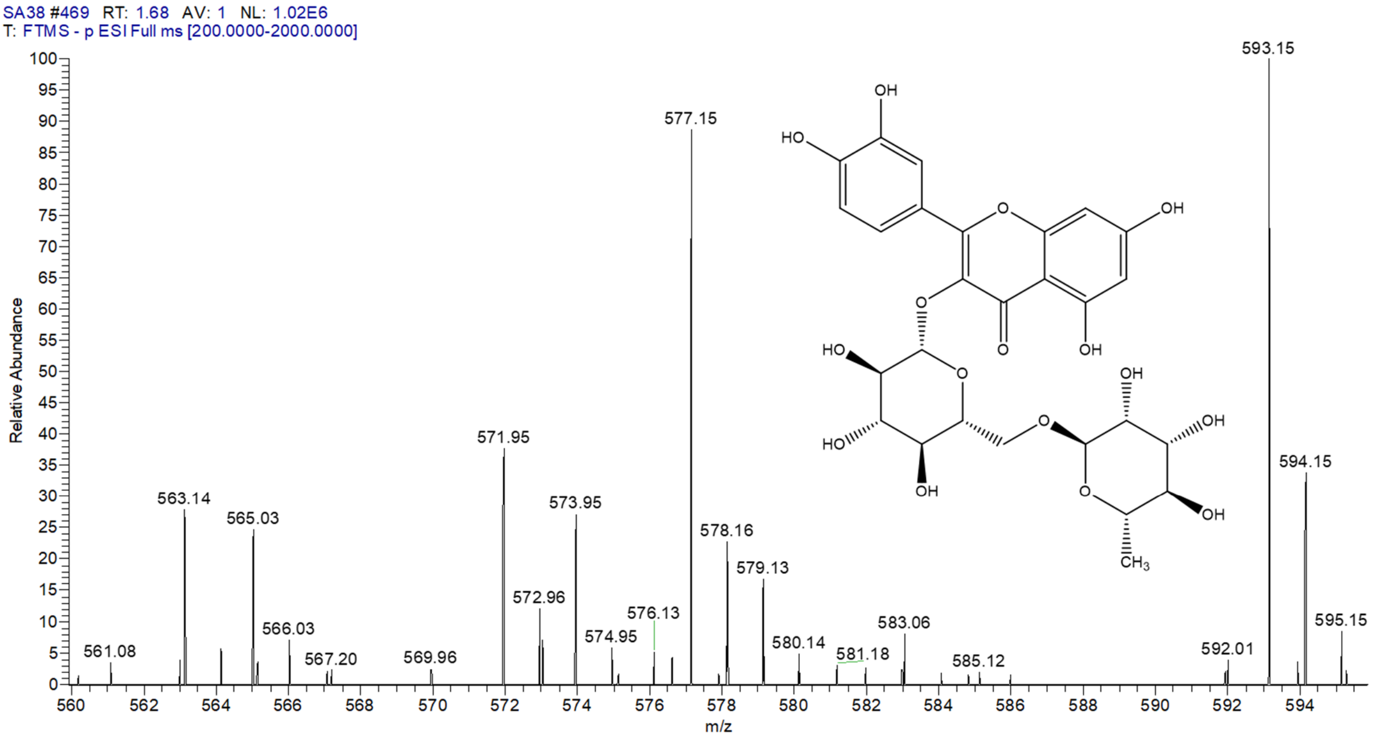

Figure S30. UHPLC–Q/Orbitrap/MS HRMS spectrum showing fragments of quercetin-3-*O*-vicianoside (**24**).

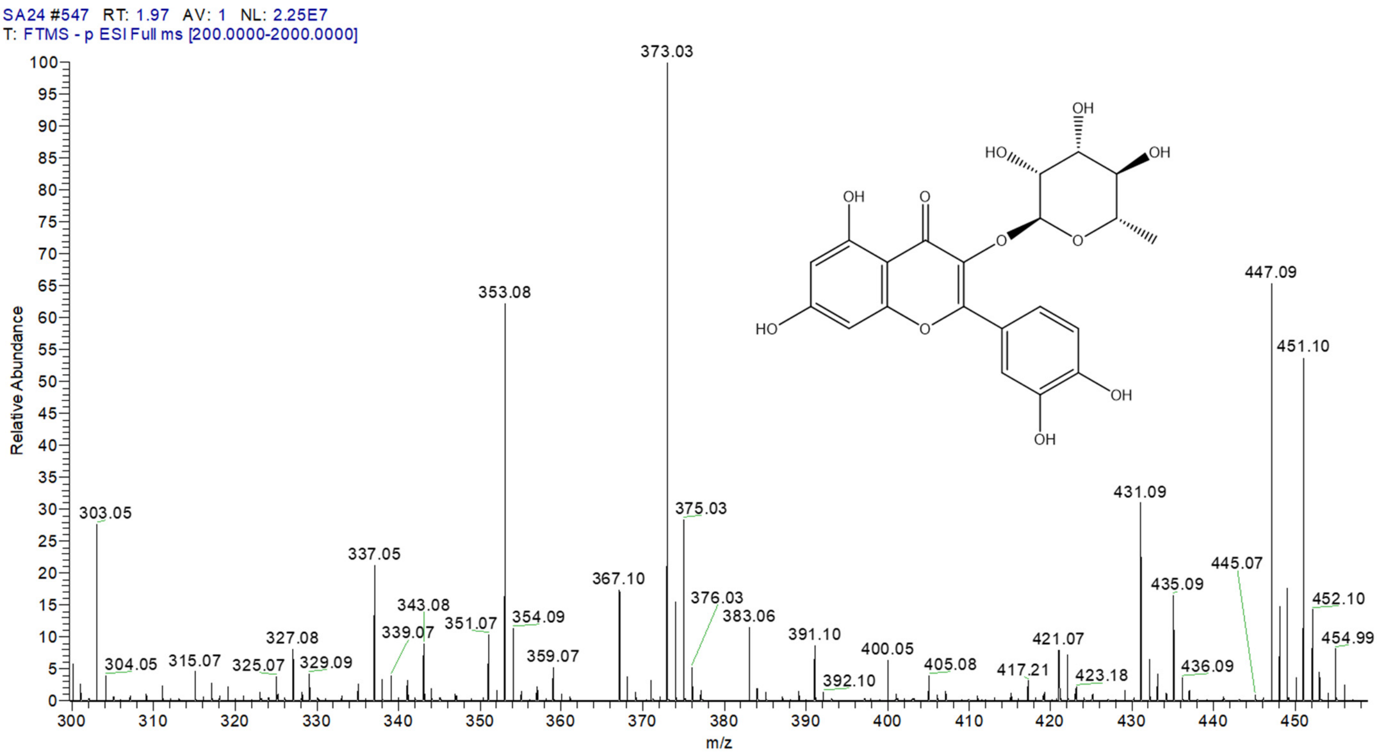

Figure S31. UHPLC–Q/Orbitrap/MS HRMS spectrum showing fragments of quercitrin (**25**).

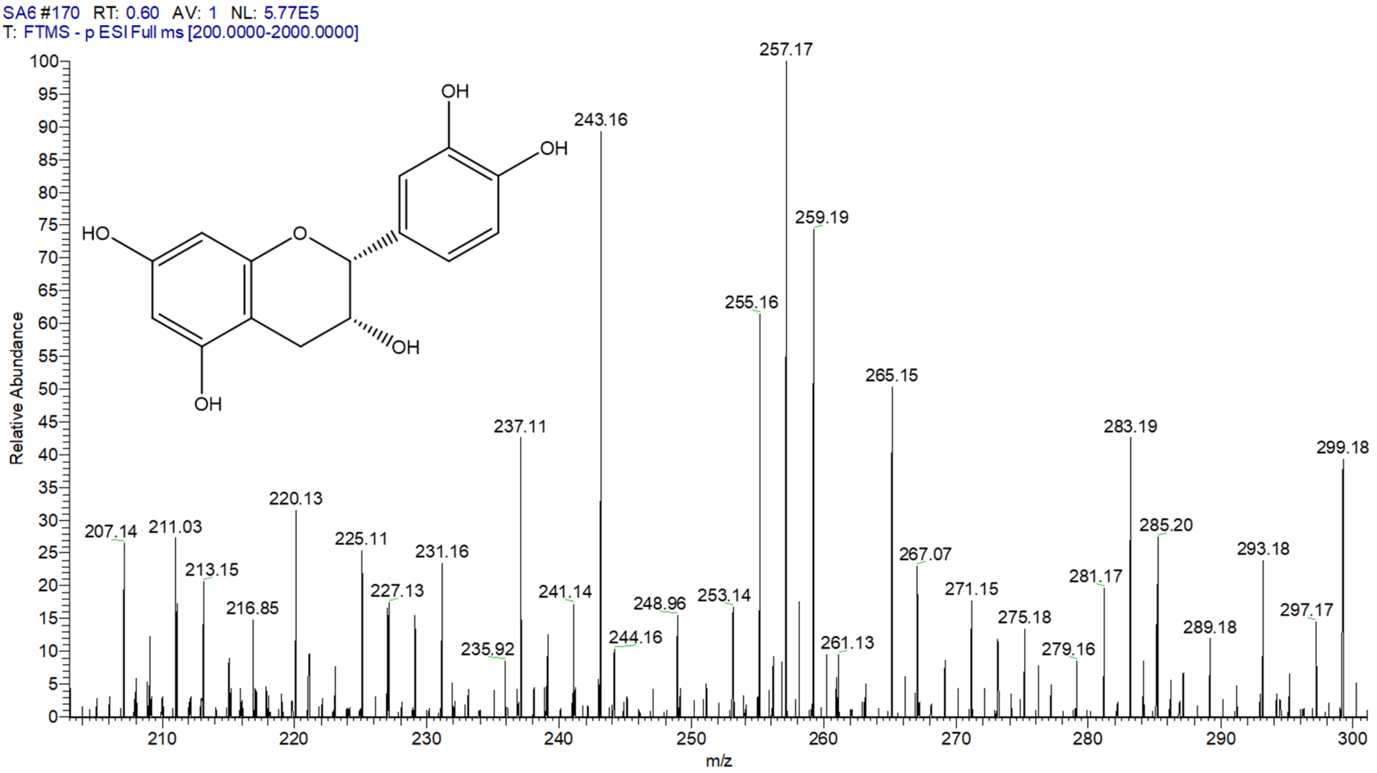

Figure S32. UHPLC–Q/Orbitrap/MS HRMS spectrum showing fragments of epicatechin (**26**).

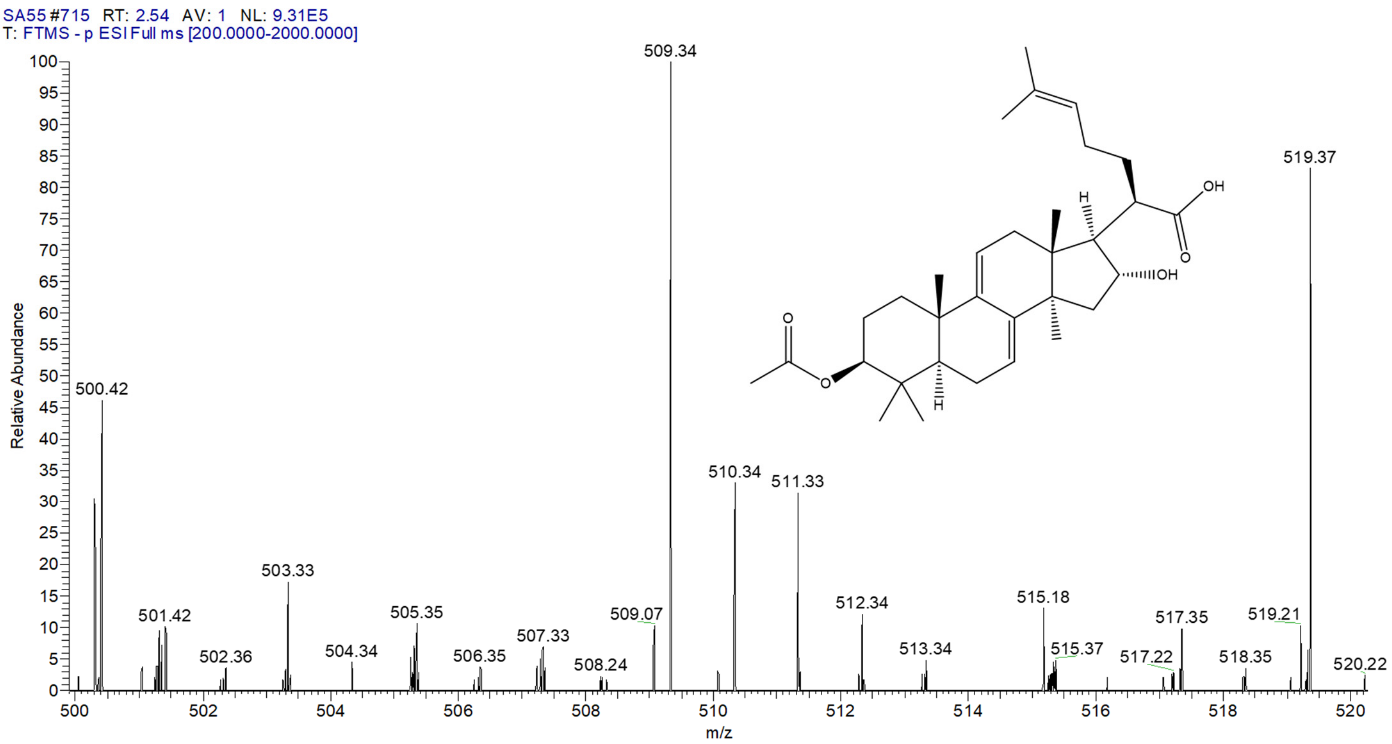

Figure S33. UHPLC–Q/Orbitrap/MS HRMS spectrum showing fragments of 3-*O*-acetyl-16alpha-hydroxydehydrotrametenolic acid (**27**).

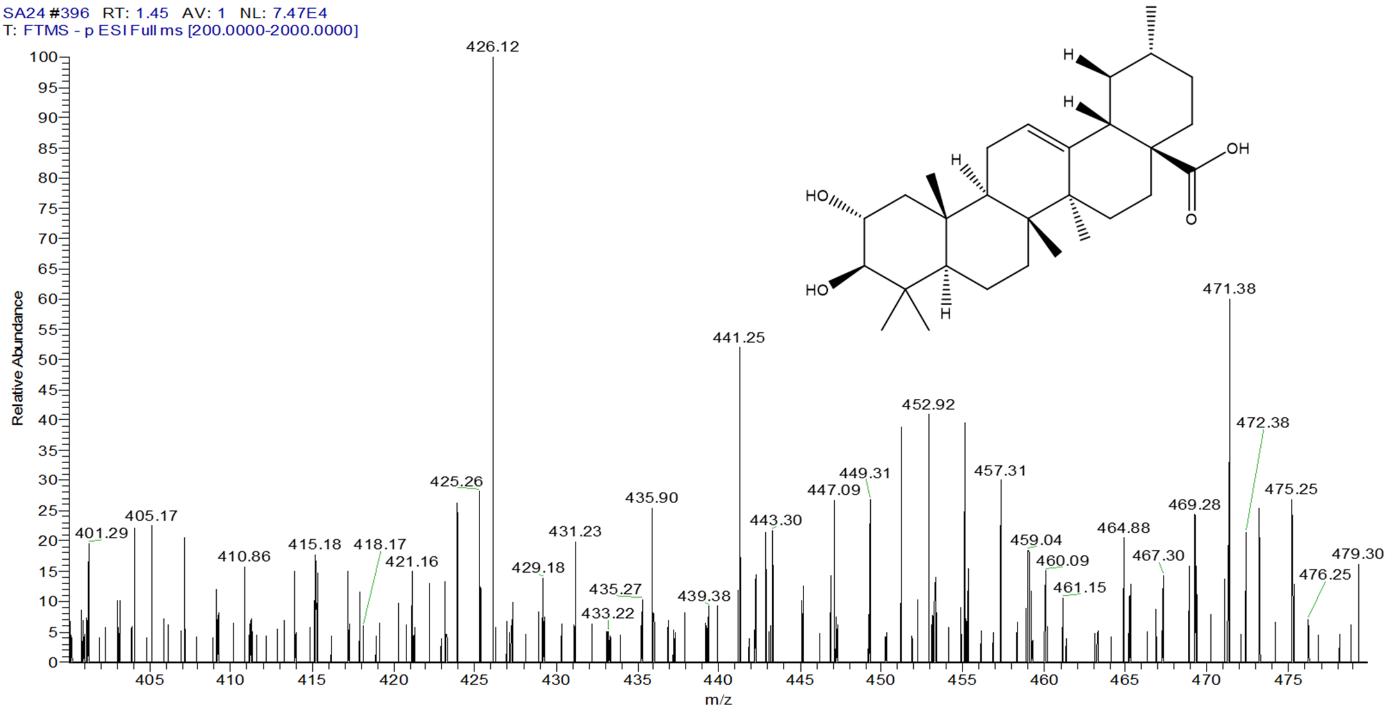

Figure S34. UHPLC–Q/Orbitrap/MS HRMS spectrum showing fragments of corosolic acid (**28**).

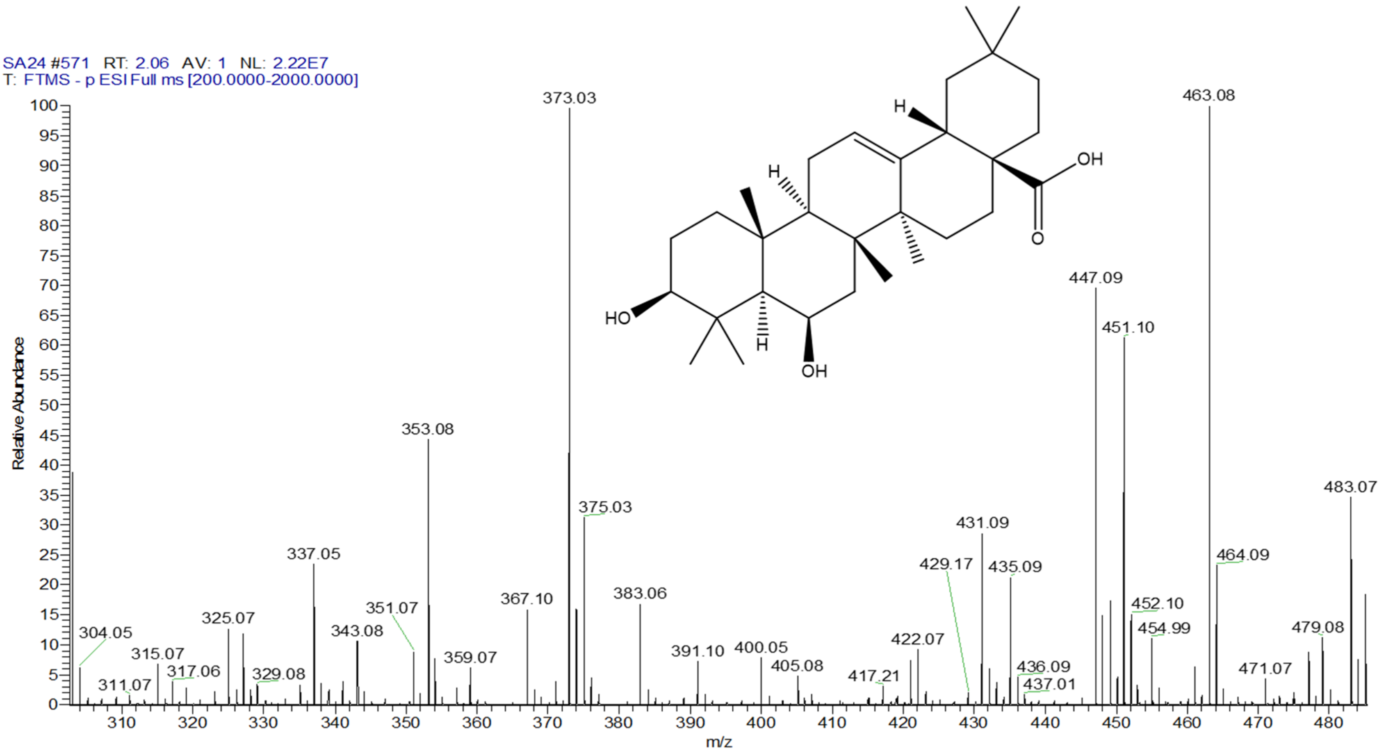

Figure S35. UHPLC–Q/Orbitrap/MS HRMS spectrum showing fragments of sumaresinolic acid (**29**).

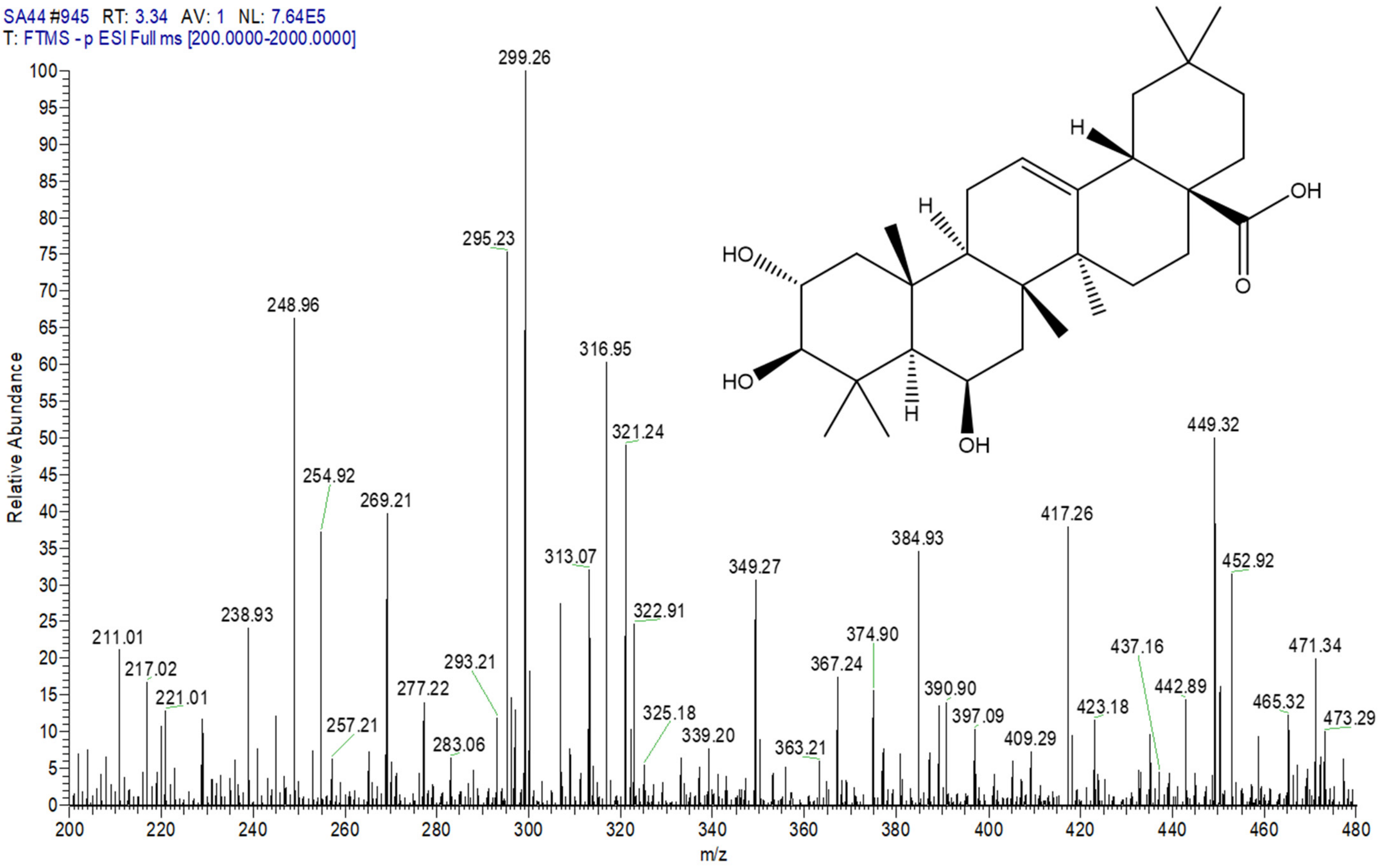

Figure S36. UHPLC–Q/Orbitrap/MS HRMS spectrum showing fragments of maslinic acid (**30**).

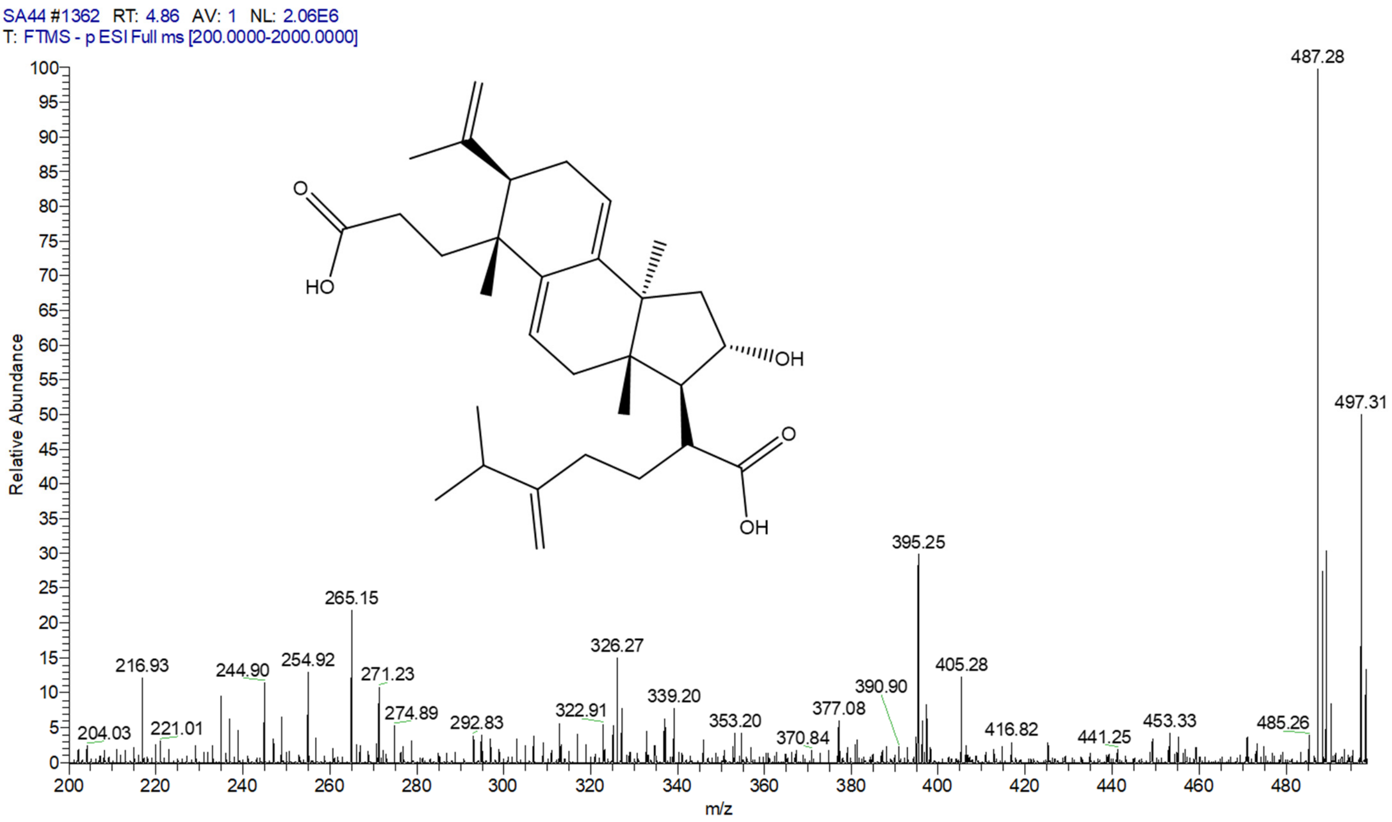

Figure S37. UHPLC–Q/Orbitrap/MS HRMS spectrum showing fragments of poricoic acid A (**31**).

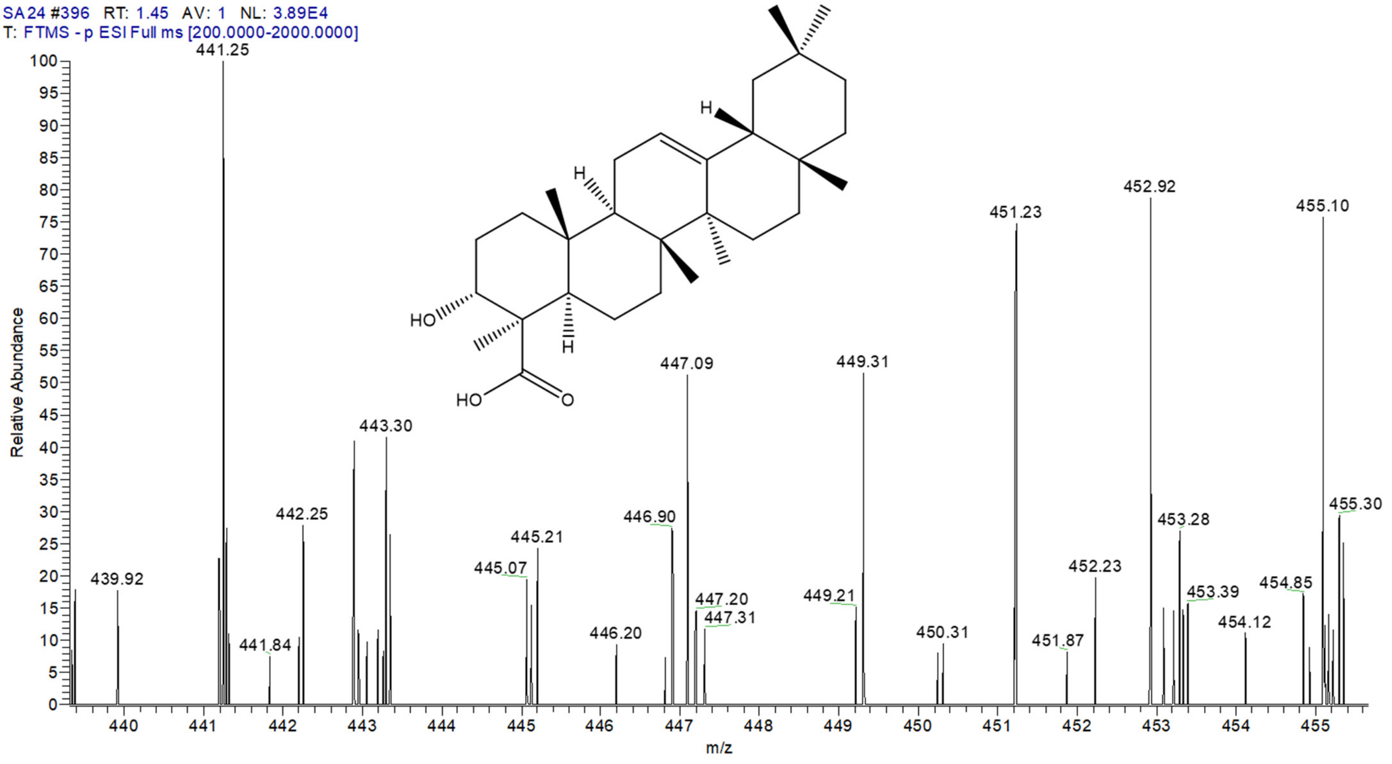

Figure S38. UHPLC–Q/Orbitrap/MS HRMS spectrum showing fragments of alpha-boswellic acid (**32**).

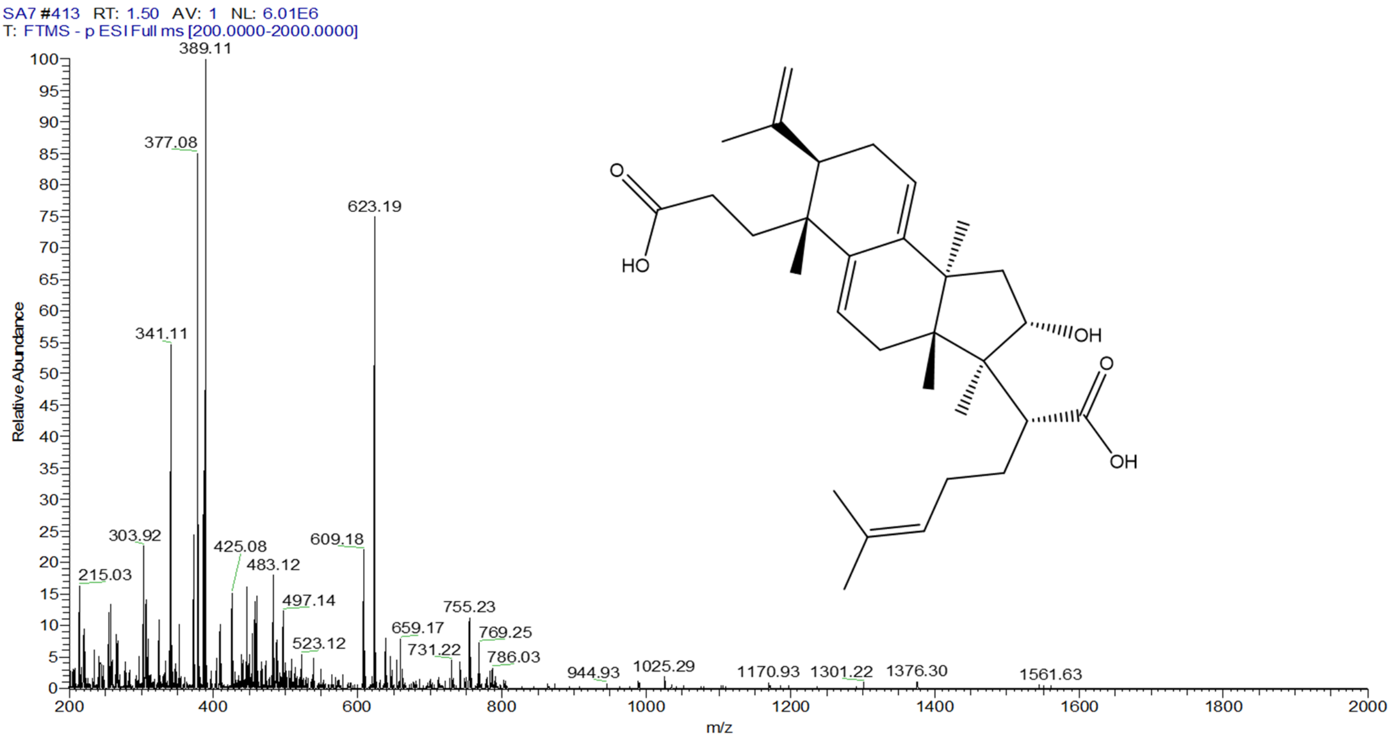

Figure S39. UHPLC–Q/Orbitrap/MS HRMS spectrum showing fragments of poricoic acid B (**33**).

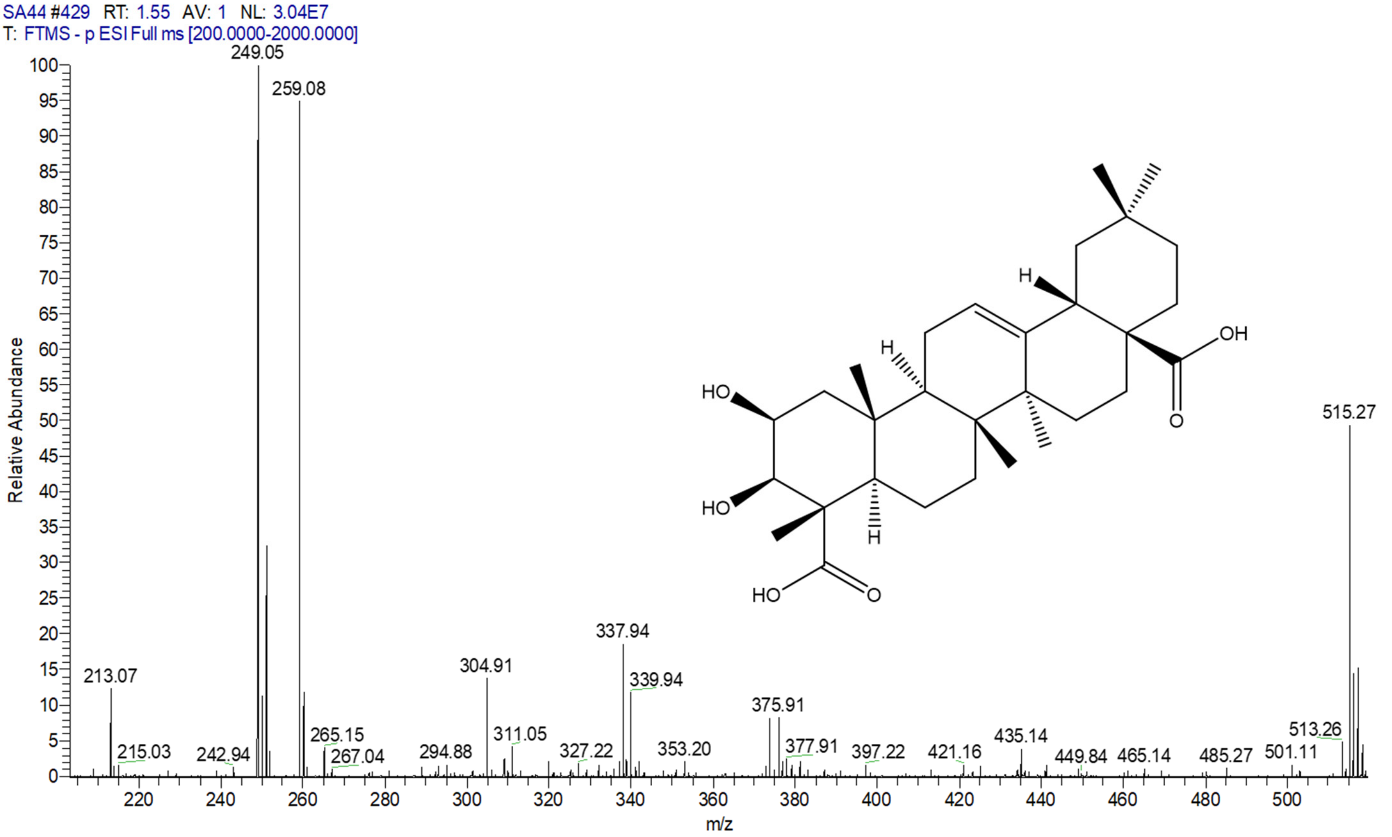

Figure S40. UHPLC–Q/Orbitrap/MS HRMS spectrum showing fragments of medicagenic acid (**34**).

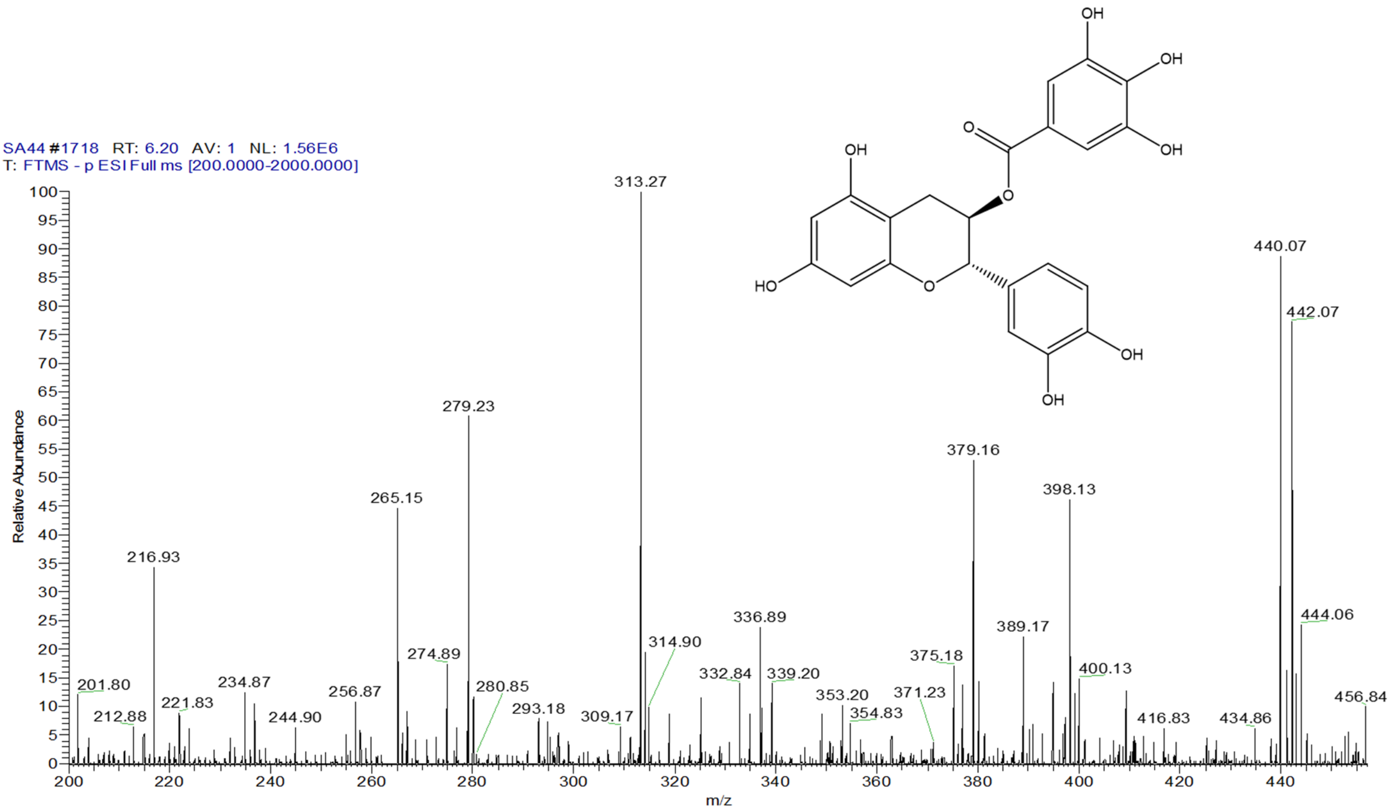

Figure S41. UHPLC–Q/Orbitrap/MS HRMS spectrum showing fragments of catechin gallate (**35**).

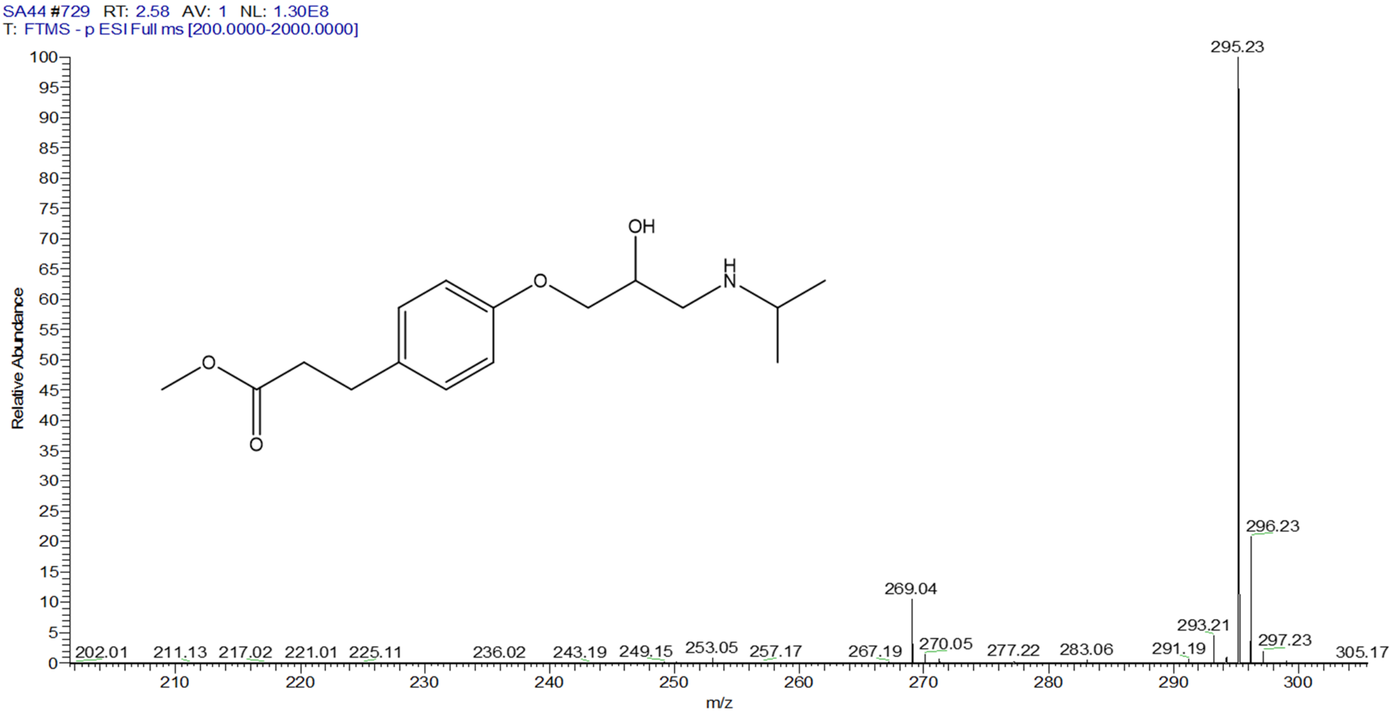

Figure S42. UHPLC–Q/Orbitrap/MS HRMS spectrum showing fragments of esmolol (**36**).

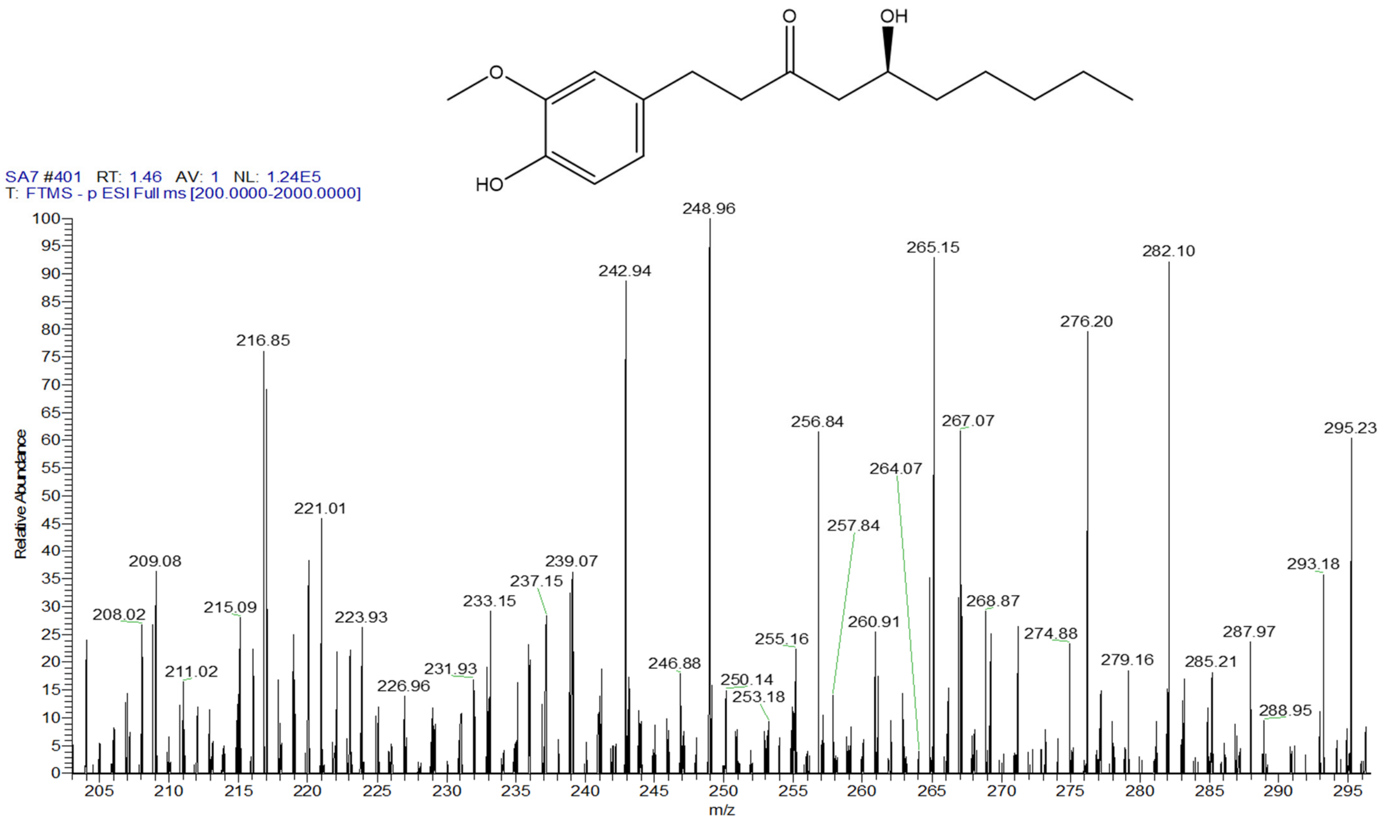

Figure S43. UHPLC–Q/Orbitrap/MS HRMS spectrum showing fragments of 6-gingerol (**37**).

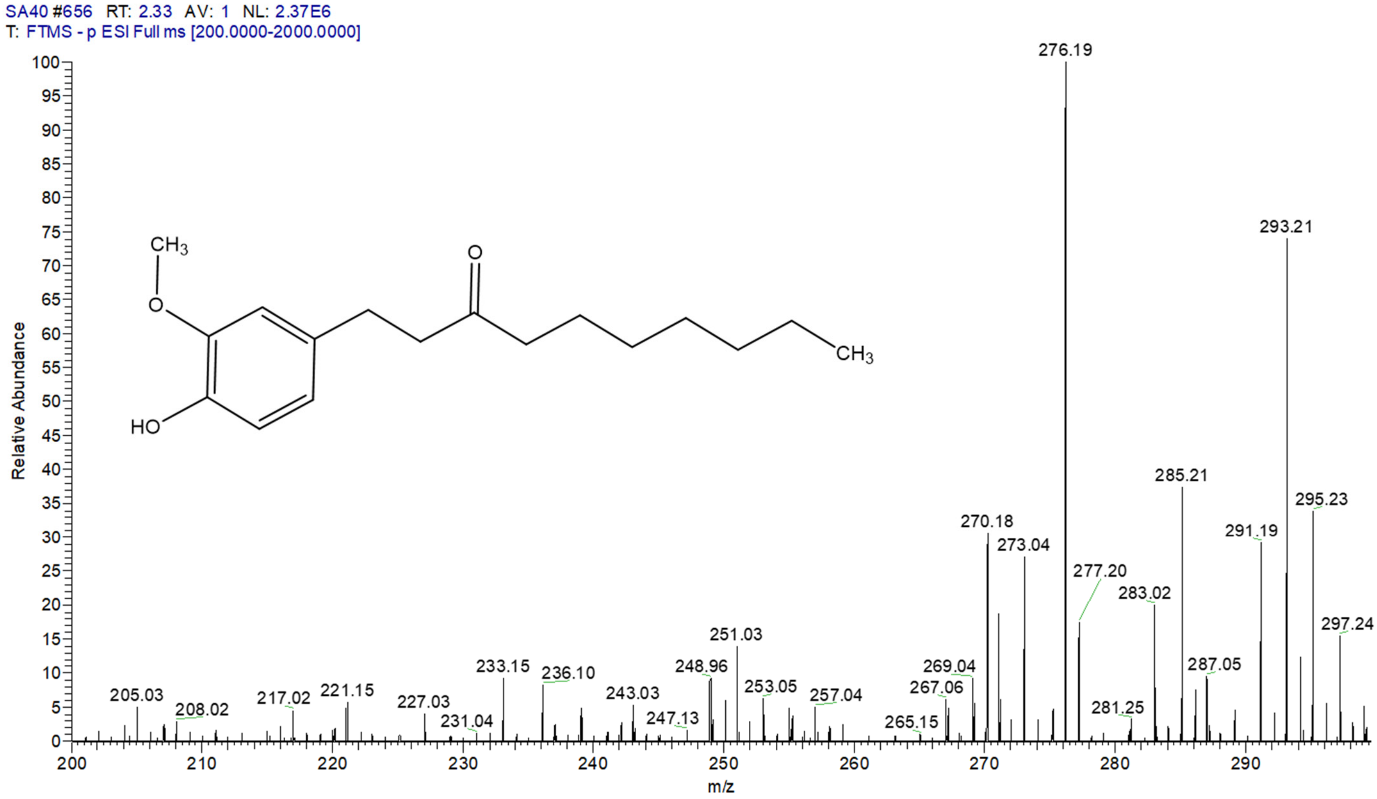

Figure S44. UHPLC–Q/Orbitrap/MS HRMS spectrum showing fragments of 6-paradol (**38**).

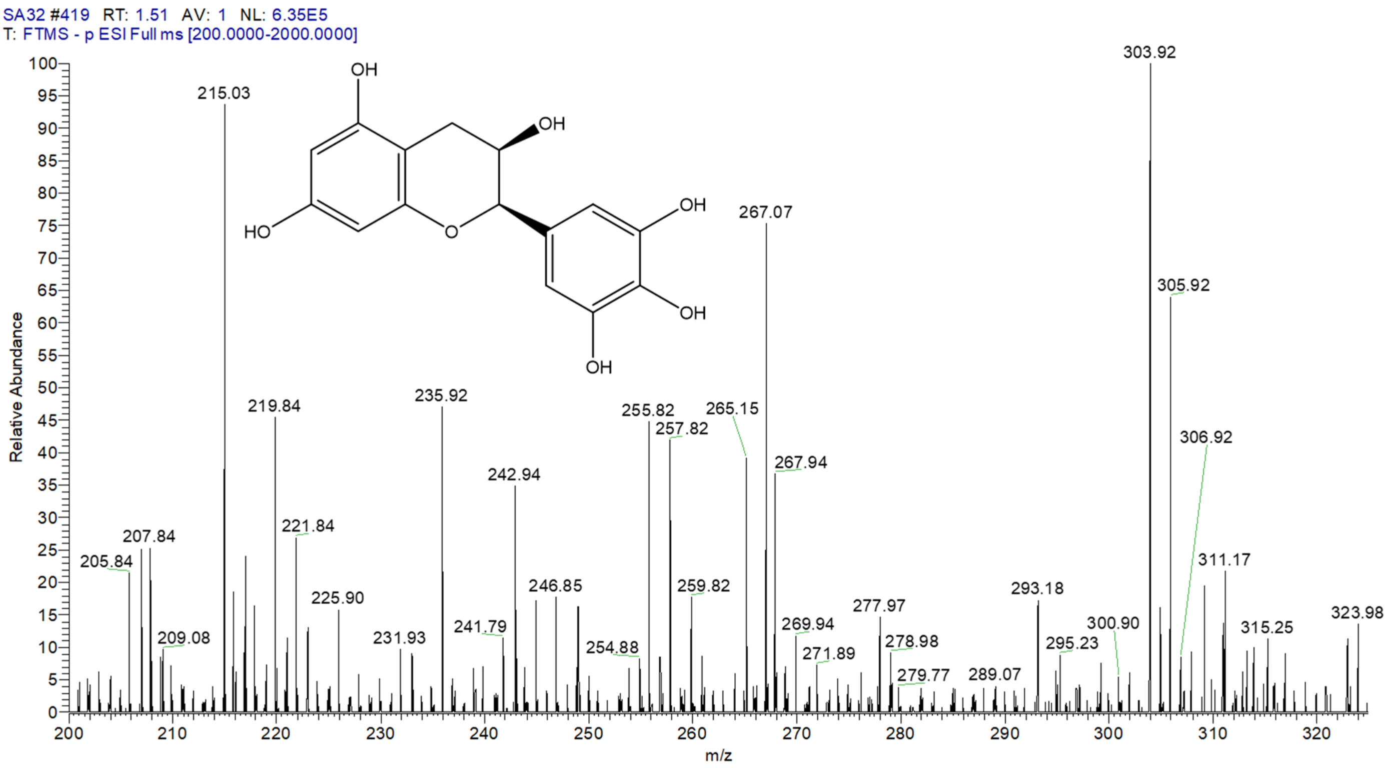

Figure S45. UHPLC–Q/Orbitrap/MS HRMS spectrum showing fragments of (−)-epigallocatechin (**39**).

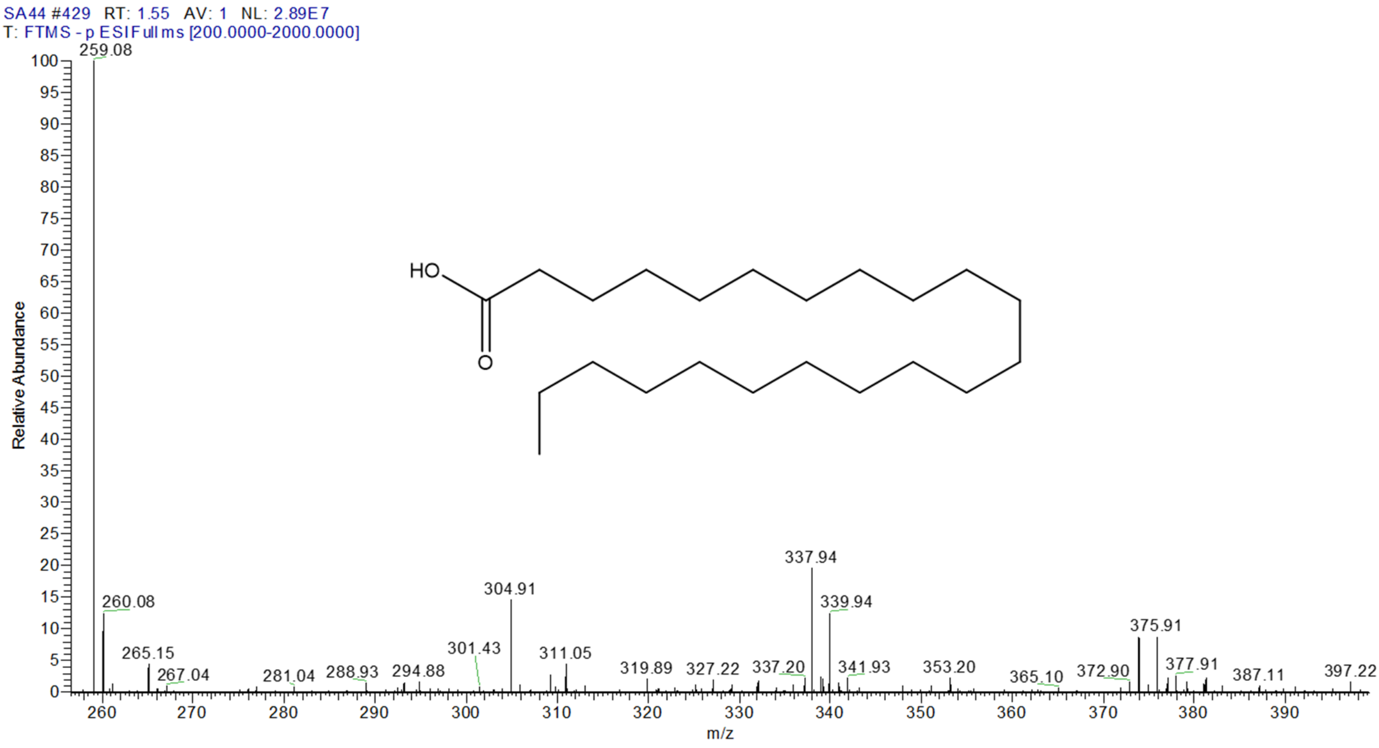

Figure S46. UHPLC–Q/Orbitrap/MS HRMS spectrum showing fragments of behenic acid (**40**).

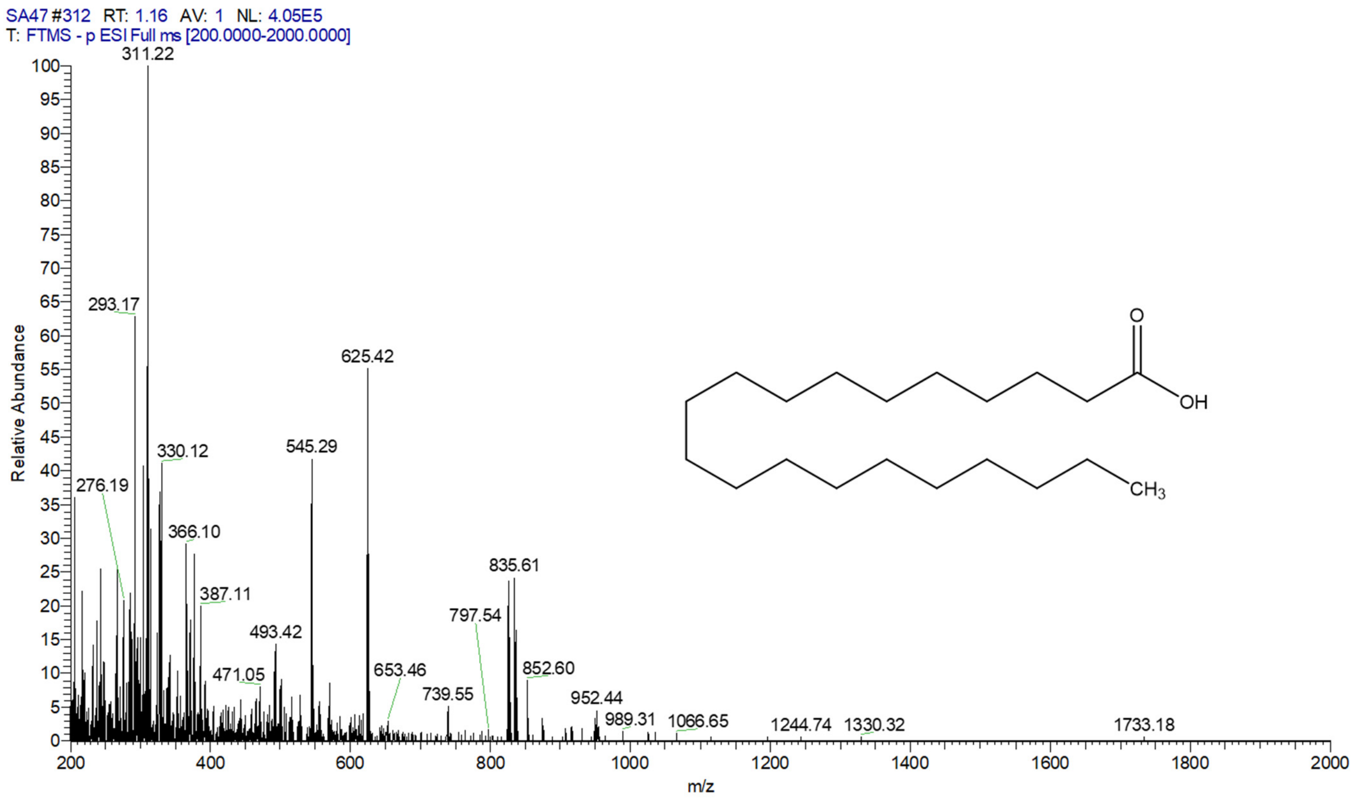

Figure S47. UHPLC–Q/Orbitrap/MS HRMS spectrum showing fragments of arachidic acid (**41**).

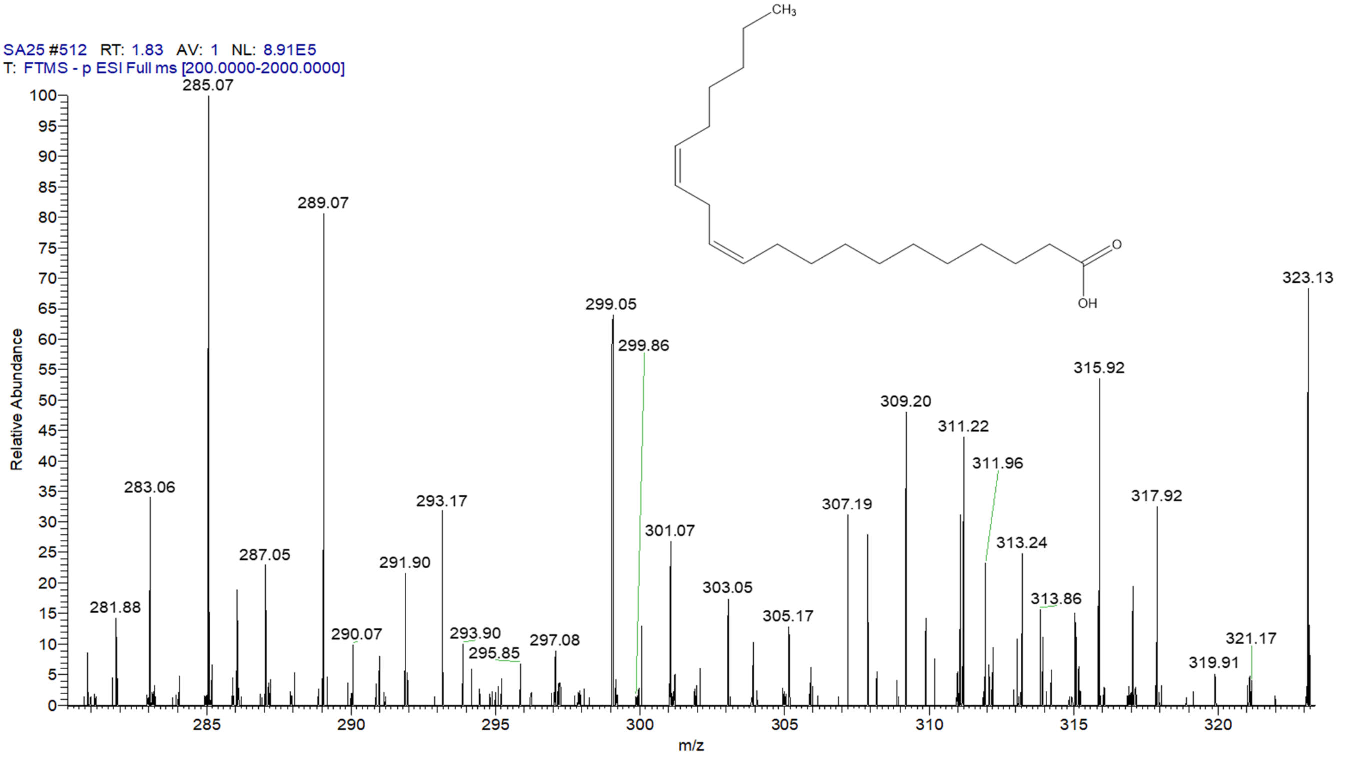

Figure S48. UHPLC–Q/Orbitrap/MS HRMS spectrum showing fragments of eicosadieneoic acid (**42**).

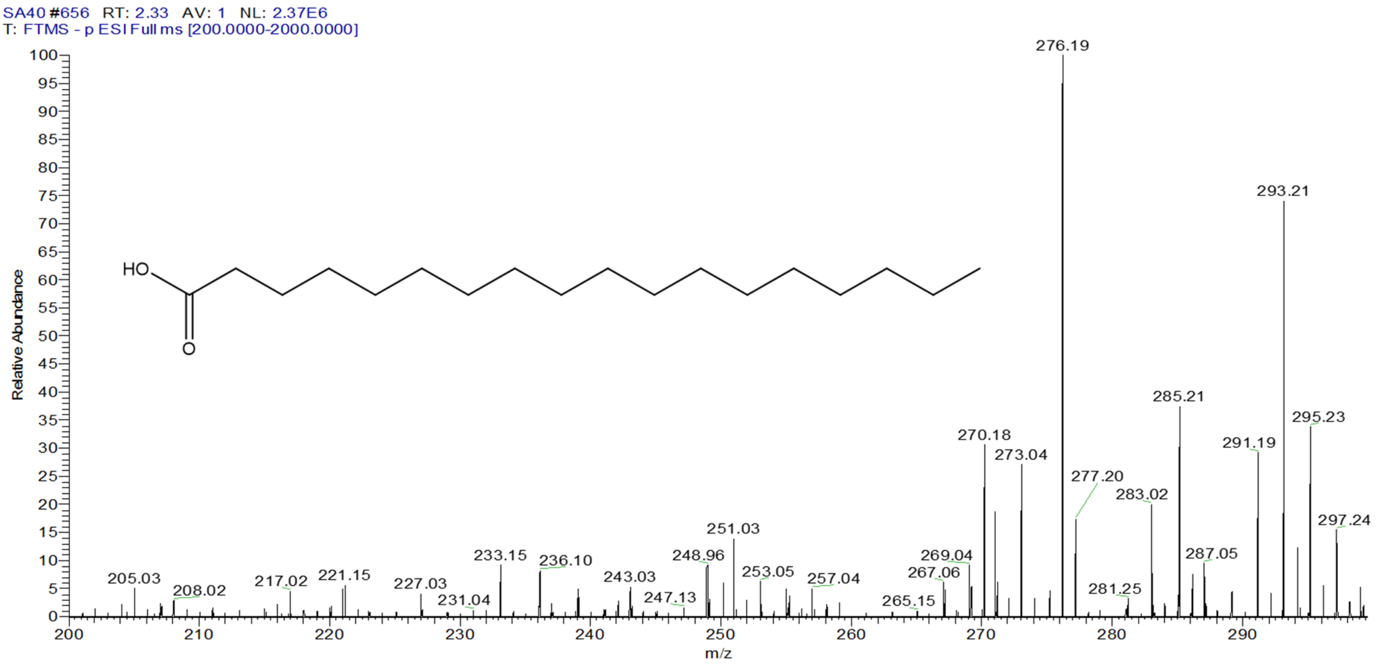

Figure S49. UHPLC–Q/Orbitrap/MS HRMS spectrum showing fragments of stearic acid (**43**).

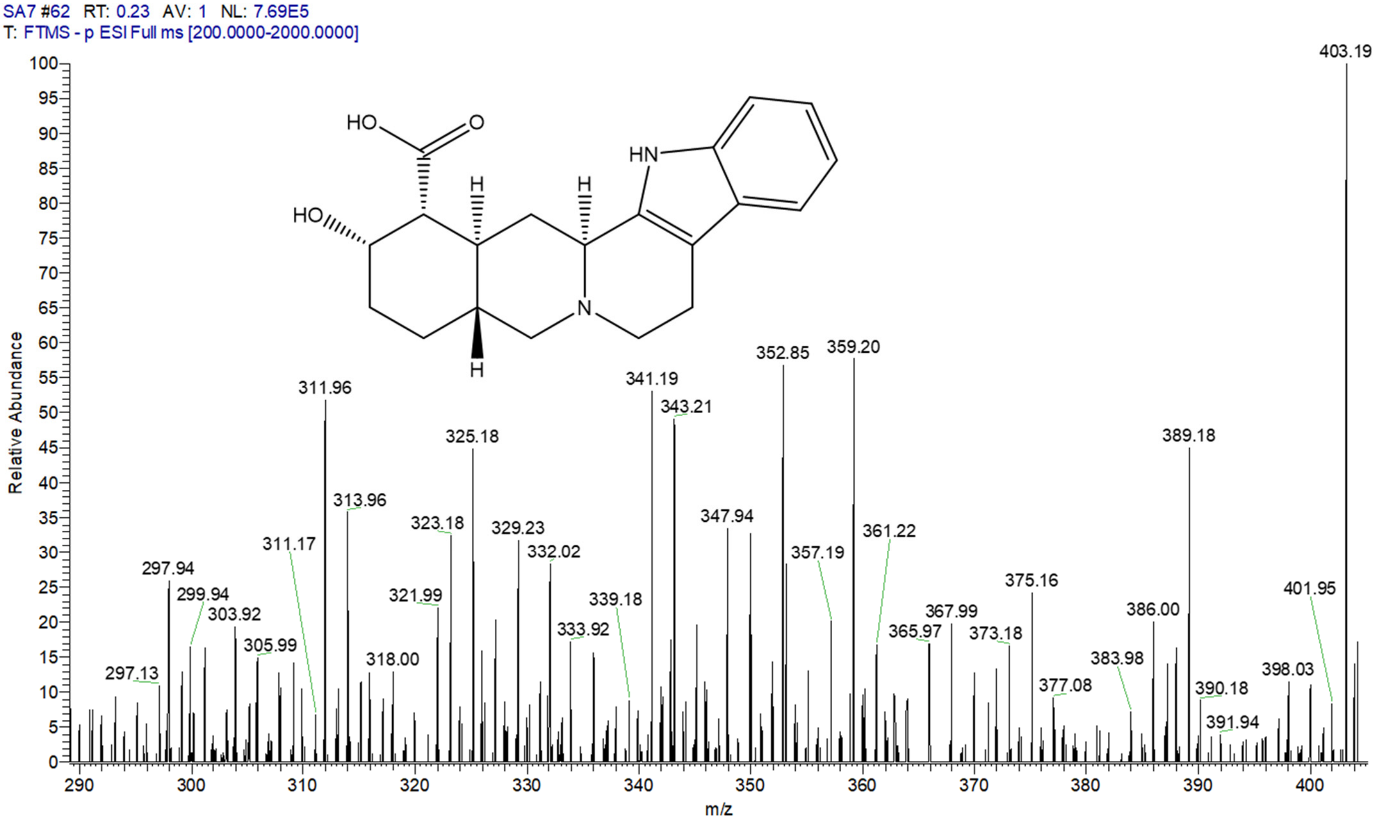

Figure S50. UHPLC–Q/Orbitrap/MS HRMS spectrum showing fragments of yohimbic acid (**44**).

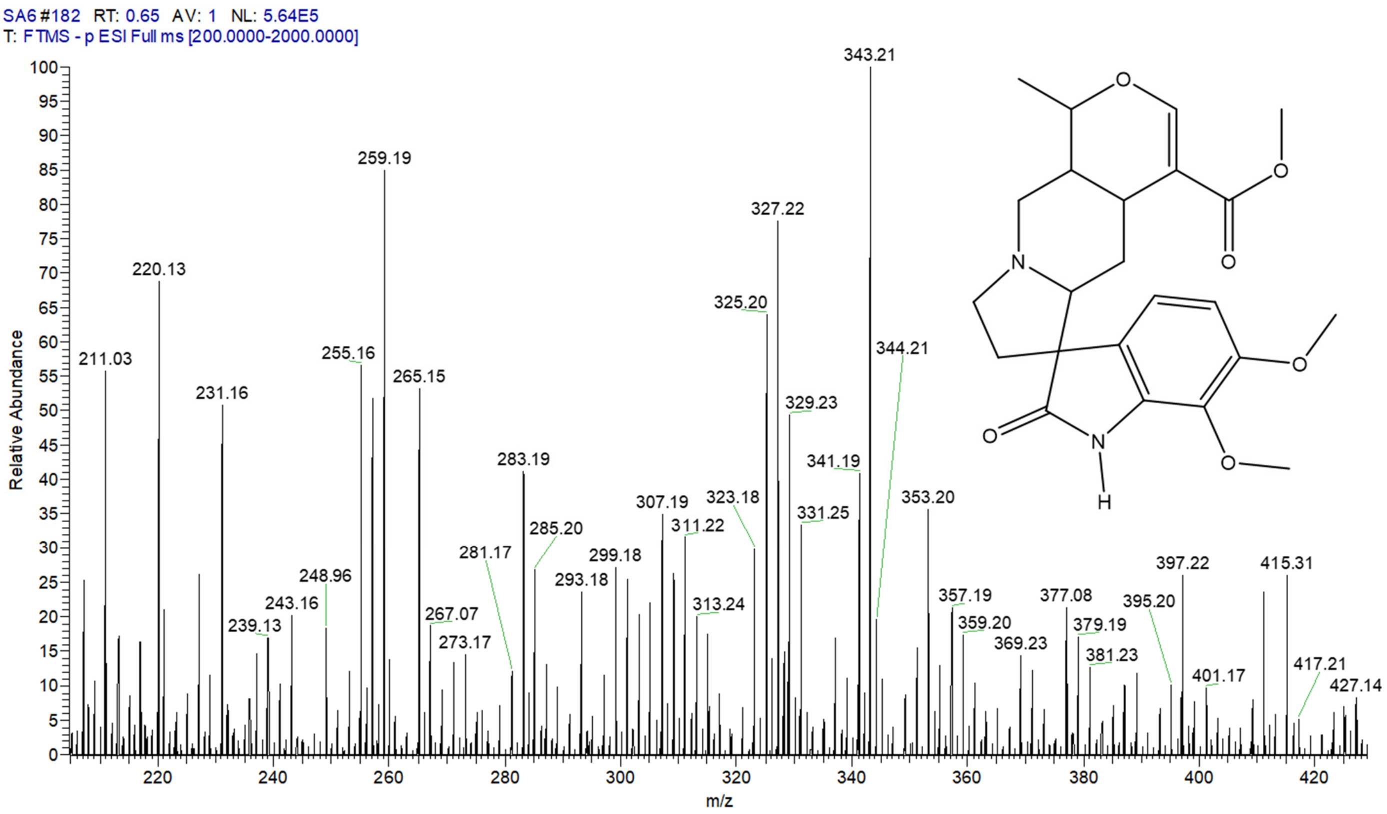

Figure S51. UHPLC–Q/Orbitrap/MS HRMS spectrum showing fragments of isomajdine (**45**).

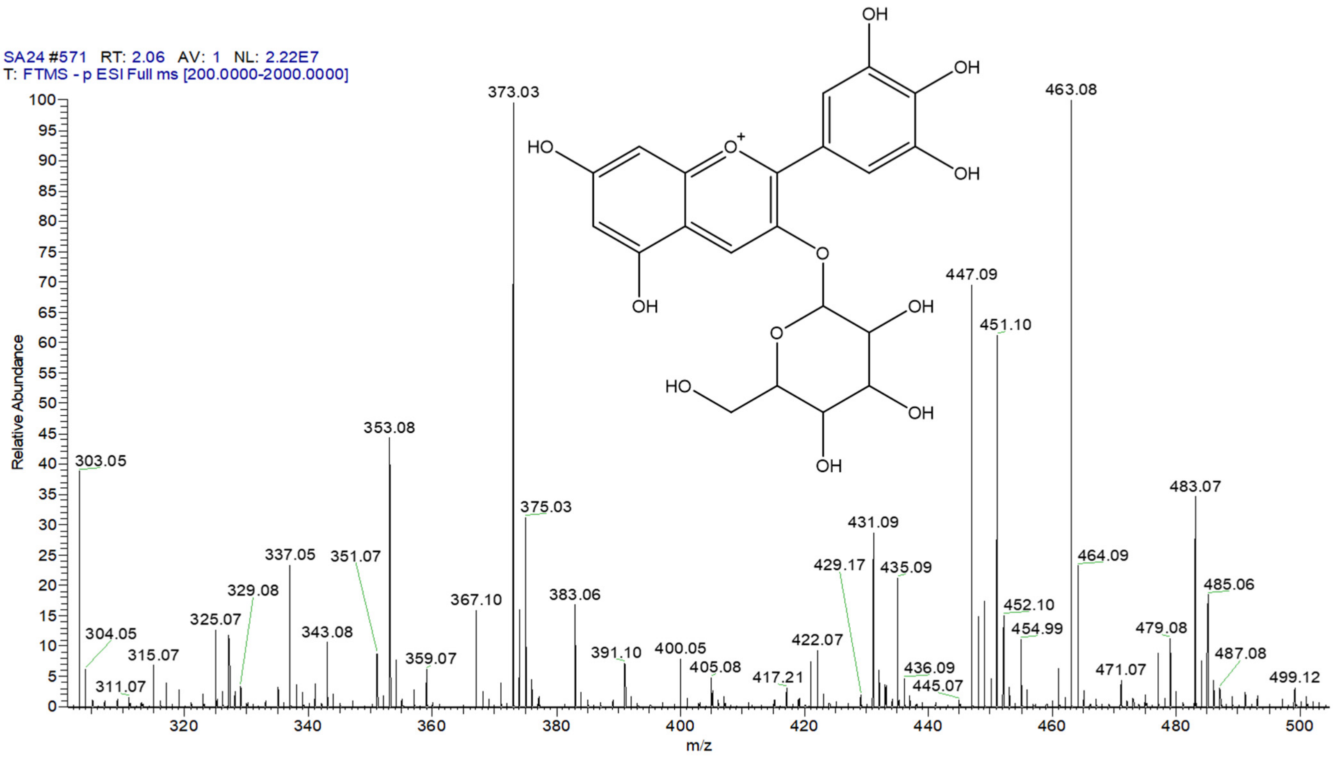

Figure S52. UHPLC–Q/Orbitrap/MS HRMS spectrum showing fragments of delphinidin 3-galactoside (**46**).

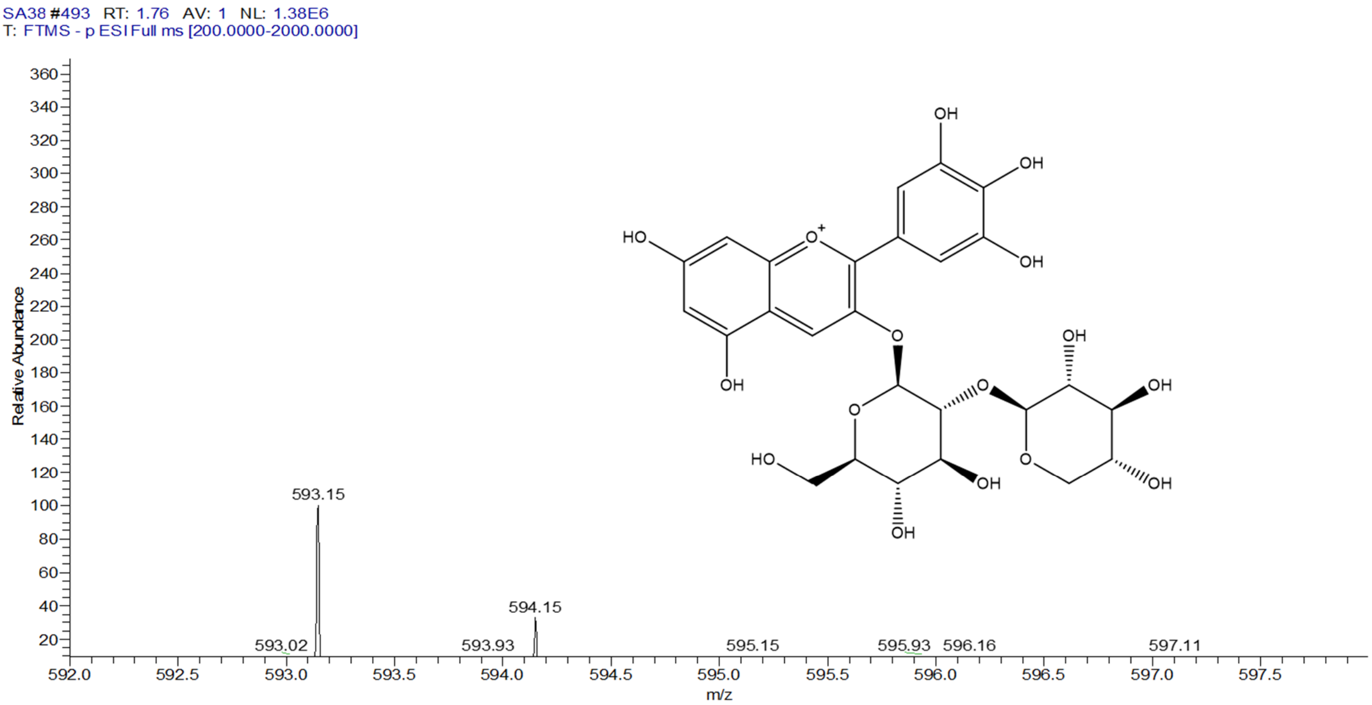

Figure S53. UHPLC–Q/Orbitrap/MS HRMS spectrum showing fragments of delphinidin-3-*O*-sambubioside (**47**).

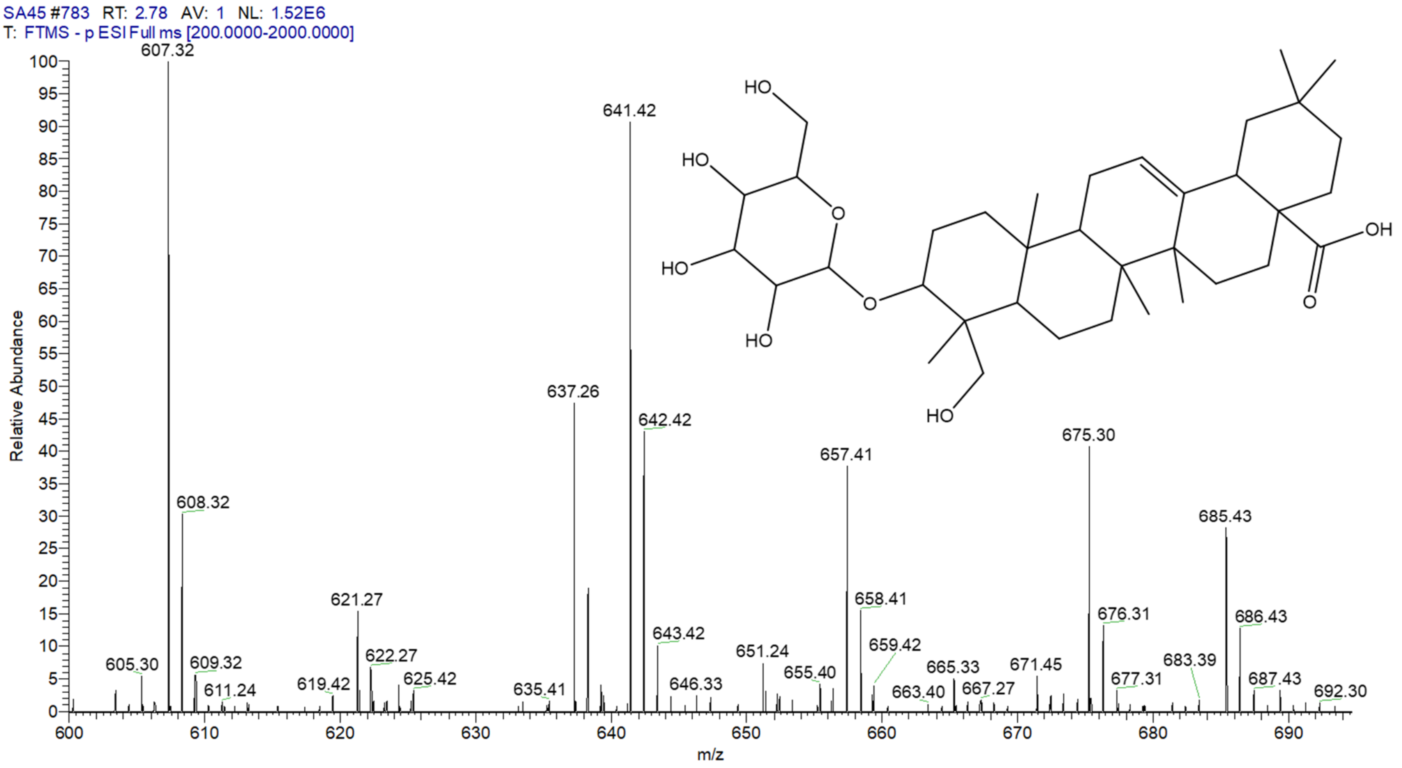

Figure S54. UHPLC–Q/Orbitrap/MS HRMS spectrum showing fragments of hederagenin base + *O*-AcetylHex (**48**).

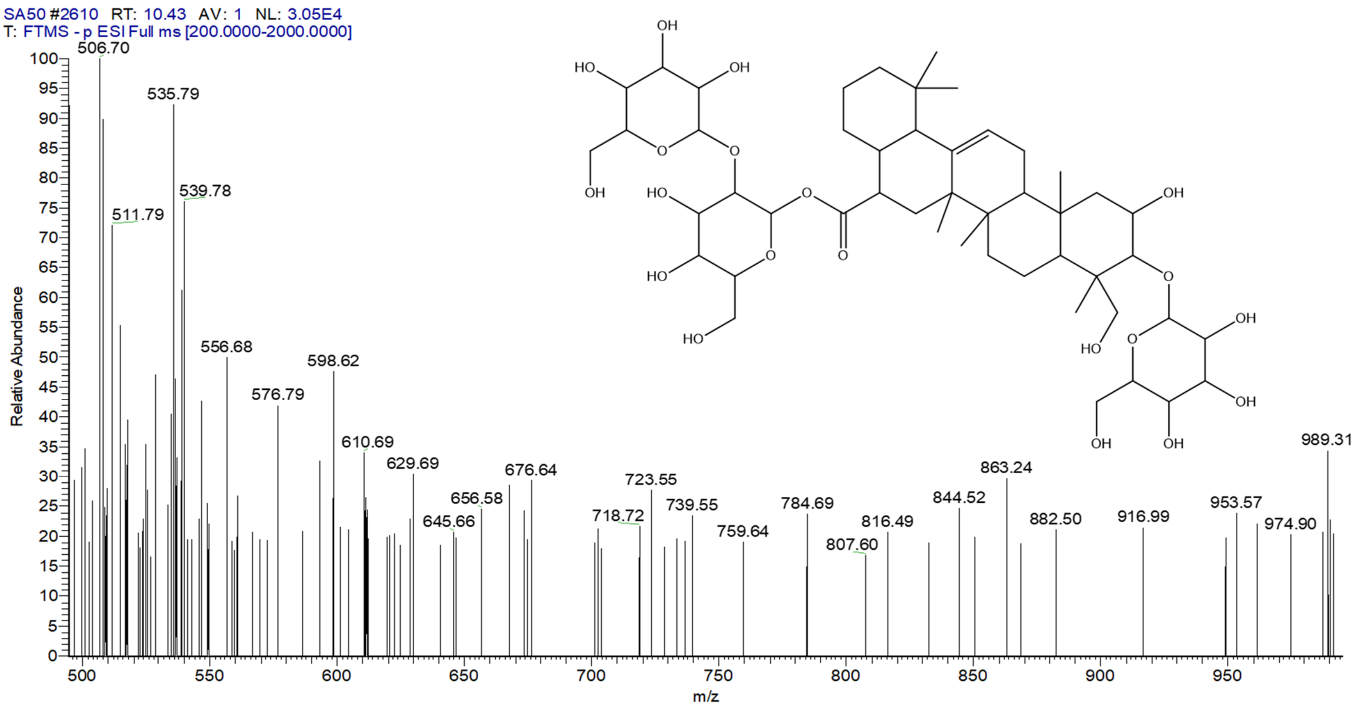

Figure S55. UHPLC–Q/Orbitrap/MS HRMS spectrum showing fragments of bayogenin base + *O*-Hex, *O*-Hex-Hex (**49**).

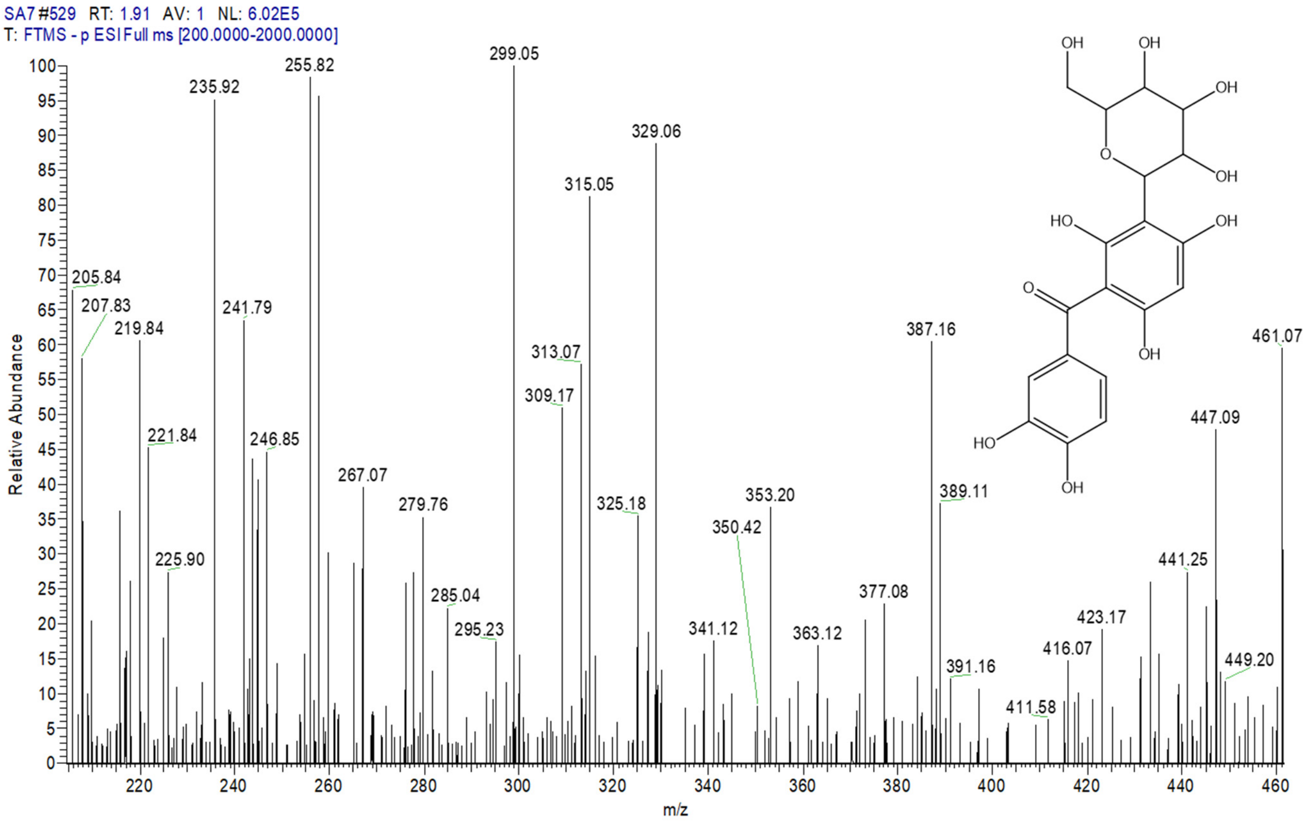

Figure S56. UHPLC–Q/Orbitrap/MS HRMS spectrum showing fragments of 3-glucosyl-2,3′,4,4′,6-pentahydroxybenzophenone (**50**).

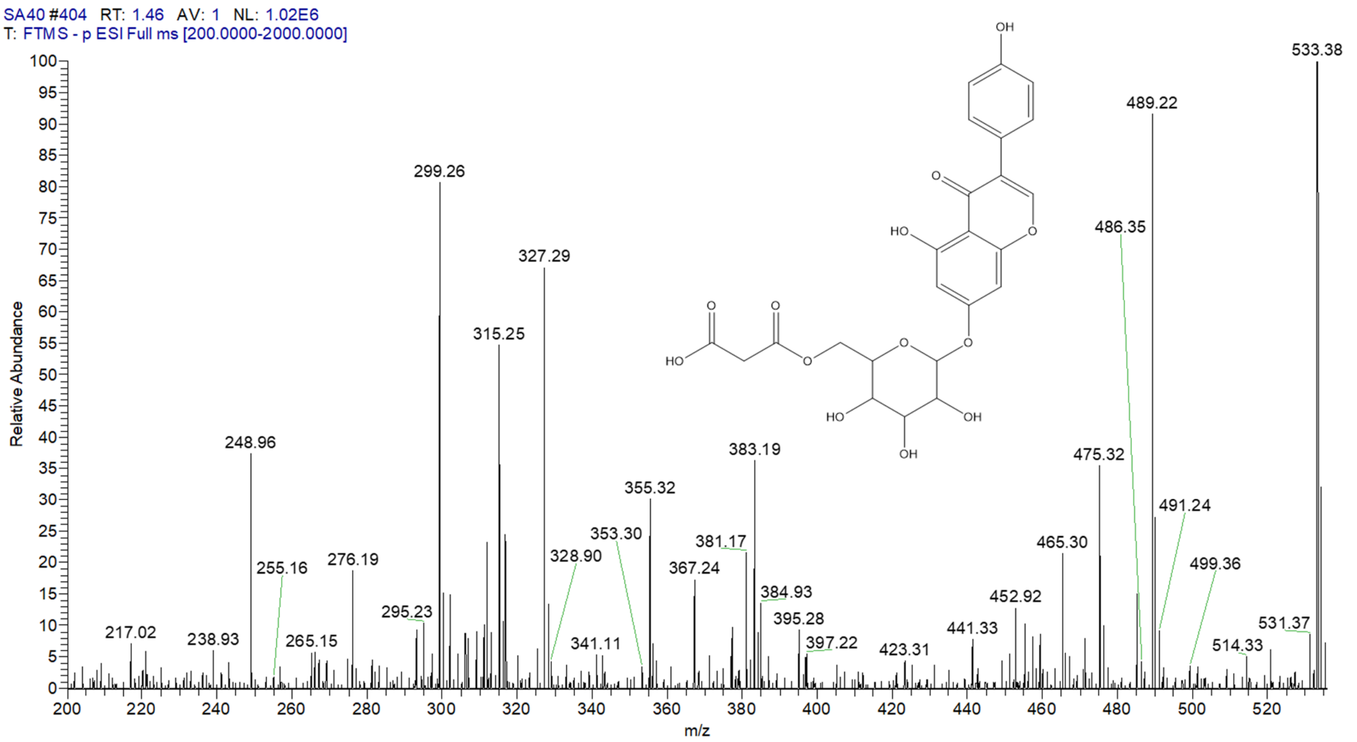

Figure S57. UHPLC–Q/Orbitrap/MS HRMS spectrum showing fragments of 6'' -*O*-malonylgenistin (**51**).

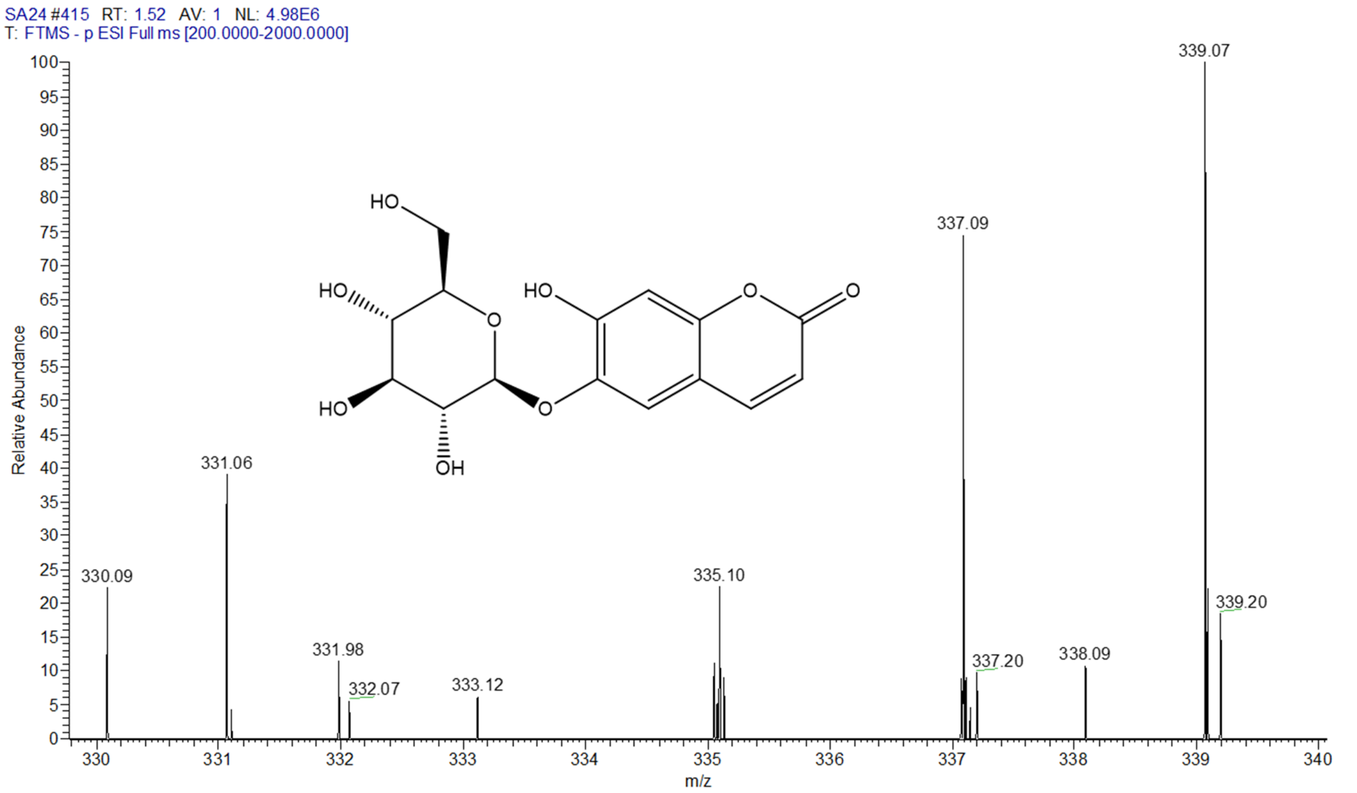

Figure S58. UHPLC–Q/Orbitrap/MS HRMS spectrum showing fragments of 6,7-dihydroxycoumarin-6-glucoside (Esculin) (**52**).

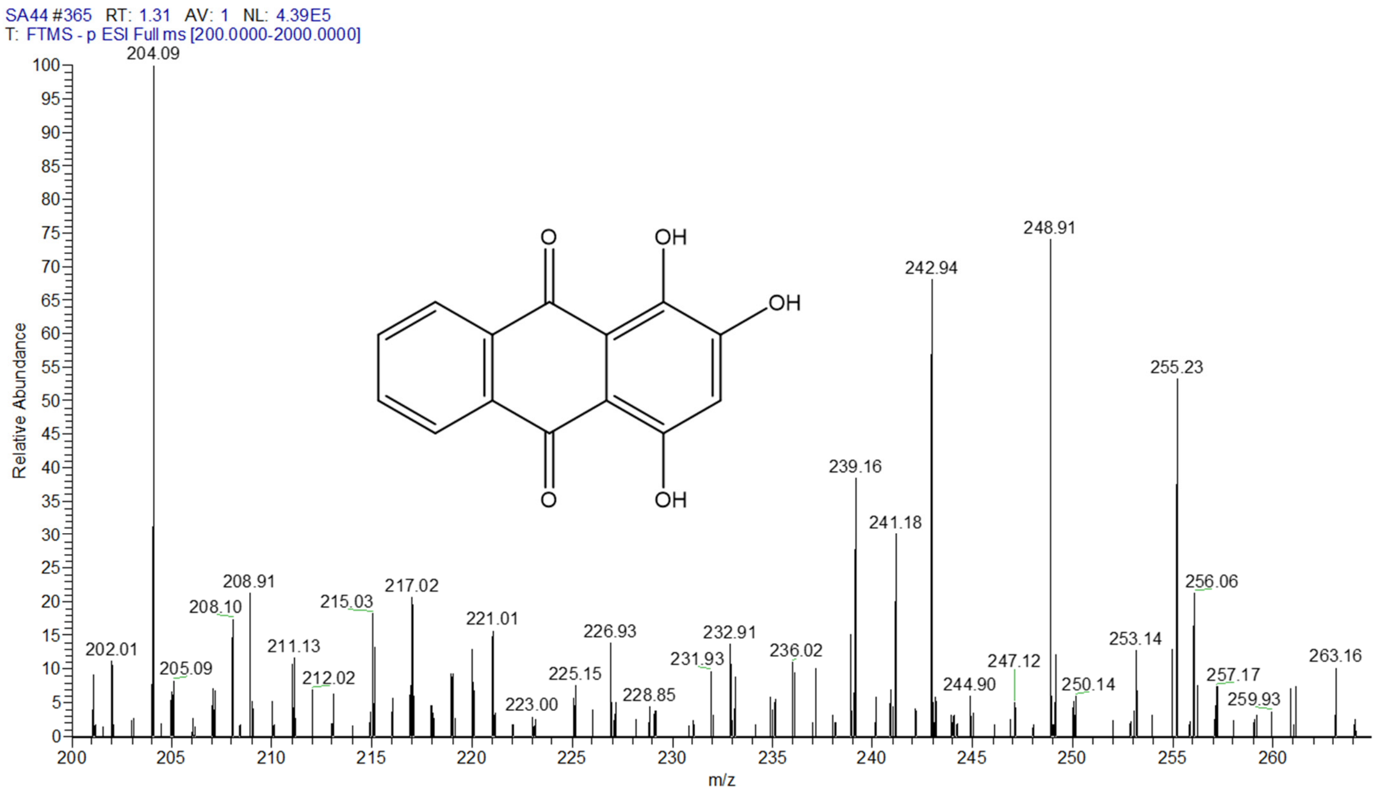

Figure S59. UHPLC–Q/Orbitrap/MS HRMS spectrum showing fragments of purpurin (**53**).

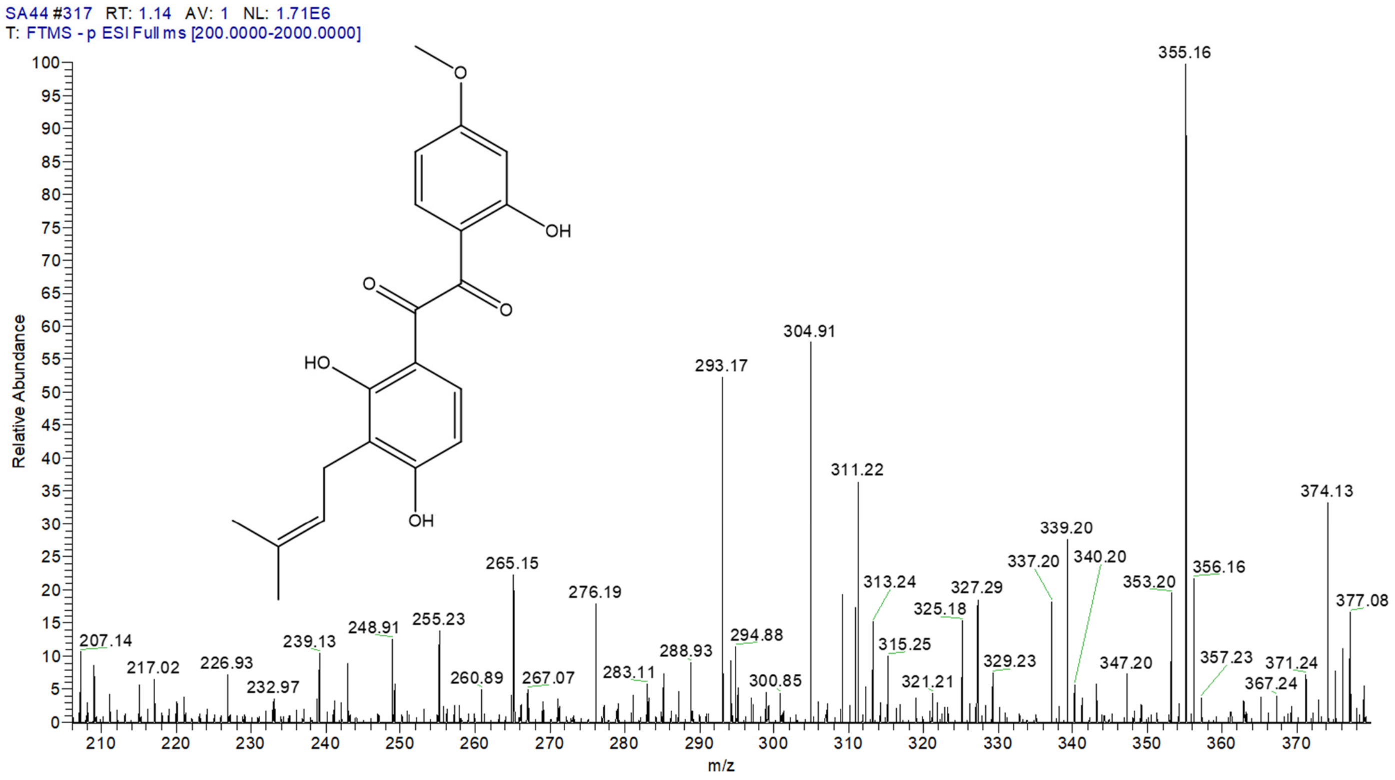

Figure S60. UHPLC–Q/Orbitrap/MS HRMS spectrum showing fragments of licoagrodione (**54**).

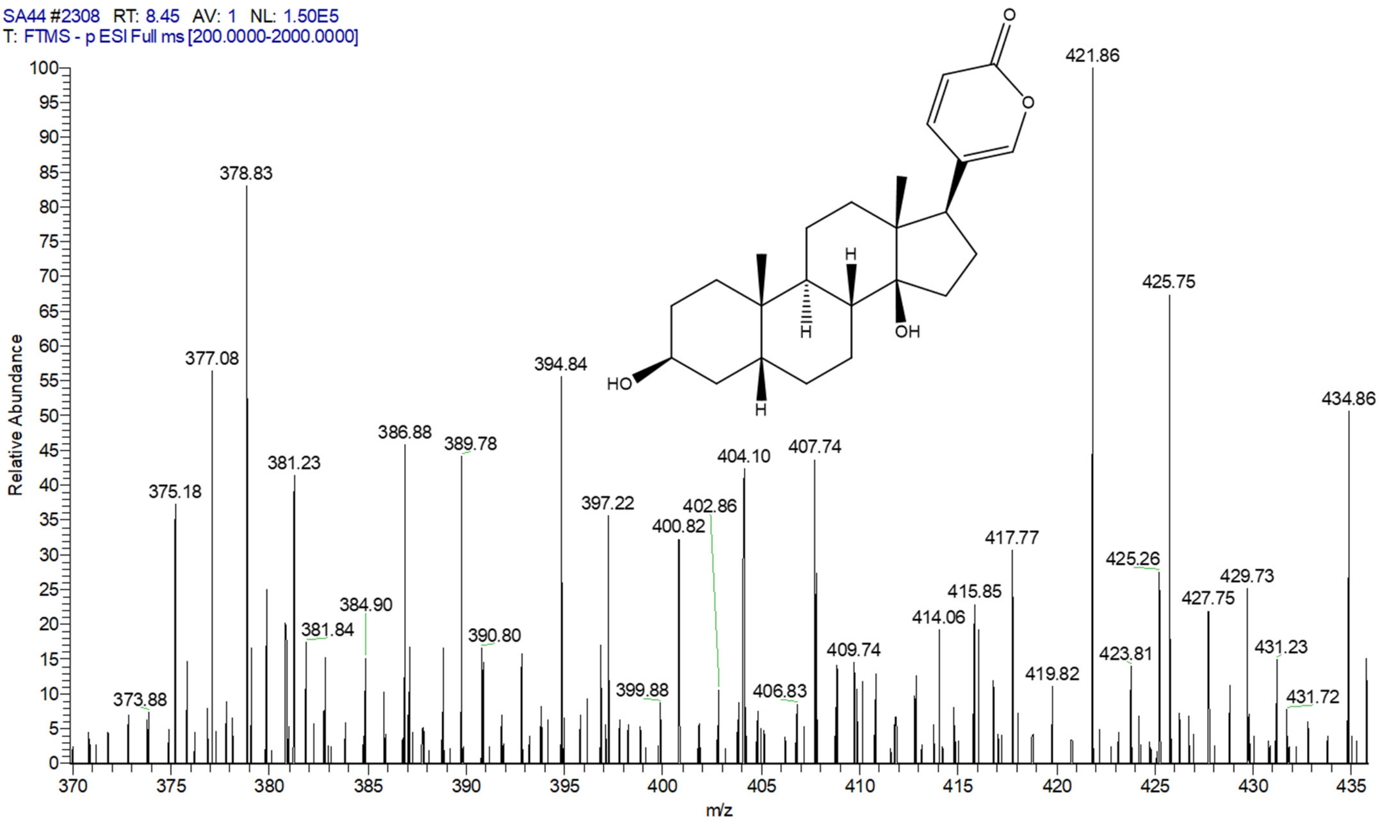

Figure S61. UHPLC–Q/Orbitrap/MS HRMS spectrum showing fragments of bufalin (**55**).

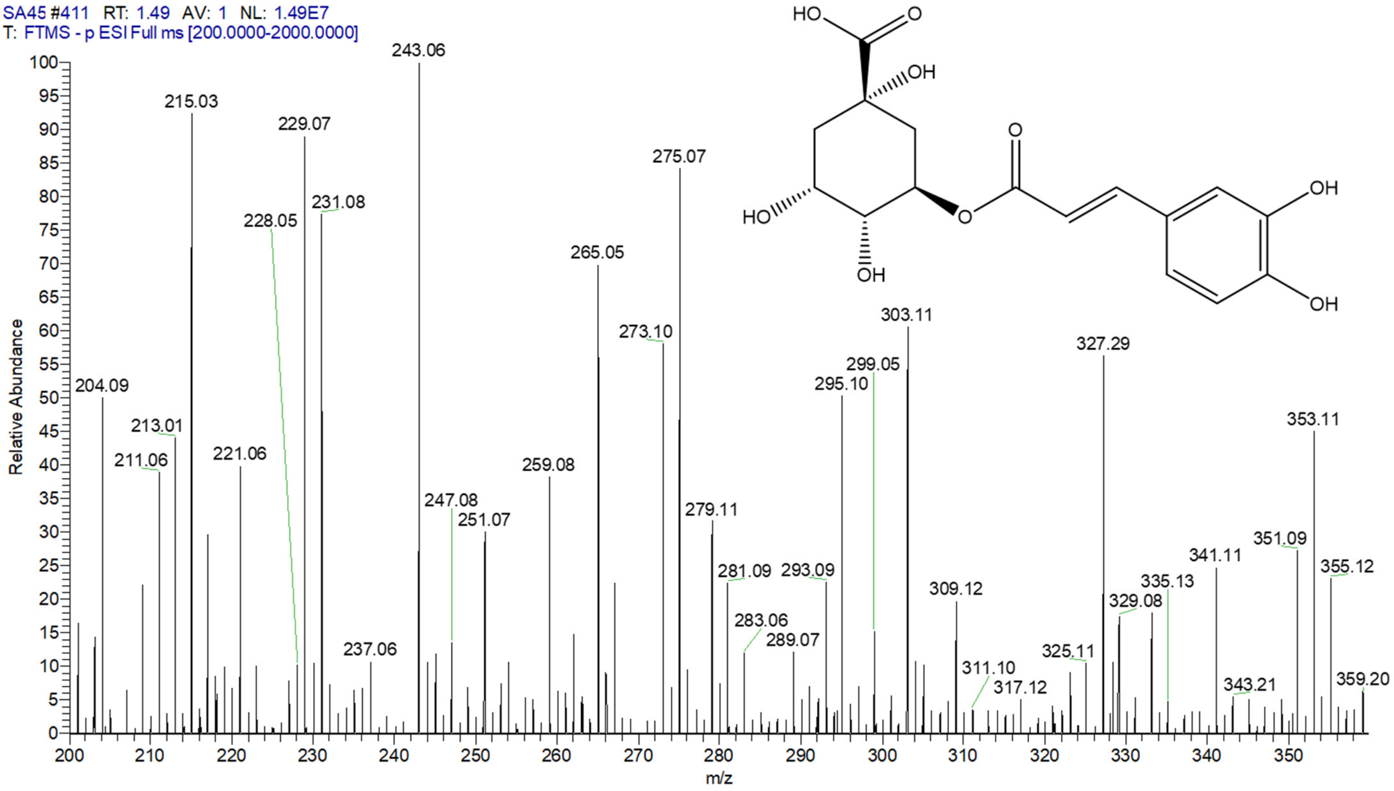

Figure S62. UHPLC–Q/Orbitrap/MS HRMS spectrum showing fragments of chlorogenic acid (**56**).

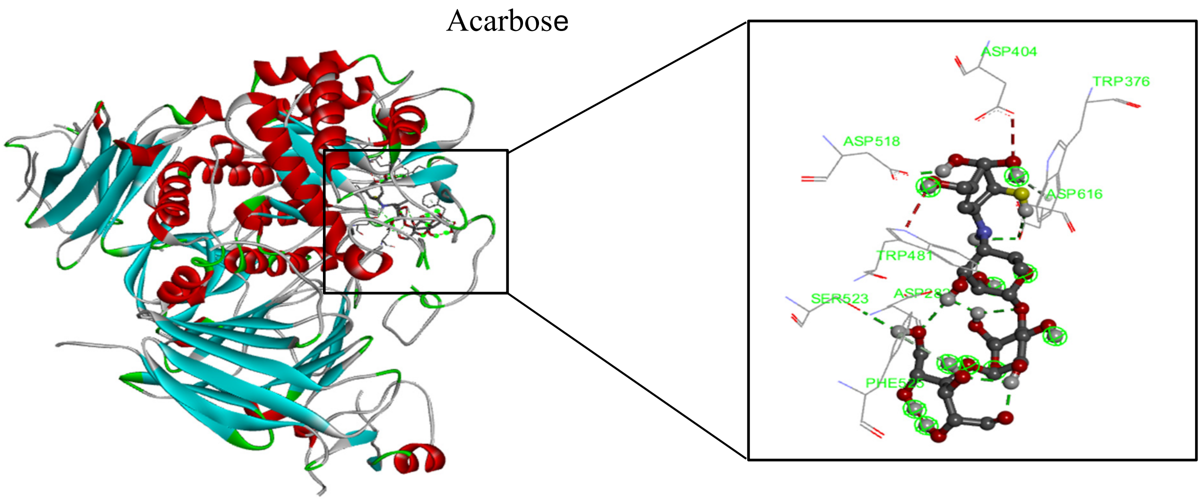
Acarbose
ASP404
TRP376
ASP518
ASP616
TRP481
SER523

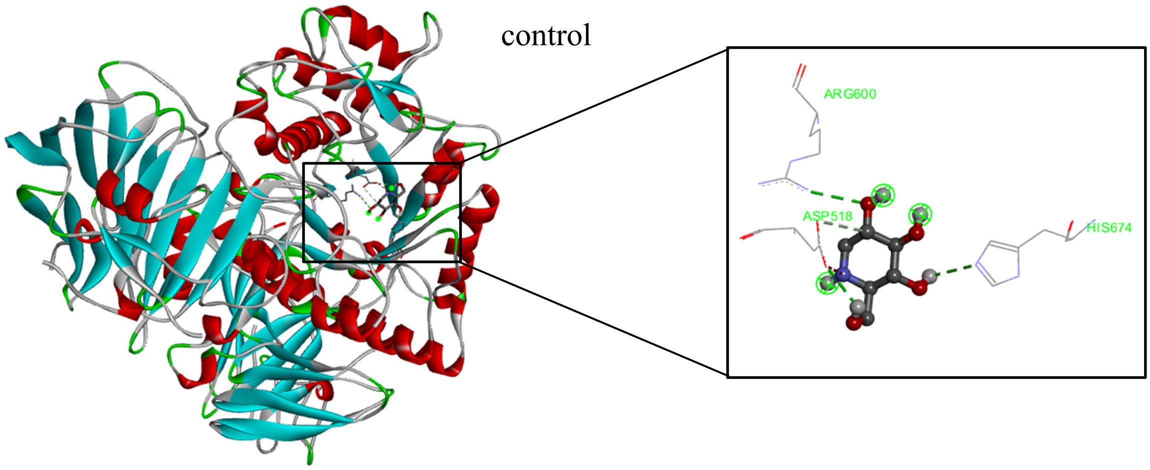
control
ARG600
ASP518
HIS674

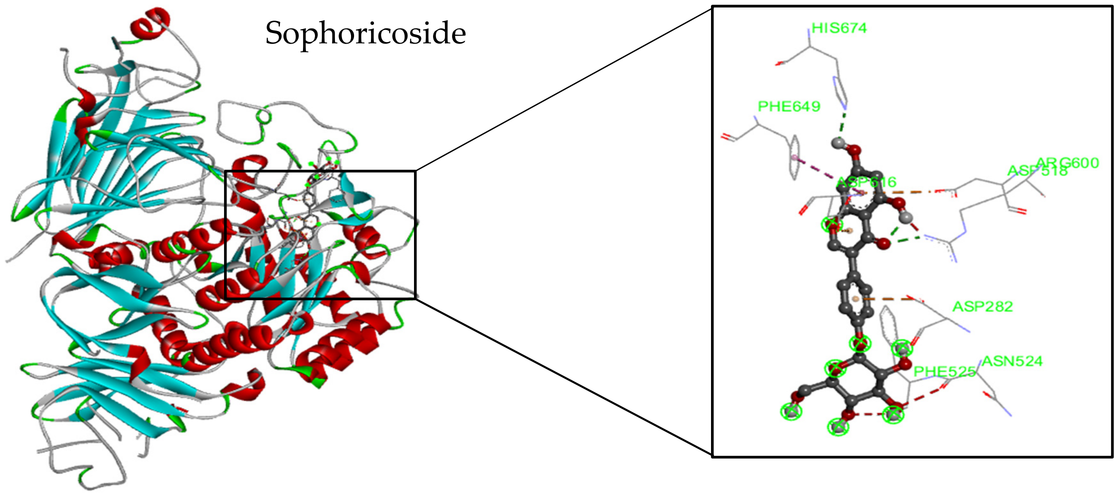
Sophoricoside
HIS674
PHE649
ARG600
ASP282
ASN524
PHE525

(-)-Epicatechin

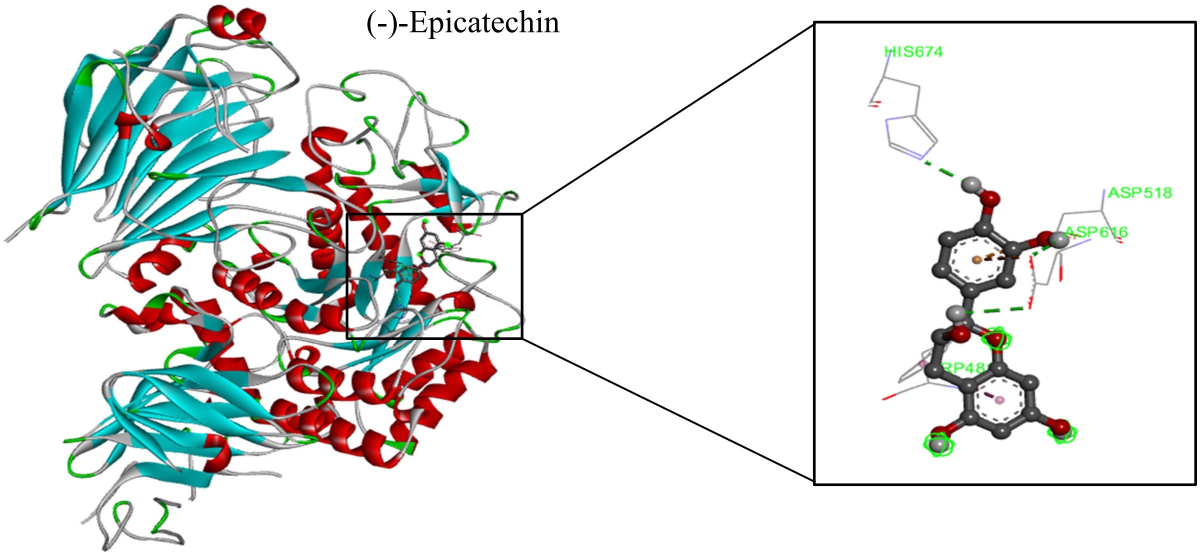

6-Gingerol

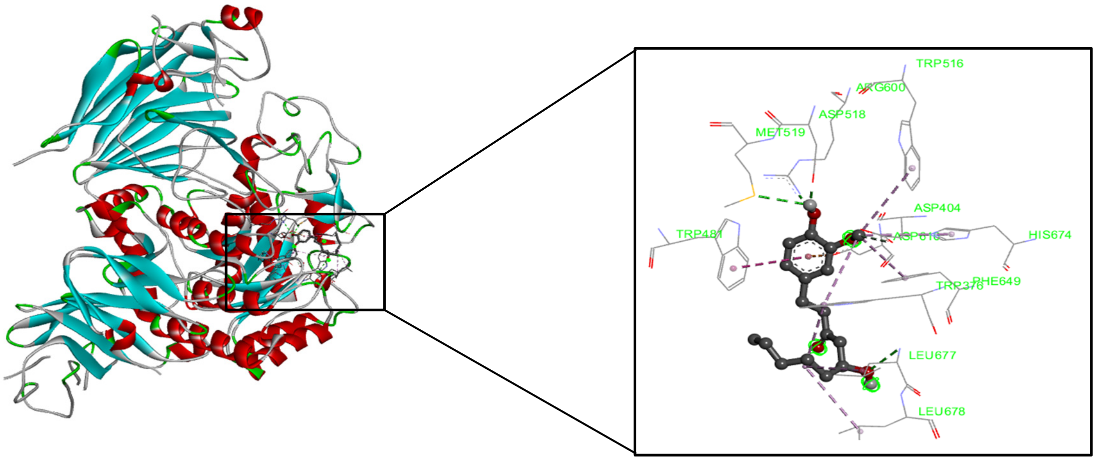

6-Paradol

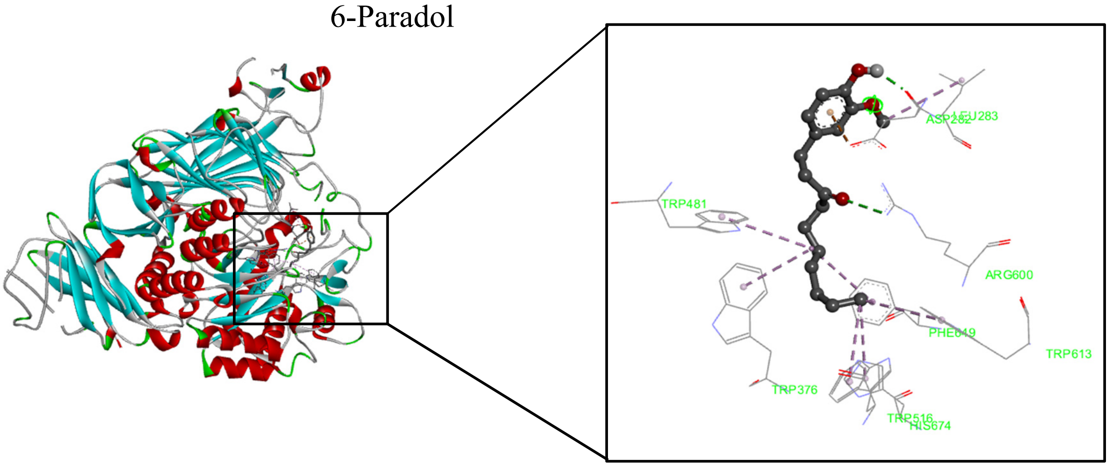

Ononin

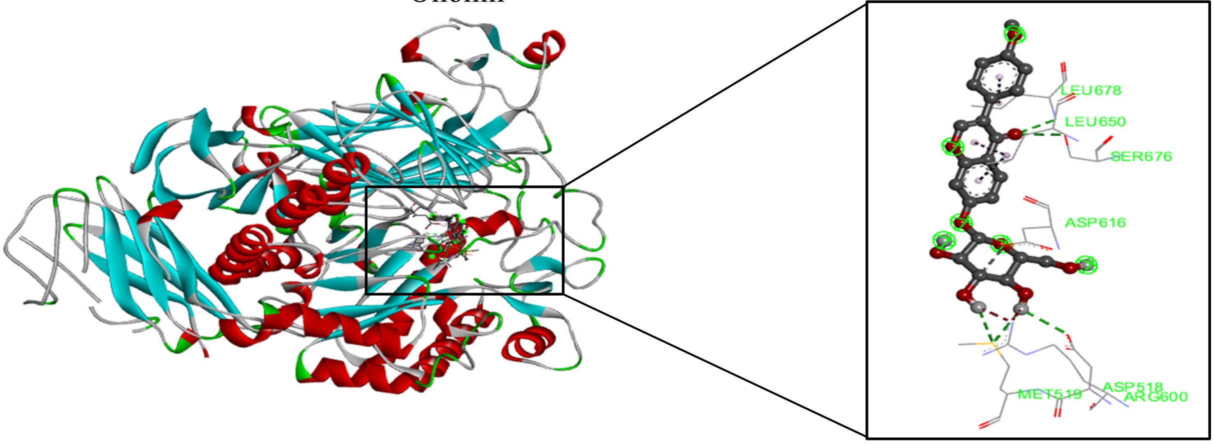

Figure S63. 3D visualisation for molecular docking against 5NN5 for the isolated compounds.
